# Application of chiral 2-isoxazoline for the synthesis of *syn*-1,3-diol analogs

**DOI:** 10.3762/bjoc.15.179

**Published:** 2019-08-01

**Authors:** Juanjuan Feng, Tianyu Li, Jiaxin Zhang, Peng Jiao

**Affiliations:** 1 Key Laboratory of Radiopharmaceuticals, College of Chemistry, Beijing Normal University, Beijing 100875, P.R. China

**Keywords:** β-hydroxy ketone, cycloaddition, 1,3-diol, isoxazoline, silyl nitronate

## Abstract

The asymmetric cycloaddition of TIPS nitronate catalyzed by “Cu(II)-bisoxazoline” gave the 2-isoxazoline product in 95% yield, which was converted into *tert*-butyl (3*S*,5*R*)-6-hydroxy-3,5-*O*-isopropylidene-3,5-dihydroxyhexanoate in 14 steps through a β-hydroxy ketone.

## Introduction

**Scheme 1:**
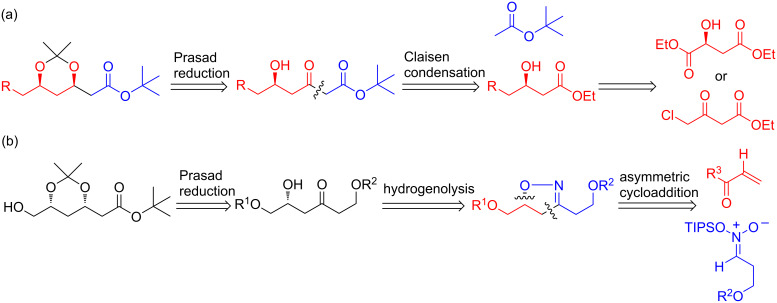
Accesses to *tert*-butyl 3,5-*O*-isopropylidene-3,5-dihydroxyhexanoates. (a) Previous methods using Claisen condensation. (b) Our new method using cycloaddition.

The chiral 1,3-diol structure is widespread in a broad spectrum of natural products [1–2]. (3*R*)-β-Hydroxy-δ-lactone or its open-ring equivalent (3*R*)-*syn*-3,5-dihydroxypentanoic acid, is a common structure in naturally occurring mevastatin (or compactin), lovastatin or closely related statins, and synthetic statins. Either the *syn* or *anti*-1,3-diol could be prepared from enantiomerically pure β-hydroxy ketones through β-hydroxy-directed carbonyl reduction following Evans’ [3] or Prasad’s [4–11] method. The Narasaka–Prasad reduction of a δ-hydroxy-β*-*keto esters derived from β-hydroxy esters [12–23] is widely used to prepare *tert*-butyl (3*R*)-3,5-*O*-isopropylidene-3,5-dihydroxyhexanoate (Scheme 1a) [24–37], which is a building block for synthetic statins [38–41], though enzymatic syntheses [42–48] of the chiral β-hydroxy-δ-lactone moiety or its equivalents, pioneered by Wong [42], is equally competitive. Here, we report the preparations of *tert*-butyl (3*S*,5*R*)-6-hydroxy-3,5-*O*-isopropylidene-3,5-dihydroxyhexanoate and related *syn*-1,3-diol analogs from a chiral 2-isoxazoline (Scheme 1b). This work is part of our continuous efforts in asymmetric syntheses and applications of chiral 2-isoxazolines [49–51].

## Results and Discussion

Our synthesis commenced with a chiral 3,5-disubstituted-2-isoxazoline **3** or **4**, which were prepared from silyl nitronate through an asymmetric 1,3-dipolar cycloaddition developed in our lab (Table 1) [49]. The synthesis of the triisopropylsilyl nitronate was initially attempted starting with 3-nitropropionic acid methyl ester but no desired product was observed. However, switching to 3-nitropropanol, protected as the THP ether, succeeded to prepare the required triisopropylsilyl nitronate. Then, the catalytic asymmetric cycloaddition gave the 2-isoxazolidine cycloadduct **1** in a high yield. In the light of our previous ligand screening results [49], two bisoxazolines with an isopropyl (ligand B) or *tert*-butyl group (ligand A) were tested. Optimization of the conditions established that 26 mol % of ligand B together with 20 mol % Cu(OTf)_2_ in anhydrous CH_2_Cl_2_ catalyzed the cycloaddition between *N*-acryloyl-1,3-oxazolidin-2-one and the silyl nitronate at −50 °C to give **1** in 95% isolated yield, which subsequently generated 3,5-disubstituted isoxazoline **4** in 80% ee. Decreasing the amount of the chiral Lewis acid catalyst led to a decrease of both the ee and the yield. Desilylation of the 2-isoxazolidine **1** was effected in CHCl_3_ using catalytic amounts of *p*-toluenesulfonic acid (PTSA). Though the yield of the in situ-generated 2-isoxazoline **2** bearing the 1,3-oxazolidin-2-one auxiliary was perfect, purification of **2** by silica gel chromatography was problematic due to decomposition. No pure product was isolated from crude **2** by chromatography on silica gel. Decomposition occurred to a compound similar to **2**, in which the 3-substituent was CH_2_OH [49]. To overcome this problem, the crude reaction mixture containing **2** and PTSA was concentrated before excess Et_3_N was added followed by CH_3_OH as the solvent. These operations removed the 1,3-oxazolidin-2-one auxiliary while preserving the THP group, and afforded the corresponding methyl ester **3** (Table 1), which was stable and could be subjected to silica gel chromatography. Compound **4** was used to determine the stereoselectivity of the cycloaddition step as well as for oxidation.

**Table 1:** Optimization of the conditions for the asymmetric cycloaddition.

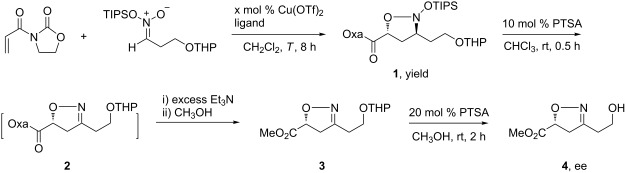					

Entry	Ligand	x mol % Cu	*T* (°C)	Yield (%) (**1**)	ee (%) (**4**)

1	A	10	−50	25	18
2	A	20	−50	75	72
3	B	10	−50	70	66
4	B	20	−50	95	80
5	B	20	−40	78	78
6	B	20	−60	84	80

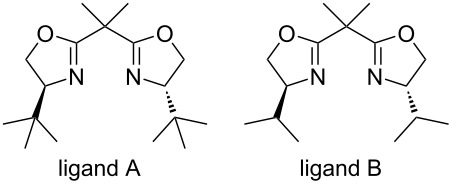

**Scheme 2:**
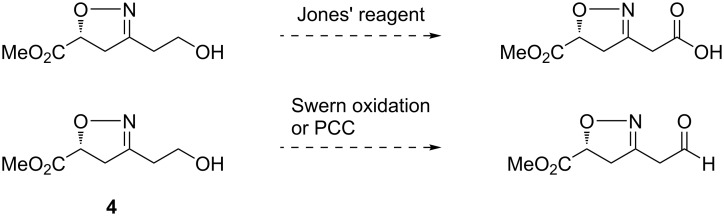
Attempted oxidations of **4**.

Oxidation of the 2-isoxazoline **4** with Jones’ reagent gave a complicated mixture, in which the desired carboxylic acid was not observed (Scheme 2). The stepwise oxidation of the free hydroxy to the carboxy group via intermediary aldehyde was then examined. Swern or pyridinium chlorochromate (PCC) oxidation of **4** also gave a complicated mixture without the desired aldehyde detected. These failed reactions indicated that the 2-isoxazoline moiety could not survive oxidation conditions. Based on this assumption, the corresponding silyl nitronate from 3-nitropropanal or its acetal were not tried for cycloaddition.

**Scheme 3:**
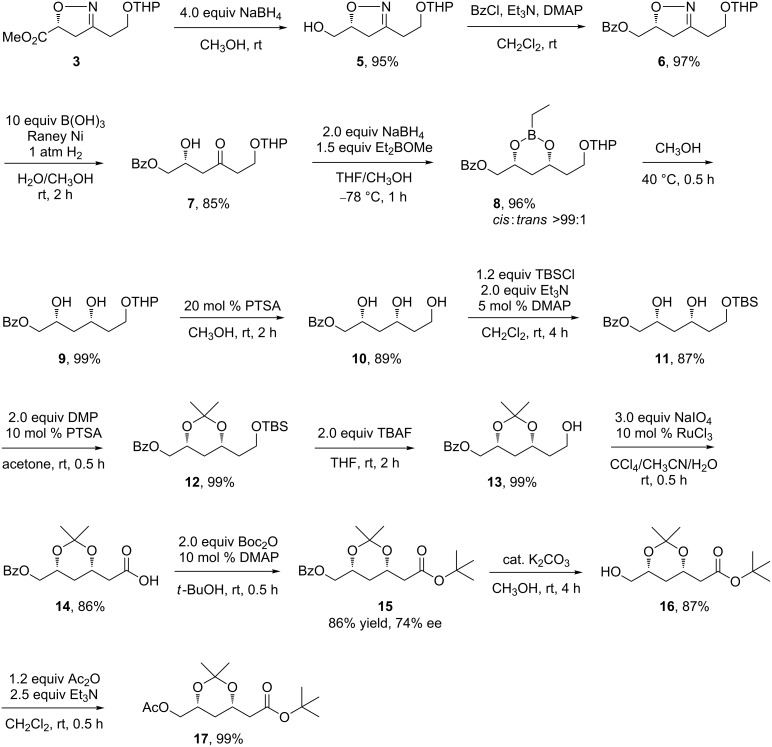
Preparations of **16** and related *syn*-1,3-diol compounds.

**Scheme 4:**
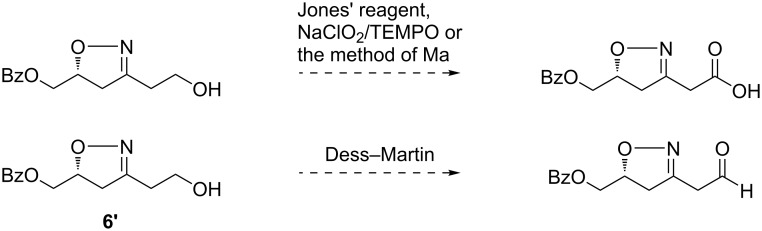
Attempted oxidations of **6'**.

We then set to liberate the β-hydroxy ketone synthon by ring opening of the isoxazoline **3** (Scheme 3). Raney-Ni-catalyzed hydrogenolysis in the presence of boronic acid had been widely utilized to disconnect the N–O bond as well as to hydrolyze the resulting imine into a ketone [52]. We applied this method to deprotect the isoxazoline **3**. However, the desired β-hydroxy ketone was never obtained. In one instance, the methyl ketone from a retro-aldol reaction of the desired β-hydroxy ketone was observed. In our experience, the hydrogenolysis of a 2-isoxazoline having a 5-ester group was troublesome. Thus, the 5-ester group was reduced with NaBH_4_ to give **5**. The hydroxy group was subsequently protected with benzoyl (Scheme 3), which also worked as a chromophore facilitating HPLC analysis. Afterwards, we tried oxidations once again. After removal of THP from **6**, the resulting compound **6'** was subjected to oxidation with various reagents (Scheme 4) [53–55]. The expected carboxylic acid or aldehyde was not observed, which further verified the intolerance exemplified in Scheme 2. These results prompted us to try the oxidation in a later stage.

When **6** was subjected to Raney-Ni-catalyzed hydrogenolysis, the desired β-hydroxy ketone **7** was obtained in 85% yield (Scheme 3). Under the weakly acidic conditions, the THP group survived. Next, a Narasaka–Prasad reduction [4–11] of **7** using Et_2_BOMe and NaBH_4_ at −78 °C gave stable ethylboronate **8** in 96% yield. Several ethylboronate compounds have been reported [9–11,56–62]. From **8** to **9**, no H_2_O_2_ treatment was necessary. Rotary evaporation of **8** with CH_3_OH at ca. 40 °C easily removed the ethylborane group. Removal of THP in **9** delivered a 1,3,5-trihydroxy compound **10**. In another way, **10** could be prepared by treating **8** with PTSA in CH_3_OH at rt. NMR spectra of **8**–**10** exhibited only one set of signals corresponding to the *syn*-dihydroxy products, indicating an extra high diastereoselectivity (*syn*:*anti* >99:1) during the reduction. To unambiguously determine the diastereomeric ratio, the *anti*-1,3-diol corresponding to **10** was prepared from **7** by RuCl_3_–PPh_3_-catalyzed hydrogenation [63–64]. However, the two diastereomers had identical proton NMR spectra.

The terminal hydroxy group of **10** was protected with TBS [65–69] and the *syn*-hydroxy groups subjected to acetonization using PTSA and dimethoxypropane (DMP) to give **12** in 86% total yield [70]. Treatment of **12** with TBAF again liberated the terminal hydroxy group for further oxidation. RuCl_3_-catalyzed oxidation of **13** with NaIO_4_ yielded the carboxylic acid **14** in 86% yield [70], which was reacted with Boc_2_O to get the *tert*-butyl ester **15** [26,43,71]. The ee of **15** was determined as 74%. The racemic sample of **15** was prepared from racemic diethyl malate following known methods [26–27]. Finally, K_2_CO_3_-catalyzed methanolysis gave **16** in 87% yield [26–27]. The absolute stereochemistry of **16** was confirmed by crystal structure analysis [72] and the specific rotation [28] of **17**. Centimeter-long prismatic single crystals of **17** were obtained by slow evaporation of a petroleum solution.

**Scheme 5:**
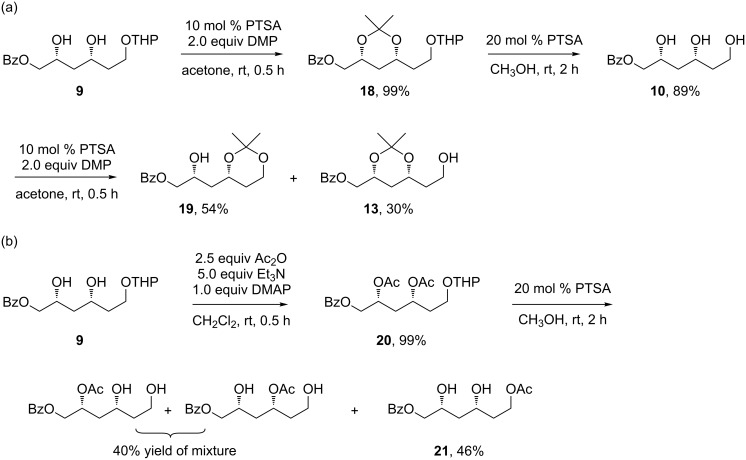
Attempted selective protections of internal 1,3-hydroxy groups: (a) acetonizations of 1,3-diols; (b) removal of co-existing Ac and THP on hydroxy groups.

Starting from **9**, we tested several reactions in order to selectively protect the internal hydroxy groups (Scheme 5). Though not fruitful, these results deserve some comments. The PTSA-catalyzed acetonization of **9** using 2.0 equiv DMP gave the acetonide **18** in a quantitative yield. Treating **18** with a catalytic amount of PTSA in methanol gave **10**, with the protecting groups removed except benzoyl. PTSA-catalyzed acetonization of **10** using 2.0 equiv DMP gave a mixture of two acetonides **19** and **13**, which are separable by silica gel chromatography (Scheme 5a). In another trial (Scheme 5b), acylation of the two hydroxy groups in **9** yielded **20** in a quantitative yield. PTSA-catalyzed removal of THP in **20** in methanol did occur. However, concomitant monodeacylation as well as further an acyl-transfer reaction also took place, resulting in a mixture. These results indicated THP, isopropylidene or Ac protection to primary or secondary hydroxy groups did not well tolerate PTSA-catalyzed methanolysis.

## Conclusion

In conclusion, we synthesized *tert*-butyl (3*S*,5*R*)-6-hydroxy-3,5-*O*-isopropylidene-3,5-dihydroxyhexanoate (**16**), which is enantiomeric to a key intermediate for atorvastatin, from a chiral 2-isoxazoline (**3**). The β-hydroxy ketone **7** obtained from **3** could be easily converted into several *syn*-1,3-diol analogs, demonstrating the usefulness of chiral 2-isoxazolines.

## Experimental

**1**: To a dry Schlenk tube were added Cu(OTf)_2_ (144 mg, 0.4 mmol), chiral bisoxazoline B (139 mg, 0.52 mmol) and anhydrous CH_2_Cl_2_ (4 mL) under N_2_. After stirring at room temperature for 2 h, a clear solution had formed, which was cooled to −50 °C and *N*-acryloyl-1,3-oxazolidin-2-one (282 mg, 2 mmol) was added. After stirring for 30 min, a solution of the silyl nitronate (3.0 mmol) in anhydrous CH_2_Cl_2_ (6 mL) was added. The mixture was stirred for 8 h at −50 °C and monitored by TLC. After the reaction was completed, the product was purified by silica gel chromatography. Yellow oil (923 mg, 95% yield); *R*_f_ 0.40 (1:1 hexanes/AcOEt); ^1^H NMR (400 MHz, CDCl_3_) δ 5.77–5.74 (m, 1H, CH_2_C*H*O), 4.53 (s, 1H, OC*H*O), 4.44 (t, *J* = 8.0 Hz, 2H, C*H*_2_O), 4.03–3.99 (m, 2H, C*H*_2_O), 3.79–3.74 (m, 2H, OC*H*_2_CH_2_), 3.47–3.37 (m, 3H, NC*H* and NC*H*_2_), 2.75–2.66 (m, 1H, CHC*H*_2_CH), 2.31–2.27 (m, 1H, CHC*H*_2_CH), 2.17–2.12 (m, 1H, C*H*_2_CH_2_), 1.84–1.79 (m, 2H, CH_2_C*H*_2_ and CH_2_CH_2_C*H*_2_), 1.68–1.49 (m, 6H, C*H*_2_C*H*_2_C*H*_2_), 1.24–1.15 (m, 3H, SiCH), 1.07–1.01 (m, 18H, SiCH(C*H*_3_)_2_); ^13^C NMR (100 MHz, CDCl_3_) δ 170.8, 153.1, 98.9, 98.9, 77.4, 77.2, 69.9, 69.8, 65.2, 62.8, 62.5, 62.3, 42.6, 35.6, 35.5, 30.7, 30.6, 29.9, 29.8, 25.5, 19.6, 19.5, 18.1, 18.0, 12.2; IR (cm^−1^): 3544, 2942, 2867, 2725, 2249, 1780, 1704, 1464, 1386, 1275, 1133, 1035, 883, 806, 677; ESIMS (*m*/*z*): [M + Na]^+^ calcd for C_23_H_42_N_2_O_7_Si, 509.2659; found, 509.2659.

**3**: To a solution of **1** (0.86 g, 1.78 mmol) in CHCl_3_ (15 mL) was added PTSA (31 mg, 0.178 mmol) at 0 °C. The mixture was allowed to warm to room temperature and stirred until complete consumption of the starting material (0.5 h). Vacuum was applied to remove the solvent before Et_3_N (5 mL) was added. After stirring for 5 min, methanol (30 mL) was added and the mixture was stirred overnight at room temperature. The crude product was purified by column chromatography. Yellow oil (0.41 g, 89% yield); *R*_f_ 0.42 (1:1 hexanes/AcOEt); ^1^H NMR (400 MHz, CDCl_3_) δ 4.92–4.87 (m, 1H, OC*H*CO), 4.51–4.50 (m, 1H, OC*H*O), 3.88–3.82 (m, 1H, C*H*_2_O), 3.75–3.71 (m, 1H, C*H*_2_O), 3.68 (s, 3H, C*H*_3_), 3.56–3.50 (m, 1H, C*H*_2_O), 3.42–3.39 (m, 1H, C*H*_2_O), 3.24–3.31 (m, 1H, CHC*H*_2_CH), 2.62–2.54 (m, 1H, CH_2_C*H*_2_CH), 1.74–1.44 (m, 6H, C*H*_2_C*H*_2_C*H*_2_); ^13^C NMR (100 MHz, CDCl_3_) δ 171.0, 156.9, 99.0, 98.9, 64.4, 64.3, 62.5, 62.4, 52.6, 41.6, 30.6, 27.9, 25.4, 19.6, 19.5; IR (cm^−1^): 3481, 2950, 2873, 2852, 2657, 1756, 1738, 1734, 1628, 1456, 1436, 1367, 1354, 1201, 1134, 1034, 869, 814, 752, 740; ESIMS (*m*/*z*): [M + H]^+^ calcd for C_12_H_19_NO_5_, 258.1341; found, 258.1340.

## Supporting Information

File 1 Experimental procedures and characterization data.

File 2 Crystallographic data for **17**.
