# Supplementary material for: Application of chiral 2-isoxazoline for the synthesis of syn-1,3-diol analogs
Source: Beilstein J Org Chem. 2019 Aug 1;15:1840–7. doi: 10.3762/bjoc.15.179 (PMC6693402; doi:10.3762/bjoc.15.179)
Supplement: File 1 — Experimental procedures and characterization data. [file Beilstein_J_Org_Chem-15-1840-s001.pdf]

BEILSTEIN JOURNAL OF ORGANIC CHEMISTRY

Supporting Information

for

Application of chiral 2-isoxazoline for the synthesis of *syn*-1,3-diol analogs

Juanjuan Feng, Tianyu Li, Jiaxin Zhang and Peng Jiao

Beilstein J. Org. Chem. **2019,** *15,* 1840–1847. doi:10.3762/bjoc.15.179

Experimental procedures and characterization data

License and Terms: This is a supporting information file under the terms of the Creative Commons Attribution License (http://creativecommons.org/licenses/by/4.0). Please note that the reuse, redistribution and reproduction in particular requires that the authors and source are credited.
The license is subject to the *Beilstein Journal of Organic Chemistry* terms and conditions: (https://www.beilstein-journals.org/bjoc)

Table of contents

General information S2

1. Preparation of compounds **1–21** S2

2. Preparation of the racemic samples of compounds **4** and **15** S16

3. Crystallographic data of compound **17** S23

NMR spectra S25

HPLC charts S76

General information

All glassware for reactions using anhydrous solvents were dried under high vacuum (<0.1 torr) using a heat gun. General Schlenk techniques were applied for addition and transfer operations. Commercial reagents and solvents were used as received unless otherwise noted. THF was distilled over sodium benzophenone ketyl under N_2. CH_2Cl_2 was distilled over CaH_2 under N_2. Thin-layer chromatography was performed on precoated silica gel (0.2–0.25 mm thickness) plates with fluorescent indicator 254 nm. The plate was visualized with 254 nm UV lamp, PMA or $KMnO_4$ stain. Column chromatography was performed on 200–300 mesh silica gel.

^{1}H NMR and ^{13}C NMR spectra were recorded at 400 (or 600) MHz and 100 (or 150) MHz, respectively. Chemical shifts of ^{1}H NMR and ^{13}C NMR were referenced to TMS (δ = 0) and chloroform (δ = 77.16), respectively. Chemical shifts of ^{11}B NMR (128 MHz) were referred to $BF_3 \cdot Et_2O$ (δ = 0). The following abbreviations were used to denote the multiplicity of each peak: s (singlet), d (doublet), t (triplet), q (quartet), dd (doublet of doublets), m (multiplet). HPLC was performed at room temperature. Specific rotation was measured using the 589 nm D-line of sodium lamp and a quartz cell with 10 cm path length. The X-ray diffraction experiment was conducted using CuKα radiation.

1. Preparation of compounds 1–21

N-{(3*S*,5*R*)-[3-(2-(2-Tetrahydropyranyloxy)ethyl)-2-triisopropylsilyloxy-5-isoxazolidinyl]carbonyl}-1,3-oxazolidin-2-one (1)

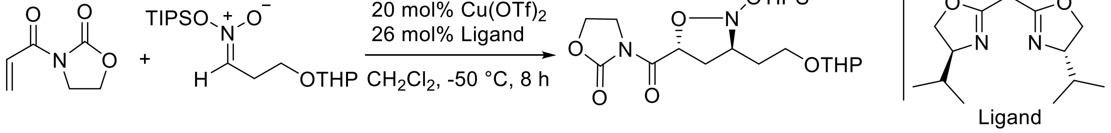

To a dry Schlenk tube were added $Cu(OTf)_2$ (144 mg, 0.4 mmol), chiral bisoxazoline (139 mg, 0.52 mmol) and anhydrous CH_2Cl_2 (4 mL) under N_2. After stirring at room temperature for 2 h, a clear solution was formed, which was cooled to −50 °C and *N*-acryloyl-1,3-oxazolidin-2-one (282 mg, 2 mmol) was added. After stirring for 30 min, a solution of the silyl nitronate (3.0 mmol) in anhydrous CH_2Cl_2 (6 mL) was added. The mixture was stirred for 8 h at −50 °C and monitored by TLC. After

the reaction was completed, the product was purified by silica gel chromatography.

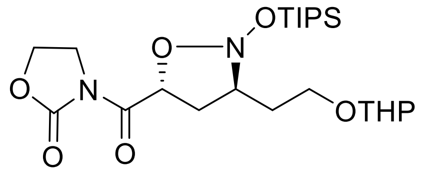

1: Yellow oil (923 mg, 95% yield), R_f = 0.40 (1:1 hexanes/AcOEt). ^{1}H NMR (400 MHz, $CDCl_3$) δ: 5.77–5.74 (m, 1H, CH_2C**H**O), 4.53 (s, 1H, OC**H**O), 4.44 (t, *J* = 8.0 Hz, 2H, C**H**$_2$O), 4.03–3.99 (m, 2H, C**H**$_2$O), 3.79–3.74 (m, 2H, OC**H**$_2$$CH_2$), 3.47–3.37 (m, 3H, NC**H** and NC**H**$_2$), 2.75–2.66 (m, 1H, CHC**H**$_2$CH), 2.31–2.27 (m, 1H, CHC**H**$_2$CH), 2.17–2.12 (m, 1H, C**H**$_2$$CH_2$), 1.84–1.79 (m, 2H, CH_2C**H**$_2$ and CH_2CH_2C**H**$_2$), 1.68–1.49 (m, 6H, C**H**$_2$C**H**$_2$C**H**$_2$), 1.24–1.15 (m, 3H, SiCH), 1.07–1.01 (m, 18H, SiCH(C**H**$_3$)$_2$); ^{13}C NMR (100 MHz, $CDCl_3$) δ: 170.8, 153.1, 98.9, 98.9, 77.4, 77.2, 69.9, 69.8, 65.2, 62.8, 62.5, 62.3, 42.6, 35.6, 35.5, 30.7, 30.6, 29.9, 29.8, 25.5, 19.6, 19.5, 18.1, 18.0, 12.2; IR (cm^{-1}): 3544, 2942, 2867, 2725, 2249, 1780, 1704, 1464, 1386, 1275, 1133, 1035, 883, 806, 677; MS (ESI): calculated for $C_{23}H_{42}N_2O_7Si$ $[M+Na]^+$ 509.2659, found 509.2659.

{(5*R*)-[3-(2-(2-Tetrahydropyranyloxy)ethyl)]-2-isoxazolin-5-yl}carboxylic acid methyl ester (3)

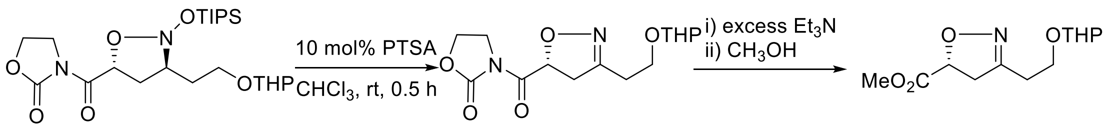

To a solution of **1** (0.86 g, 1.78 mmol) in $CHCl_3$ (15 mL) was added PTSA (31 mg, 0.178 mmol) at 0 °C. The mixture was allowed to warm to room temperature and stirred until complete consumption of the starting material (0.5 h). Vacuum was applied to remove the solvent before Et_3N (5 mL) was added. After stirring for 5 min, methanol (30 mL) was added and the mixture stirred overnight at room temperature. The crude product was purified by column chromatography.

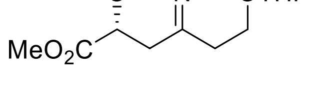

3: Yellow oil (0.41 g, 89% yield), R_f= 0.42 (1:1 hexanes/ AcOEt). ^{1}H NMR (400 MHz, $CDCl_3$) δ: 4.89 (dd, *J* = 10.2, 7.9 Hz, 1H, OC**H**CO), 4.51–4.50 (m, 1H, OC**H**O), 3.88–3.82 (m, 1H, C**H**$_2$O), 3.75–3.71 (m, 1H, C**H**$_2$O), 3.68 (s, 3H, C**H**$_3$), 3.56–3.50 (m, 1H, C**H**$_2$O), 3.42–3.39 (m, 1H, C**H**$_2$O), 3.24–3.21 (m, 2H, CHC**H**$_2$CH), 2.62–2.54 (m, 2H, CH_2C**H**$_2$CH), 1.74–1.44 (m, 6H, C**H**$_2$C**H**$_2$C**H**$_2$); ^{13}C NMR (100 MHz, $CDCl_3$) δ: 171.0, 156.9, 99.0, 98.9, 64.4, 64.3,62.5, 62.4, 52.6, 41.6, 30.6, 27.9, 25.4, 19.6, 19.5; IR (cm^{-1}): 3481, 2950, 2873, 2852, 2657, 1756, 1738, 1734, 1628, 1456,

1436, 1367, 1354, 1201, 1134, 1034, 869, 814, 752, 740; MS (ESI): calculated for $C_{12}H_{19}NO_5$ $[M+H]^+$ 258.1341, found 258.1340.

[(5*R*)-3-(2-Hydroxyethyl)-2-isoxazolin-5-yl]carboxylic acid methyl ester (4)

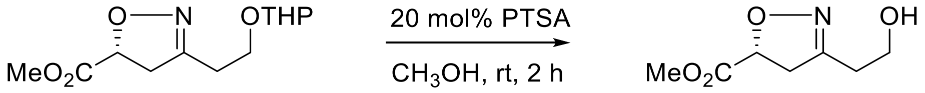

To a solution of **3** (276 mg, 1.07 mmol) in MeOH (8 mL) was added *p*-TsOH·H_2O (37 mg, 0.21 mmol). After stirring for 2 h at room temperature, the mixture was concentrated and purified by column chromatography.

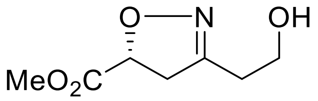

4: Yellow oil (176 mg, 95% yield), ee = 80%, R_f= 0.42 (1:1 hexanes/ AcOEt). $[\alpha]_D^{20}$ = −55.6 (*c*=1.28, $CHCl_3$). ^{1}H NMR (400 MHz, $CDCl_3$) δ: 5.01–4.96 (m, 1H, OC**H**CO), 3.90–3.89 (m, 1H, HOC**H**$_2$CH$_2$), 3.78 (s, 1H, OC**H**$_3$), 3.30–3.27 (m, 2H, NCC**H**$_2$CH), 2.60–2.57 (m, 2H, OHCH$_2$C**H**$_2$); ^{13}C NMR (100 MHz, $CDCl_3$) δ: 170.9, 157.2, 59.2, 52.8, 41.7, 30.6; IR (cm^{-1}): 3373, 2958, 2924, 2850, 1739, 1653, 1558, 1506, 1437, 1261, 1220, 1051, 1028, 873, 800, 501; MS (ESI): calculated for $C_7H_{11}NO_4$ $[M+H]^+$ 174.0766, found 174.0760; HPLC (Daicel AD-H column, *n*-hexane: *i*-PrOH = 80: 20, Flow rate = 1 mL/min, λ = 225 nm.): t_{major} = 7.8 min, t_{minor} = 9.1 min.

(5*R*)-5-Hydroxymethyl-3-[2-(2-tetrahydropyranyloxy)ethyl]-2-isoxazoline (5)

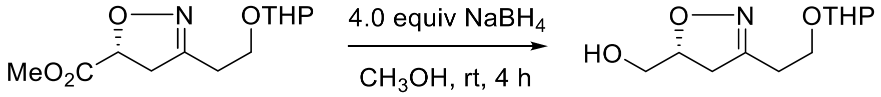

To a solution of **4** (244 mg, 0.95 mmol) in MeOH (10 mL) was added $NaBH_4$ (148 mg, 3.79 mmol). After stirring at room temperature for 4 h, the mixture was purified by column chromatography.

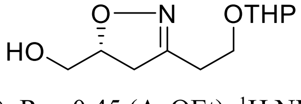

5: Colorless oil (206 mg, 95% yield), R_f= 0.45 (AcOEt). ^{1}H NMR (400 MHz, $CDCl_3$) δ: 4.64–4.63 (m, 1H, OC**H**CH$_2$), 4.58 (m, 1H, OC**H**O), 3.95–3.47 (m, 6H, C**H**$_2$O and C**H**$_2$O and C**H**$_2$O), 3.05–2.98 (m, 1H, CC**H**$_2$CH), 2.91–2.85 (m, 1H, CC**H**$_2$CH), 2.63–2.62 (m, 2H, CH$_2$C**H**$_2$), 2.48 (m, 1H, CH$_2$O**H**), 1.78–1.49 (m, 6H, C**H**$_2$C**H**$_2$C**H**$_2$); ^{13}C NMR (150 MHz, $CDCl_3$) δ: 157.3, 98.6, 79.9, 64.1, 63.2, 62.1, 38.7, 30.3, 27.9, 25.0, 19.2; IR (cm^{-1}): 3419, 2942, 2817, 2739, 2247, 1627, 1418, 1367, 1200, 1135, 1120, 905, 869, 814, 732; MS (ESI): calculated for $C_{11}H_{19}NO_4$ $[M+H]^+$ 230.1392, found

230.1383.

(5*R*)-5-Benzoyloxymethyl-3-[2-(2-tetrahydropyranyloxy)ethyl]-2-isoxazoline (6)

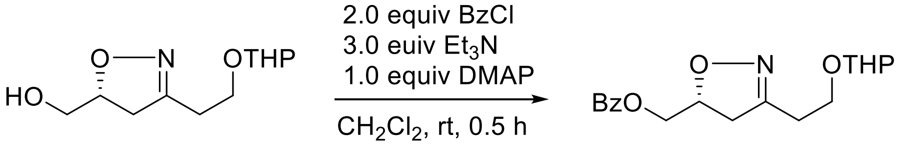

To a solution of **5** (414 mg, 1.81 mmol) in CH_2Cl_2 (20 mL) were added Et_3N (760 µL, 5.43 mmol), DMAP (221 mg, 1.81 mmol) and benzoyl chloride (416 µL, 3.62 mmol). After stirring for 0.5 h at room temperature, the mixture was purified by column chromatography directly.

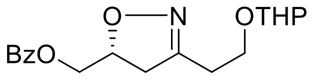

6: Colorless oil (585 mg, 97% yield), $R_f = 0.57$ (1:1 hexanes/AcOEt). ^{1}H NMR (400 MHz, $CDCl_3$) δ: 8.02–8.00 (m, 2H, ArH), 7.56–7.52 (m, 1H, ArH), 7.44–7.39 (m, 2H, ArH), 4.90–4.87 (m, 1H, OC**H**CH_2), 4.57–4.50 (m, 1H, OC**H**O), 4.37–4.35 (m, 2H, BzOCH_2), 3.94–3.44 (m, 4H, THPOC**H$_2$** and C**H$_2$**OCH), 3.20–3.12 (m, 1H, NCC**H$_2$**CHO), 2.94–2.87 (m, 1H, NCC**H$_2$**CHO), 2.72–2.58 (m, 2H, THPOCH_2C**H$_2$**), 1.76–1.48 (m, 6H, $CH_2CH_2CH_2$); ^{13}C NMR (150 MHz, $CDCl_3$) δ: 166.2, 157.1, 157.0, 133.3, 129.9, 129.7, 129.5, 128.8, 128.6, 128.4, 98.9, 77.4, 77.1, 65.5, 64.4, 64.3, 62.5, 62.4, 39.9, 30.6, 29.7, 28.2, 28.1, 25.3, 19.6, 19.5; IR (cm^{-1}): 3429, 3064, 3944, 1788, 1722, 1601, 1451, 1315, 1273, 1120, 1033, 974, 869, 814, 712; MS (ESI): calculated for $C_{18}H_{23}NO_5$ $[M+H]^+$ 334.1654, found 334.1643.

(5*R*)-5-Benzoyloxymethyl-3-(2-hydroxyethyl)-2-isoxazoline (6′)

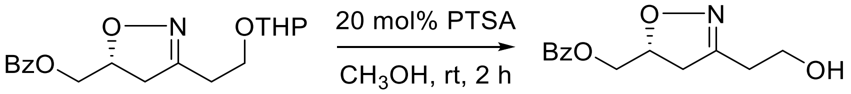

To a solution of **6** (294 mg, 0.88 mmol) in MeOH (4 mL) was added *p*-TsOH·H_2O (30 mg, 0.2 equiv). After stirring for 2 h at room temperature, the mixture was concentrated and purified by column chromatography.

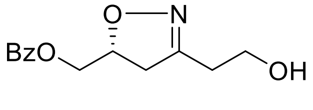

6′: Colorless oil (208 mg, 95% yield), $R_f = 0.24$ (1:1 hexanes/AcOEt). ^{1}H NMR (600 MHz, $CDCl_3$) δ: 7.94–7.92 (m, 2H, ArH), 7.48–7.44 (m, 1H, ArH), 7.35–7.32 (m, 2H, ArH), 4.81–4.80 (m, 1H, OC**H**CH_2), 4.29–4.26 (m, 2H, BzOCH_2), 3.78–3.74 (m, 2H, HOC**H$_2$**), 3.41 (s, 1H, OH), 3.12–3.05 (m, 1H, NCC**H$_2$**CHO), 2.84–2.78 (m, 1H, NCC**H$_2$**CHO), 2.51–2.48 (m, 2H, HOCH_2C**H$_2$**); ^{13}C

NMR (100 MHz, $CDCl_3$) δ: 166.0, 157.1, 132.9, 129.3, 129.2, 128.1, 65.2, 58.7, 39.5, 30.5; IR (cm^{-1}): 3418, 3065, 2955, 2887, 1716, 1600, 1583, 1452, 1315, 1275, 1122, 1070, 871, 713; MS (ESI): calculated for $C_{13}H_{15}NO_4$ $[M+H]^+$ 250.1079, found 250.1076.

(2*R*)-1-Benzoyloxy-2-hydroxy-6-(2-tetrahydropyranyloxy)-4-hexanone (7)

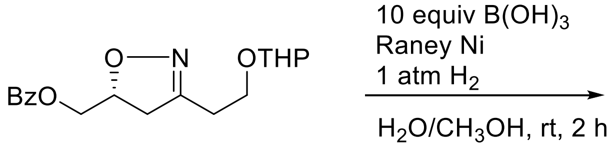

To a solution of **6** (208 mg, 0.62 mmol) in MeOH (30 mL) and H_2O (6 mL) were added $B(OH)_3$ (385 mg, 6.22 mmol) and Raney nickel (250 mg). The reaction mixture was placed under 1 atm of H_2 and well stirred at room temperature for 2 h. The catalyst was filtered off and the filtrate concentrated. The residue was partitioned between sat. aq. $NaHCO_3$ solution (30 mL) and CH_2Cl_2 (50 mL). The organic layer was separated. The aqueous layer was extracted with CH_2Cl_2 (3 × 20 mL). The combined organic layers were dried over Na_2SO_4 and concentrated. The crude product was purified by column chromatography.

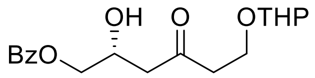

7: Colorless oil (177 mg, 85% yield), R_f = 0.37 (1:1 hexanes/AcOEt). ^{1}H NMR (400 MHz, $CDCl_3$) δ: 8.06–8.04 (m, 2H, ArH), 7.59–7.55 (m 1H, ArH), 7.46–7.42 (m, 2H, ArH), 4.58–4.57 (m, 1H, OC**H**O), 4.48–4.41 (m, 1H, C**H**OH), 4.39–4.35 (m, 2H, $BzOCH_2$), 4.04–3.48 (m, 4H, THPOC**H_2** and C**H_2**OCH), 3.34 (m, 1H, CHO**H**), 2.80–2.73 (m, 4H, $THPOCH_2C$**H_2**COC**H_2**), 1.77–1.50 (m, 6H, $CH_2CH_2CH_2$); ^{13}C NMR (150 MHz, $CDCl_3$) δ: 209.1, 166.6, 133.2, 129.9, 129.7, 128.5, 99.1, 67.8, 66.1, 62.6, 62.5, 62.4, , 46.5, 43.7, 30.6, 29.7, 25.4, 19.6; IR (cm^{-1}): 3459, 2944, 2875, 1717, 1451, 1315, 1275, 1027, 977, 869, 813, 713; MS (ESI): calculated for $C_{18}H_{24}O_6$ $[M+H]^+$ 337.1651, found 337.1649.

(4*R*,6*R*)-6-Benzoyloxymethyl-2-ethyl-4-[2-(2-tetrahydropyranyloxy)ethyl]-1,3,2-dioxaborolane (8)

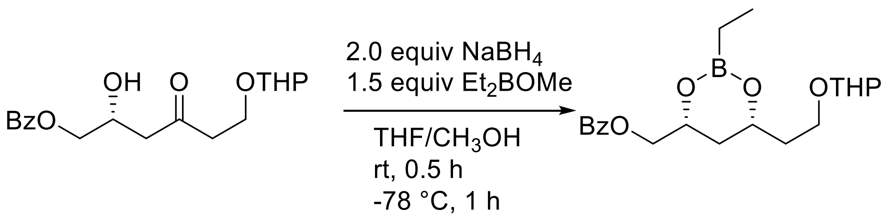

To a solution of **7** (1.73 g, 5.14 mmol) in THF (20 mL) and MeOH (4 mL) was added Et_2BOMe

(7.71 mL, 7.71 mmol, 1 mol/L in THF), and the solution was stirred for 30 min at room temperature before being cooled to −78 °C. $NaBH_4$ (339 mg, 10.28 mmol) was added portionwise over 10 min (moderate gas evolution observed). After the solution was stirred for 1 h at −78 °C, 20 mL of sat. aq. NH_4Cl was added to the cold solution. The mixture was allowed to warm to room temperature and then extracted with EtOAc (50 mL × 3). The combined layers were washed successively with saturated NaCl (20 mL) before being dried over Na_2SO_4 and concentrated in vacuo to give a viscous pale yellow oil. Flash chromatography with hexanes/AcOEt (5:1) as eluent provided the desired product.

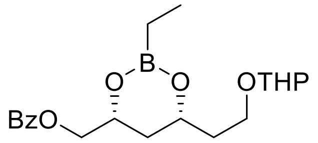

8: Colorless oil (1.86 g, 96% yield), R_f = 0.72 (1:1 hexanes/AcOEt). $^{11}B\{^1H\}$ NMR (128 MHz, $CDCl_3$) δ: 31.46; ^1H NMR (600 MHz, $CDCl_3$) δ: 8.06–8.03 (m, 2H, ArH), 7.58–7.54 (m 1H, ArH), 7.46–7.42 (m, 2H, ArH), 4.60–4.57 (m, 1H, OC**H**O), 4.37–4.31 (m, 3H, C**H**OB and BzOC**H**$_2$), 4.21–4.15 (m, 1H, C**H**OB), 3.99–3.46 (m, 4H, THPOC**H$_2$** and C**H$_2$**OCH), 2.06–1.76 (m, 4H, THPOCH$_2$C**H**$_2$COC**H**$_2$), 1.73–1.44 (m, 6H, CH$_2$CH$_2$CH$_2$), 0.89–0.85 (m, 3H, BCH$_2$**CH$_3$**), 0.71–0.66 (m, 2H, B**CH$_2$**CH$_3$); ^{13}C NMR (150 MHz, $CDCl_3$) δ: 166.3, 133.0, 129.9, 129.6, 128.3, 99.3, 98.6, 69.3, 68.1, 68.0, 67.6, 63.4, 63.1, 62.5, 62.1, 37.4, 37.3, 35.5, 35.4, 30.7, 25.4, 19.7, 19.5, 7.6; IR (cm^{-1}):2951, 2878, 1724, 1452, 1431, 1400, 1335, 1271, 1213, 1118, 1070, 1026, 981, 711; MS (ESI): calculated for $C_{20}H_{29}BO_6$ $[M+Na]^+$ 399.1955, found 399.1957.

(2*R*,4*R*)-1-Benzoyloxy-6-(2-tetrahydropyranyloxy)hexane-2,4-diol (9)

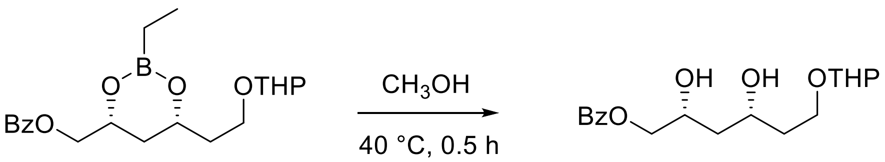

A solution of **8** (222 mg, 0.59 mmol) in MeOH (20 mL) was rotary evaporated at 40 °C for 4 times. The mixture was purified by column chromatography.

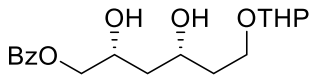

9: Colorless oil (198 mg, 99% yield), R_f = 0.24 (AcOEt). ^1H NMR (600 MHz, $CDCl_3$) δ: 8.06–8.05 (m, 2H, ArH), 7.58–7.54 (m 1H, ArH), 7.45–7.41 (m, 2H, ArH), 4.59–4.55 (m, 1H, OC**H**O), 4.36–4.24 (m, 3H, C**H**OH and BzOC**H**$_2$), 4.20–4.14 (m, 1H, C**H**OH), 4.01–3.49 (m, 5H, THPOC**H$_2$** and

C**H**$_2$OCH and CHO**H**), 1.87–1.72 (m, 4H, THPOCH$_2$C**H**$_2$COC**H**$_2$), 1.61–1.52 (m, 6H, CH$_2$CH$_2$CH$_2$); ^{13}C NMR (150 MHz, CDCl$_3$) δ: 166.6, 133.0, 129.9, 129.7, 128.3, 99.7, 99.2, 72.1, 71.1, 70.4, 70.3, 68.7, 66.1, 65.5, 63.3, 62.6, 39.6, 39.5, 36.9, 36.8, 30.7, 30.5, 25.2, 19.9, 19.6; IR (cm^{-1}): 3435, 2872, 1720, 1452, 1383, 1352, 1315, 1275, 1118, 1072, 1026, 976, 713; MS (ESI): calculated for $C_{18}H_{26}O_6$ $[M+Na]^+$ 361.1627, found 361.1624.

(2*R*,4*R*)-1-Benzoyloxyhexane-2,4,6-triol (10)

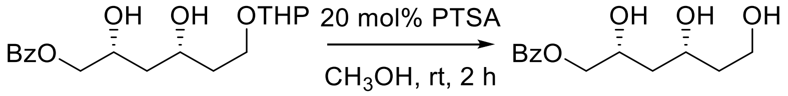

To a solution of **9** (199 mg, 0.59 mmol) in MeOH (8 mL) was added *p*-TsOH·H$_2$O (21 mg, 0.12 mmol). After stirring for 2 h at room temperature, the mixture was concentrated and purified by column chromatography.

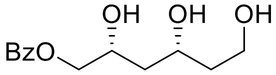

10: Colorless oil (134 mg, 89% yield), $R_f = 0.24$ (AcOEt). ^{1}H NMR (600 MHz, CDCl$_3$) δ: 8.06–8.04 (m, 2H, ArH), 7.59–7.55 (m 1H, ArH), 7.46–7.42 (m, 2H, ArH), 4.37–4.18 (m, 4H, C**H$_2$**OH and BzOC**H$_2$**), 4.07 (s, 1H, CH$_2$O**H**), 3.93–3.79 (m, 3H, C**H**OH and C**H**OH and O**H**), 2.75 (s, 1H, O**H**), 1.80–1.66 (m, 4H, C**H$_2$**CHOHC**H$_2$**CHOH); ^{13}C NMR (150 MHz, CDCl$_3$) δ: 166.8, 133.2, 129.7, 129.6, 128.4, 72.2, 70.8, 68.9, 61.3, 39.4, 38.7, 29.7; IR (cm^{-1}): 3375, 2918, 1716, 1452, 1279, 1116, 1099, 1070, 711; MS (ESI): calculated for $C_{13}H_{18}O_5$ $[M+Na]^+$ 277.1052, found 277.1048.

(2*R*,4*R*)-1-Benzoyloxy-6-(*tert*-butyldimethylsilyloxy)hexane-2,4-diol (11)

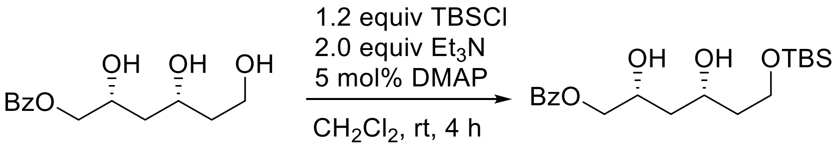

To a stirred solution of **10** (117 mg, 0.46 mmol), Et$_3$N (129 μL, 0.92 mmol) and DMAP (3 mg, 0.023 mmol) in CH$_2$Cl$_2$ (4 mL) was added TBS-Cl (83 mg, 0.552 mmol). After stirring for 4 h at room temperature, the mixture was poured into sat. aq. NH$_4$Cl (25 mL). The organic layer was separated, and the aqueous portion extracted with CH$_2$Cl$_2$ (3 × 10 mL). The combined organic extracts were washed with sat. aq. NaCl (20 mL) and dried. Removal of the solvent in vacuo followed by column chromatography of the residue afforded pure **11**.

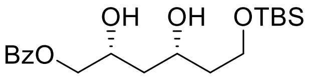

11: Colorless oil (147 mg, 87% yield), $R_f = 0.47$ (1:1 hexanes/AcOEt). ^{1}H NMR (400 MHz, $CDCl_3$) δ: 8.07–8.05 (m, 2H, ArH), 7.58–7.54 (m 1H, ArH), 7.45–7.41 (m, 2H, ArH), 4.32–4.17 (m, 5H, C**H₂**OTBS and OH and BzOC**H₂**), 4.05 (s, 1H, CHO**H**), 3.94–3.81 (m, 2H, C**H**OH and C**H**OH), 1.77–1.66 (m, 4H, C**H₂**CHOHC**H₂**CHOH), 0.89 (s, 9H, C(C**H₃**)₃), 0.08 (s, 6H, Si(C**H₃**)₂); ^{13}C NMR (100 MHz, $CDCl_3$) δ: 166.6, 133.0, 130.0, 129.7, 128.3, 72.9, 70.3, 68.7, 62.6, 25.8, 18.1, −5.6, −5.6; IR (cm^{-1}): 3416, 2953, 2829, 2856, 1722, 1452, 1275, 1257, 1097, 1072, 837, 777, 711; MS (ESI): calculated for $C_{19}H_{32}O_5Si$ $[M+Na]^+$ 391.1917, found 391.1913.

(4*R*,6*R*)-6-Benzoyloxymethyl-4-[2-(*tert*-butyldimethylsilyloxy)ethyl]-2,2-dimethyl-1,3-dioxane (12)

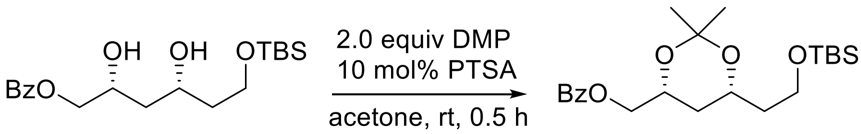

To a solution of **11** (111 mg, 0.30 mmol) in acetone (4 mL) were added 2,2-dimethoxpropane (63 mg, 0.60 mmol) and PTSA (5 mg, 0.03mmol). The resulting mixture was stirred at room temperature until TLC analysis indicated consumption of the starting material (0.5 h). Et_3N (3 mL) was added, and the mixture concentrated and purified by column chromatography.

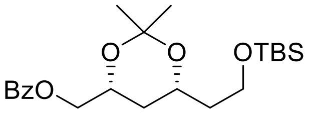

12: Colorless oil (121 mg, 99% yield), $R_f = 0.66$ (3:1 hexanes/AcOEt). ^{1}H NMR (400 MHz, $CDCl_3$) δ: 8.06-8.03 (m, 2H, ArH), 7.55–7.53 (m 1H, ArH), 7.45–7.41 (m, 2H, ArH), 4.30–4.09 (m, 4H, C**H₂**OTBS and BzOC**H₂**), 3.75–3.65 (m, 2H, C**H**OC(CH₃)₂ and C**H**OC(CH₃)₂), 1.69–1.58 (m, 3H, C**H₂**CHOC**H**₂CHO), 1.58 (s, 3H, C(C**H₃**)₂), 1.47 (s, 3H, C(C**H₃**)₂), 1.42–1.33 (m, 1H, one of CH₂CHOCC**H₂**CHOC), 0.89 (s, 9H, C(C**H₃**)₃), 0.04 (s, 6H, Si(C**H₃**)₂); ^{13}C NMR (100 MHz, $CDCl_3$) δ: 166.3, 132.9, 130.0, 129.6, 128.3, 98.7, 67.5, 67.4, 65.2, 58.6, 39.4, 33.6, 30.0, 25.9, 19.7, 18.2, −5.4; IR (cm^{-1}): 2953, 2928, 1724, 1471, 1379, 1273, 1170, 1105, 1099, 835, 775, 711; MS (ESI): calculated for $C_{22}H_{36}O_5Si$ $[M+Na]^+$ 431.2230, found 431.2222.

(4*R*,6*R*)-6-Benzoyloxymethyl-2,2-dimethyl-4-(2-hydroxyethyl)-1,3-dioxane (13)

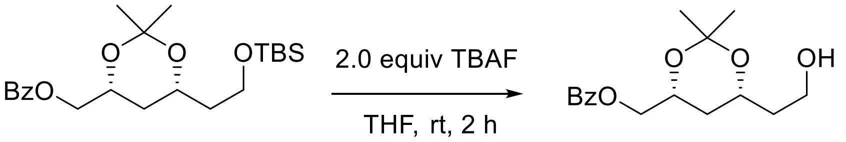

To a stirred solution of **12** (119 mg, 0.29 mmol) in THF (4 mL) was added 1 M tetrabutylammonium fluoride in THF (0.58 mL, 0.58 mmol). The mixture was stirred for 2 h at room temperature. The reaction was quenched with aqueous NH_4Cl. The layers were separated, and the aqueous layer extracted with AcOEt. The combined organic layers were washed with brine and dried over Na_2SO_4. After evaporation of the solvent under reduced pressure, the residue was purified by column chromatography.

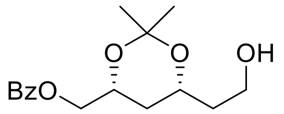

13: Colorless oil (84 mg, 99% yield), $R_f = 0.41$ (1:1 hexanes/AcOEt). ^{1}H NMR (400 MHz, $CDCl_3$) δ: 8.05–8.03 (m, 2H, ArH), 7.57–7.54 (m 1H, ArH), 7.45–7.41 (m, 2H, ArH), 4.29–4.15 (m, 4H, C**H**$_2$OH and BzOC**H$_2$**), 3.78–3.75 (m, 2H, C**H**OC(CH_3)$_2$ and C**H**OC(CH_3)$_2$), 2.56 (s, 1H, OH), 1.78–1.72 (m, 2H, C**H$_2$**CHOC**H**$_2$CHO), 1.58–1.55 (m, 1H, one of C**H$_2$**CHOC**H**$_2$CHO), 1.48 (s, 3H, C(C**H$_3$**)$_2$), 1.47–1.43 (m, 1H, one of C**H$_2$**CHOC**H**$_2$CHO), 1.42 (s, 3H, C(C**H$_3$**)$_2$); ^{13}C NMR (100 MHz, $CDCl_3$) δ: 166.4, 133.0, 129.9, 129.6, 128.3, 98.87, 68.5, 67.3, 62.3, 60.4, 38.2, 33.0, 29.9, 29.6, 19.7; IR (cm^{-1}): 3487, 2920, 1720, 1452, 1381, 1276, 1201, 1168, 1114, 912, 732, 713, 648; MS (ESI): calculated for $C_{16}H_{22}O_5$ $[M+Na]^+$ 317.1365, found 317.1358.

[(4*S*,6*R*)-6-Benzoyloxymethyl-2,2-dimethyl-1,3-dioxan-4-yl]acetic acid (14)

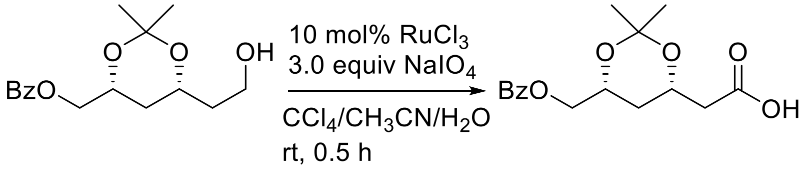

To a stirred solution of **13** (152 mg, 0.52 mmol) and $NaIO_4$ (334 mg, 1.56 mmol) in CCl_4 (2 mL), CH_3CN (2 mL), and H_2O (1.2 mL) was added ruthenium trichloride hydrate (12 mg, 0.052 mmol), and the mixture stirred for 30 min at room temperature. After this period, CH_2Cl_2 (25 mL) was added, and the layers were separated. The aqueous layer was extracted with CH_2Cl_2. The combined organic layers were dried over Na_2SO_4. The mixture was concentrated and purified by column chromatography.

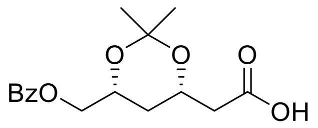

14: Colorless oil (138 mg, 86% yield), R_f = 0.40 (1:1 hexanes/AcOEt). ^{1}H NMR (400 MHz, $CDCl_3$) δ: 8.05–8.03 (m, 2H, ArH), 7.57–7.53 (m 1H, ArH), 7.45–7.41 (m, 2H, ArH), 4.39–4.25 (m, 4H, C**H**OC(CH_3)$_2$ and C**H**OC(CH_3)$_2$ and BzOC**H$_2$**), 2.63–2.45 (m, 2H, C**H**$_2$COOH), 1.71–1.68 (m, 1H, OCHC**H**$_2$CHO), 1.48 (s, 3H, C(C**H$_3$**)$_2$), 1.43–1.38 (m, 1H, OCHC**H**$_2$CHO), 1.41 (s, 3H, C(C**H$_3$**)$_2$); ^{13}C NMR (100 MHz, $CDCl_3$) δ: 176.2, 166.4, 133.0, 129.8, 129.6, 128.3, 99.2, 67.2, 67.1, 65.3, 41.0, 32.6, 29.7, 19.5; IR (cm^{-1}): 3200, 2993, 2945, 1716, 1383, 1276, 1201, 1168, 1118, 995, 958, 947, 713; MS (ESI): calculated for $C_{16}H_{20}O_6$ [M+Na]$^+$ 331.1158, found 331.1158.

[(4*S*,6*R*)-6-Benzoyloxymethyl-2,2-dimethyl-1,3-dioxan-4-yl]acetic acid *t*-butyl ester (15)

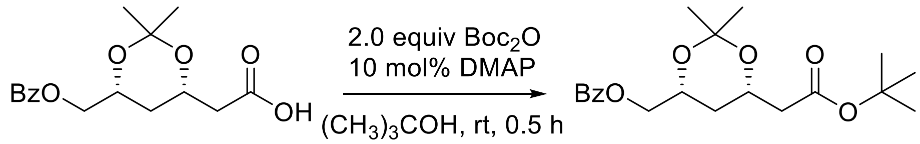

To a stirred solution of **14** (115 mg, 0.37 mmol) in *tert*-butyl alcohol (4 mL) were added BOC anhydride (162 mg, 0.74 mmol), DMAP (5 mg, 0.037 mmol), and the reaction mixture was stirred for 0.5 h at room temperature. The reaction was quenched with aqueous NH_4Cl. The layers were separated, and the aqueous layer was extracted with AcOEt. The combined organic layers were washed with brine and dried over Na_2SO_4. After evaporation of the solvent under reduced pressure, the residue was purified by column chromatography.

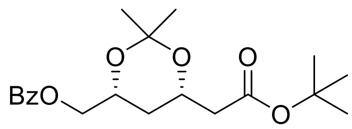

15: Colorless oil (116 mg, 86% yield), ee = 74%, R_f = 0.37 (5:1 hexanes/AcOEt). $[\alpha]_D^{20}$ = −4.5 (*c*=0.88, $CHCl_3$). ^{1}H NMR (400 MHz, $CDCl_3$) δ: 8.04–8.02 (m, 2H, ArH), 7.56–7.52 (m 1H, ArH), 7.44–7.40 (m, 2H, ArH), 4.32–4.23 (m, 4H, C**H**OC(CH_3)$_2$ and C**H**OC(CH_3)$_2$ and BzOC**H$_2$**), 2.47–2.30 (m, 2H, C**H**$_2$COOC(CH_3)$_3$), 1.67–1.63 (m, 1H, one of OCHC**H**$_2$CHO), 1.47 (s, 3H, C(C**H$_3$**)$_2$), 1.42 (s, 9H, C(CH_3)$_3$), 1.39 (s, 3H, C(C**H$_3$**)$_2$), 1.37–1.31 (m, 1H, one of OCHC**H**$_2$CHO); ^{13}C NMR (100 MHz, $CDCl_3$) δ: 169.9, 166.3, 132.9, 129.3, 129.6, 128.3, 98.9, 80.6, 67.3, 67.2, 65.8, 42.6, 32.8, 29.9, 27.9, 19.6; IR (cm^{-1}): 2980, 2941, 1724, 1602, 1452, 1381, 1315, 1275, 1153, 1114, 1070, 995, 844, 713; MS (ESI): calculated for $C_{20}H_{28}O_6$ [M+Na]$^+$ 387.1784, found 387.1782; HPLC

(Daicel OD-H column, *n*-hexane: *i*-PrOH = 90: 10, Flow rate = 0.5 mL/min, λ = 254 nm.): t_{major} = 13.7 min, t_{minor} = 10.9 min.

[(4*S*,6*R*)-2,2-Dimethyl-6-hydroxymethyl-1,3-dioxan-4-yl]acetic acid *t*-butyl ester (16)

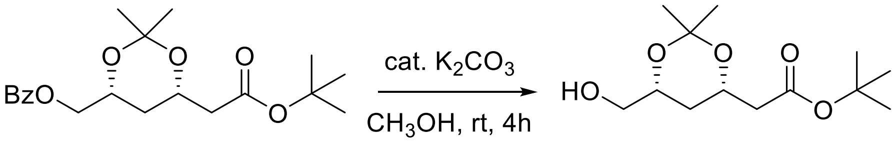

A mixture of **15** (98 mg, 0.25 mmol), solid K_2CO_3 (13 mg, 0.125 mmol) and MeOH (8 mL) was stirred at room temperature for 4 h. It was concentrated and purified by column chromatography.

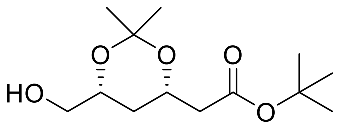

16: Colorless oil (57 mg, 87% yield), R_f = 0.42 (1:1 hexanes/AcOEt). ^{1}H NMR (400 MHz, $CDCl_3$) δ: 4.29–4.00 (m, 1H, one of C**H**$_2$OH), 3.99–3.96 (m, 1H, one of C**H**$_2$OH), 3.59–3.45 (m, 2H, C**H**OC$(CH_3)_2$ and C**H**OC$(CH_3)_2$), 2.44–2.26 (m, 3H, C**H**$_2$COOC$(CH_3)_3$ and CH_2O**H**), 1.48–1.28 (m, 2H, OCHC**H**$_2$CHO), 1.44 (s, 3H, C(C**H**$_3$)$_2$), 1.42 (s, 9H, C$(CH_3)_3$), 1.36 (s, 3H, C(C**H**$_3$)$_2$); ^{13}C NMR (100 MHz, $CDCl_3$) δ: 170.1, 98.8, 80.6, 69.5, 65.8, 65.7, 42.6, 31.8, 29.9, 28.0, 19.7; IR (cm^{-1}): 3449, 2980, 2937, 1730, 1369, 1315, 1259, 1201, 1153, 1078, 1024, 950, 842; MS (ESI): calculated for $C_{13}H_{24}O_5$ [M+Na]$^+$ 283.1521, found 283.1516.

[(4*S*,6*R*)-6-Acetoxymethyl-2,2-dimethyl-1,3-dioxan-4-yl]acetic acid *t*-butyl ester (17)

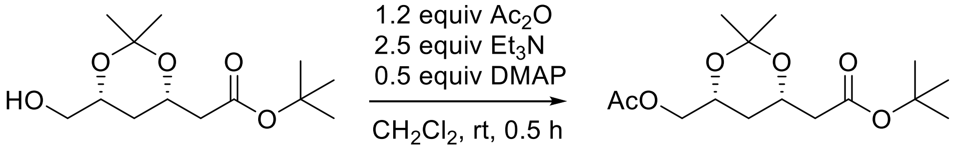

To a solution of **16** (47 mg, 0.18 mmol) in CH_2Cl_2 (5 mL) were added Et_3N (63 μL, 0.45 mmol), DMAP (11 mg, 0.09 mmol) and Ac_2O (23 mg, 0.22 mmol). After stirring for 0.5 h at room temperature, the mixture was purified by column chromatography directly.

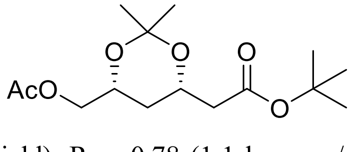

17: Colorless oil (54 mg, 99% yield), R_f = 0.78 (1:1 hexanes/AcOEt). $[\alpha]_D^{20}$ = −11.67 (*c*=0.54, $CHCl_3$). ^{1}H NMR (400 MHz, $CDCl_3$) δ: 4.30–3.97 (m, 4H, AcOC**H**$_2$ and C**H**OC$(CH_3)_2$ and C**H**OC$(CH_3)_2$), 2.46–2.83 (m, 2H, C**H**$_2$COOC$(CH_3)_3$), 2.07 (s, 3H, OCOC**H**$_3$), 1.58–1.54 (m, 1H, one of OCHC**H**$_2$CHO), 1.45 (s, 3H, C(C**H**$_3$)$_2$), 1.44 (s, 9H, C(C**H**$_3$)$_3$), 1.38 (s, 3H, C(C**H**$_3$)$_2$), 1.29–

1.21 (m, 1H, one of OCHC**H**$_2$CHO); ^{13}C NMR (100 MHz, CDCl$_3$) δ: 170.9, 170.1, 98.9, 80.7, 67.2, 67.0, 65.8, 42.6, 32.5, 29.9, 28.1, 20.9, 19.6; IR (cm^{-1}): 2980, 1739, 1367, 1238, 1155, 1035, 864; MS (ESI): calculated for $C_{15}H_{26}O_6$ $[M+Na]^+$ 325.1627, found 325.1619.

(4*R*,6*R*)-6-Benzoyloxymethyl-2,2-dimethyl-4-[2-(2-tetrahydropyranyloxy)ethyl]-1,3-dioxane (18)

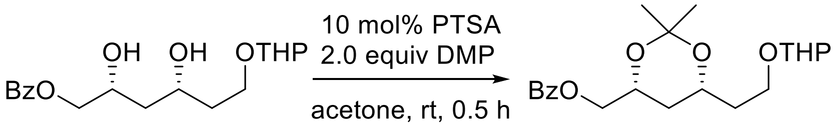

To a solution of **9** (113 mg, 0.33 mmol) in acetone (4 mL) were added 2,2-dimethoxpropane (69 mg, 0.66 mmol) and PTSA (6 mg, 0.033 mmol). The resulting mixture was stirred at room temperature until TLC analysis indicated consumption of the starting material (0.5 h). Et_3N (3 mL) was added, and the mixture concentrated and purified by column chromatography.

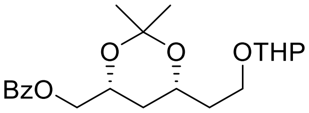

18: Colorless oil (123 mg, 99% yield), $R_f = 0.77$ (1:1 hexanes/AcOEt). ^{1}H NMR (600 MHz, CDCl$_3$) δ: 8.05–8.04 (m, 2H, ArH), 7.56–7.54 (m 1H, ArH), 7.44–7.42 (m, 2H, ArH), 4.59–4.55 (m, 1H, OC**H**O), 4.31–4.25 (m, 3H, C**H**OC(CH$_3$)$_2$ and BzOC**H$_2$**), 4.09–4.07 (m, 1H, C**H**OC(CH$_3$)$_2$), 3.86–3.43 (m, 4H, THPOC**H$_2$** and C**H$_2$**OCH), 1.82–1.46 (m, 10H, THPOCH$_2$C**H**$_2$COC**H**$_2$ and C**H**$_2$C**H**$_2$C**H**$_2$); ^{13}C NMR (150 MHz, CDCl$_3$) δ: 166.4, 132.9, 130.1, 129.6, 128.3, 99.3, 98.8, 98.5, 99.3, 98.8, 98.5, 67.5, 67.4, 66.7, 65.5, 63.2, 61.9, 36.5, 36.4, 33.6, 33.4, 30.7, 30.6, 30.0, 29.6, 25.4, 19.7, 19.4; IR (cm^{-1}): 2941, 2370, 1722, 1381, 1274, 1201, 1118, 1026, 867, 711, 412; MS (ESI): calculated for $C_{21}H_{30}O_6$ $[M+Na]^+$ 401.1940, found 401.1937.

(2*R*,4*R*)-1-Benzoyloxyhexane-2,4,6-triol (10)

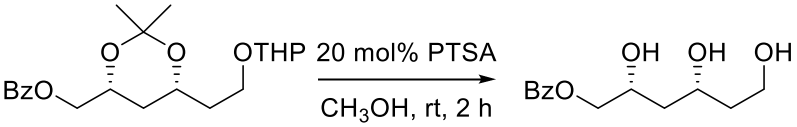

To a solution of **18** (223 mg, 0.59 mmol) in MeOH (4 mL) was added *p*-TsOH·H_2O (21 mg, 0.12 mmol). After stirring for 2 h at room temperature, the mixture was concentrated and purified by column chromatography.

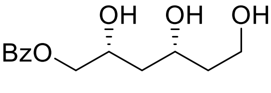

10: Colorless oil (134 mg, 89% yield), R_f = 0.24 (AcOEt).

(2*R*)-1-Benzoyloxy-3-[(4*R*)-2,2-dimethyl-1,3-dioxan-4-yl)]-2-propanol (19)

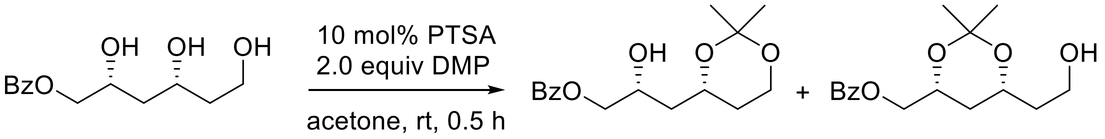

19, 54% yield **13**, 30% yield

To a solution of **10** (168 mg, 0.66 mmol) in acetone (4 mL) were added 2,2-dimethoxpropane (138 mg, 1.32 mmol) and PTSA (11 mg, 0.066 mmol). The resulting mixture was stirred at room temperature until TLC analysis indicated consumption of the starting material (0.5 h). Et_3N (3 mL) was added, and the mixture concentrated and purified by column chromatography.

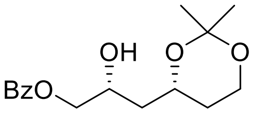

19: Colorless oil (106 mg, 54% yield), R_f = 0.44 (1:1 hexanes/AcOEt). ^1H NMR (600 MHz, $CDCl_3$) δ: 8.03–8.02 (m, 2H, ArH), 7.55–7.54 (m 1H, ArH), 7.43–7.41 (m, 2H, ArH), 4.29–4.23 (m, 3H, C**H**OH and BzOC$\mathbf{H_2}$), 4.16–4.12 (m, 1H, C**H**OC$(CH_3)_2$), 3.81–3.70 (m, 2H, C$\mathbf{H_2}$OC$(CH_3)_2$), 2.57 (s, 1H, OH), 1.78–1.41 (m, 4H, OC$\mathbf{H_2}$CH_2CHOC$\mathbf{H_2}$), 1.47 (s, 3H, C(C$\mathbf{H_3}$)$_2$), 1.41 (s, 3H, C(C$\mathbf{H_3}$)$_2$); MS (ESI): calculated for $C_{16}H_{22}O_5$ $[M+Na]^+$ 317.1365, found 317.1358.

(2*R*,4*R*)-1-Benzoyloxy-2,4-diacetoxy-6-(2-tetrahydropyranyloxy)hexane (20)

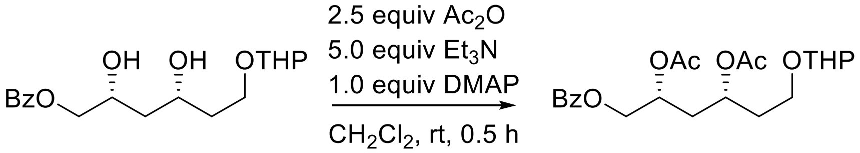

To a solution of **9** (100 mg, 0.30 mmol) in CH_2Cl_2 (5 mL) were added Et_3N (210 μL, 1.5 mmol), DMAP (37 mg, 0.3 mmol) and Ac_2O (77 mg, 0.75 mmol). After stirring for 0.5 h at room temperature, the mixture was purified by column chromatography directly.

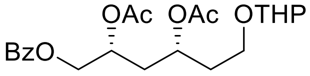

20: Colorless oil (125 mg, 99% yield), R_f = 0.81 (1:1 hexanes/AcOEt). ^1H NMR (600 MHz, $CDCl_3$) δ: 8.01–7.99 (m, 2H, ArH), 7.56–7.53 (m 1H, ArH), 7.44–7.40 (m, 2H, ArH), 5.32–5.12 (m, 2H, two C**H**OCOCH_3), 4.54–4.46 (m, 2H, OC**H**O and one of BzOC$\mathbf{H_2}$), 4.33–4.28 (m, 1H, one of BzOC$\mathbf{H_2}$), 3.83–3.73 (m, 2H, THPOC$\mathbf{H_2}$), 3.47–3.33 (m, 2H, C$\mathbf{H_2}$OCH), 2.07–2.03 (m, 8H, two of THPOCH_2C$\mathbf{H_2}$COC$\mathbf{H_2}$ and two COC$\mathbf{H_3}$), 1.92–1.86 (m, 2H, two of THPOCH_2C$\mathbf{H_2}$COC$\mathbf{H_2}$), 1.79–

1.45 (m, 6H, C**H**$_2$C**H**$_2$C**H**$_2$); ^{13}C NMR (150 MHz, $CDCl_3$) δ: 170.4, 170.2, 166.0, 133.1, 129.7, 128.4, 99.1, 98.5, 68.7, 68.6, 68.5, 65.2, 63.4, 63.3, 62.3, 61.9, 35.1, 33.9, 33.8, 30.5, 29.6, 25.3, 21.1, 20.9, 19.5, 19.2; IR (cm^{-1}): 2943, 2872, 2850, 1739, 1452, 1373, 1276, 1236, 1120, 1070, 1026, 906, 732, 713; MS (ESI): calculated for $C_{22}H_{30}O_8$ $[M+Na]^+$ 445.1838, found 445.1835.

(2*R*,4*R*)-6-Acetoxy-1-benzoyloxyhexane-2,4-diol (21)

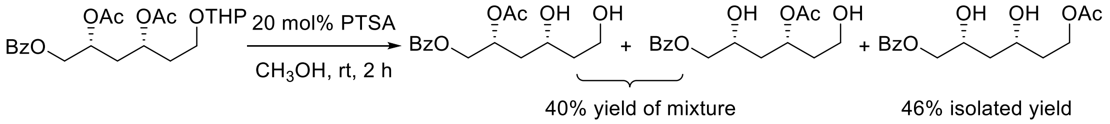

To a solution of **20** (196 mg, 0.46 mmol) in MeOH (4 mL) was added *p*-TsOH·H_2O (16 mg, 0.09 mmol). After stirring for 2 h at room temperature, the mixture was concentrated and purified by column chromatography.

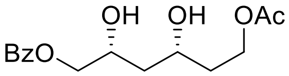

21: Colorless oil (62 mg, 46% yield), $R_f = 0.37$ (1:1 hexanes/AcOEt). ^{1}H NMR (600 MHz, $CDCl_3$) δ: 8.05–8.02(m, 2H, ArH), 7.57–7.55 (m 1H, ArH), 7.45–7.42 (m, 2H, ArH), 4.37–4.23 (m, 4H, C**H**$_2$OAc and BzOC**H**$_2$), 4.14–4.10 (m, 1H, C**H**OH), 4.00–3.96 (m, 1H, C**H**OH), 3.77 (s, 1H, O**H**), 3.68 (s, 1H, O**H**), 2.05 (s, 3H, C**H**$_3$CO), 1.85–1.69 (m, 4H, C**H**$_2$CHOHC**H**$_2$CHOH); MS (ESI): calculated for $C_{15}H_{20}O_6$ $[M+Na]^+$ 319.1158, found 319.1153.

(2*R*,4*S*)-1-Benzoyloxyhexane-2,4,6-triol

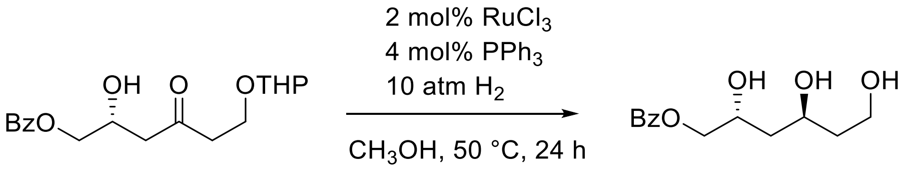

7 (117.6 mg, 0.35 mmol) in degassed methanol (5 mL) was cannulated to a Schlenk tube containing a degassed mixture of $RuCl_3$ (1.5 mg, 0.007 mmol) and PPh_3 (3.6 mg, 0. 014 mmol). The Schlenk tube was placed in a glove box and the reaction mixture transferred to a stainless steel autoclave, which was purged with hydrogen and pressurized to 10 bar. The autoclave was heated to 50 °C. After stirring for 24 h, the autoclave was cooled to room temperature, hydrogen was vented, and the mixture was concentrated and purified by column chromatography.

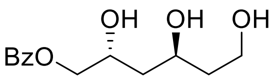

Colorless oil (72 mg, 81% yield), $R_f = 0.24$ (AcOEt). ^{1}H NMR (400 MHz, $CDCl_3$) δ: 8.04–8.03 (m,

2H, ArH), 7.59–7.55 (m 1H, ArH), 7.46–7.42 (m, 2H, ArH), 4.36–4.19 (m, 4H, C**H**$_2$OH and BzOC**H**$_2$), 4.08 (s, 1H, CH$_2$O**H**), 3.91–3.81 (m, 2H, C**H**OH and C**H**OH), 2.78 (s, 1H, O**H**), 1.78–1.66 (m, 4H, C**H**$_2$CHOHC**H**$_2$CHOH); MS (ESI): calculated for $C_{13}H_{18}O_5$ $[M+Na]^+$ 277.1052, found 277.1048.

2. Preparation of the racemic samples of compounds 4 and 15

Preparation of the racemic sample of compound 4

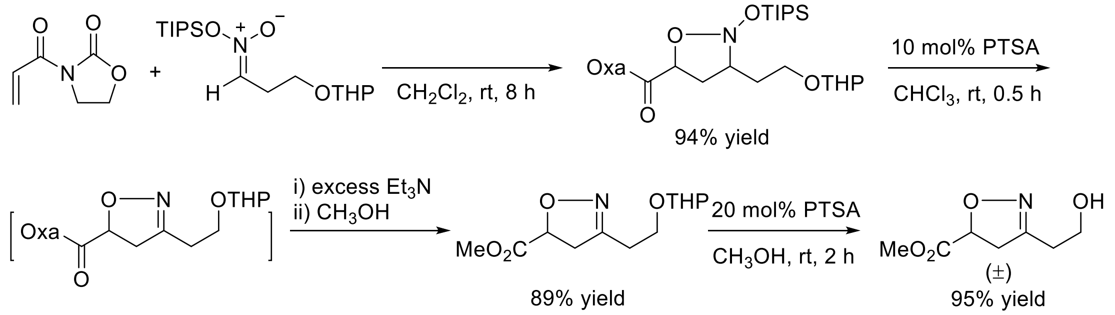

***N*-{(3*S**,5*R**)-[3-(2-(2-Tetrahydropyranyloxy)ethyl)-2-triisopropylsilyloxy-5-isoxazolidinyl]carbonyl}-1,3-oxazolidin-2-one**

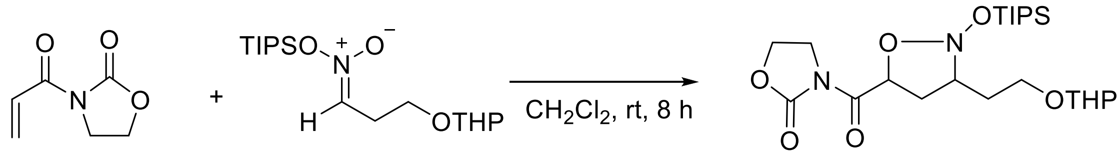

To a dry Schlenk tube were added *N*-acryloyl-1,3-oxazolidin-2-one (282 mg, 2 mmol) and the silyl nitronate (3.0 mmol) in anhydrous CH_2Cl_2 (3 mL). The mixture was stirred at room temperature overnight and monitored by TLC. After the reaction was completed, the product was purified by silica gel chromatography.

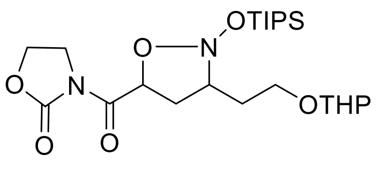

Yellow oil (914 mg, 94 % yield), R_f = 0.40 (1:1 hexanes/AcOEt). ^{1}H NMR (600 MHz, $CDCl_3$) δ: 5.81–5.74 (m, 1H, CH$_2$C**H**O), 4.56-4.55 (m, 1H, OC**H**O), 4.50-4.42 (m, 2H, C**H**$_2$O), 4.14–3.99 (m, 2H, C**H**$_2$O), 3.85–3.77 (m, 2H, OC**H**$_2$CH$_2$), 3.54–3.40 (m, 3H, NC**H** and NC**H**$_2$), 2.77–2.69 (m, 1H, CHC**H**$_2$CH), 2.35–2.29 (m, 1H, CHC**H**$_2$CH), 2.18–2.14 (m, 1H, CH$_2$CH$_2$), 1.89–1.49 (m, 7H, CH$_2$C**H**$_2$ and C**H**$_2$C**H**$_2$C**H**$_2$), 1.27–1.16 (m, 3H, SiCH), 1.10–1.05 (m, 18H, SiCH(C**H**$_3$)$_2$); MS (ESI):

calculated for $C_{23}H_{42}N_2O_7Si$ [M+Na]$^+$ 509.2659, found 509.2659.

{(5*R)-[3-(2-(2-Tetrahydropyranyloxy)ethyl)]-2-isoxazolin-5-yl}carboxylic acid methyl ester**

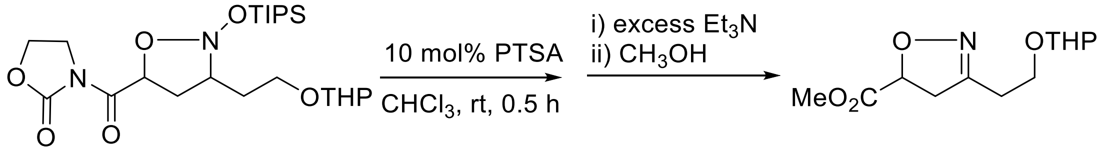

To a solution of the starting isoxazolidine (0.86 g, 1.78 mmol) in $CHCl_3$ (15 mL) was added PTSA (31 mg, 0.178 mmol) at 0 °C. The mixture was allowed to warm to room temperature and stirred until complete consumption of the starting material (0.5 h). Vacuum was applied to remove the solvent before Et_3N (5 mL) was added. After stirring for 5 min, methanol (30 mL) was added and the mixture stirred overnight at room temperature. The crude product was purified by column chromatography.

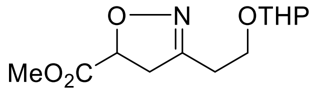

Yellow oil (0.41 g, 89% yield), R_f= 0.42 (1:1 hexanes/ AcOEt).

(±)-[3-(2-Hydroxyethyl)-2-isoxazolin-5-yl]carboxylic acid methyl ester

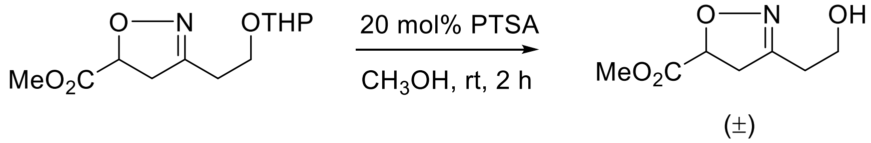

To a solution of the starting 2-isoxazoline (276 mg, 1.07 mmol) in MeOH (8 mL) was added *p*-TsOH·H_2O (37 mg, 0.21 mmol). After stirring for 2 h at room temperature, the mixture was concentrated and purified by column chromatography.

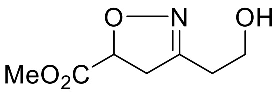

Yellow oil (176 mg, 95% yield), R_f= 0.42 (1:1 hexanes/ AcOEt).

Preparation of the racemic sample of compound 15

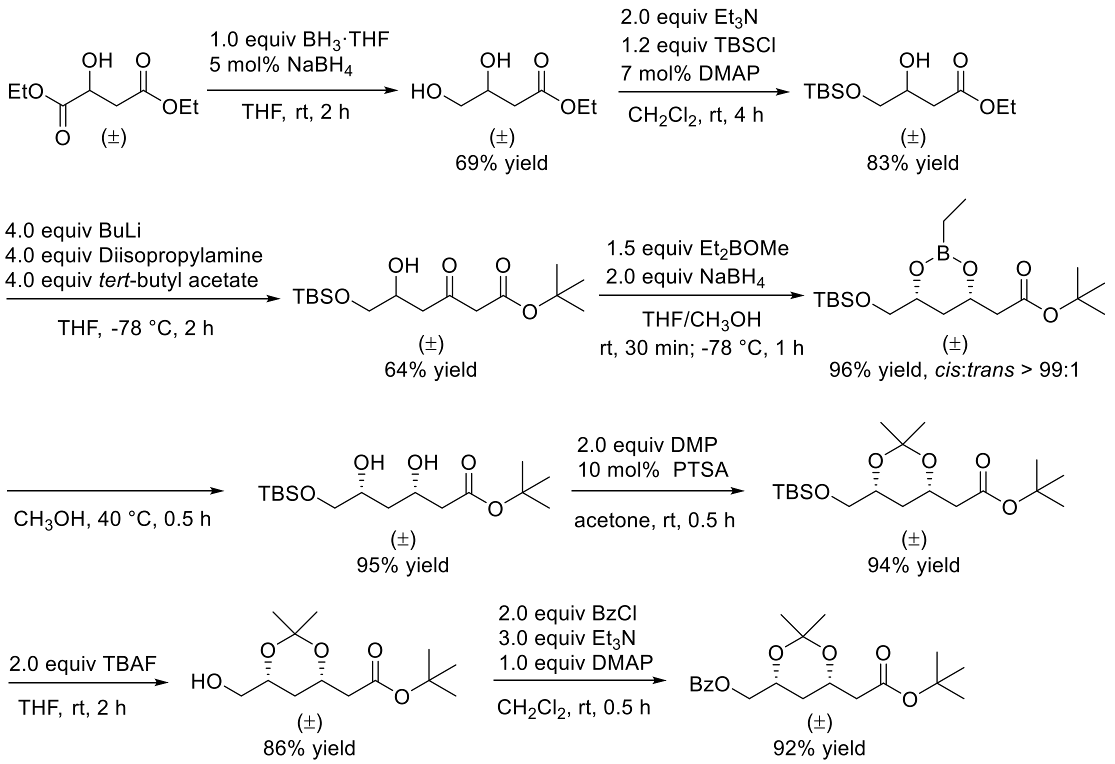

(±)-3,4-Dihydroxybutanoic acid ethyl ester

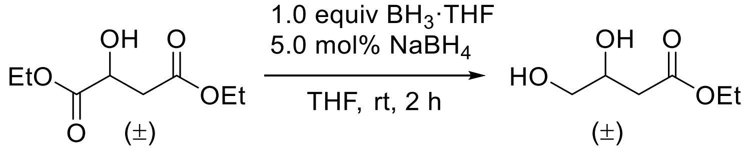

To a solution of diethyl DL-malate (2 g, 10.5 mmol) in THF (10 mL) was added dropwise BH_3 in THF (10.6 mL, 10.6 mmol) over 30 min at 0 °C under N_2. $NaBH_4$ powder (20 mg, 0.525 mmol) was added in one portion (exothermic) under vigorous stirring at that temperature until evolution of hydrogen ceased (30 min). Then, the mixture was allowed to warm to room temperature and stirred until TLC analysis indicated consumption of the starting material (2 h). To the reaction mixture were added MeOH (10 mL) and PTSA (0.525 mg, 5 mol %) and the resulting slightly cloudy solution was stirred for 30 min at room temperature, followed by concentration to give a colorless gum. This was dissolved in MeOH (10 mL) and the resulting solution was concentrated via rotary evaporation again. This operation was repeated. To the residue was added benzene (10 mL) and the solution concentrated via rotary evaporation. This was repeated to eliminate MeOH and $B(OMe)_3$ as thoroughly as possible to give a clear, colorless gum.

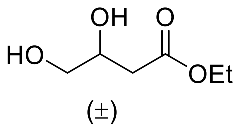

Colorless oil (1.07 g, 69% yield), $R_f = 0.42$ (AcOEt). ^{1}H NMR (400 MHz, $CDCl_3$) δ: 4.20–4.19 (m, 1H, $CH_2O\mathbf{H}$), 4.06–3.94 (m, 4H, C**H**OH and $CH_3C\mathbf{H_2}O$ and O**H**), 3.53–3.35 (m, 2H, C**H₂**OH), 2.38–2.36 (m, 2H, $CHOHC\mathbf{H_2}CO$), 1.14 (t, $J = 7.1$ Hz, 3H, $OCH_2C\mathbf{H_3}$).

(±)-4-(*tert*-Butyldimethylsilyloxy)-3-hydroxybutanoic acid ethyl ester

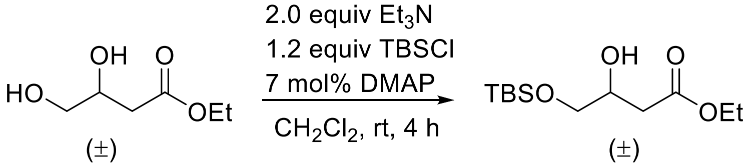

To a stirred solution of the starting dihydroxy ester (2.79 g, 18 mmol), Et_3N (5 mL, 0.36 mmol) and DMAP (146.4 mg, 1.21 mmol) in CH_2Cl_2 (20 mL) was added TBS-Cl (3.2 g, 21.6 mmol). After stirring for 4 h at room temperature, the mixture was poured into sat. aq. NH_4Cl (25 mL), the organic layer was separated, and the aqueous portion was extracted with CH_2Cl_2 (3 × 10 mL). The combined organic extracts were washed with saturated NaCl (20 mL) and dried. Removal of the solvent in vacuo followed by column chromatography of the residue afforded pure product.

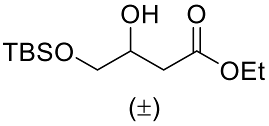

Colorless thick liquid (3.9 g, 83% yield), $R_f = 0.43$ (5:1 hexanes/ AcOEt). ^{1}H NMR (600 MHz, $CDCl_3$) δ: 4.09–4.05 (m, 2H, $OC\mathbf{H_2}CH_3$), 3.99–3.98 (m, 1H, C**H**OH), 3.52–3.51 (m, 2H, $C\mathbf{H_2}OTBS$), 3.02–3.01 (m, 1H, CHOH), 2.46–2.39 (m, 2H, $CHOHC\mathbf{H_2}CO$), 1.19–1.17 (m, 3H, $OCH_2C\mathbf{H_3}$), 0.81 (s, 9H, $C(C\mathbf{H_3})_3$), 0.01 (s, 6H, $Si(C\mathbf{H_3})_2$).

(±)-6-(*tert*-Butyldimethylsilyloxy)-5-hydroxy-3-oxohexanoic acid *tert*-butyl ester

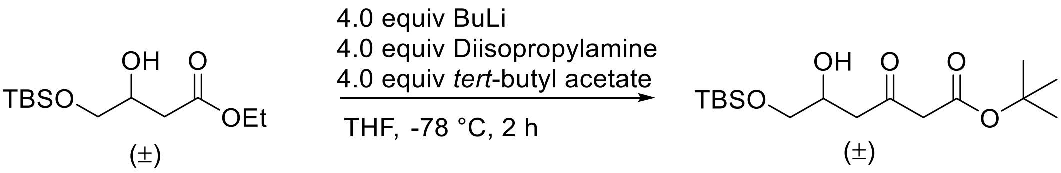

The preparation was following a reported procedure by Carreira (*Angew. Chem., Int. Ed.* **2008**, *47*, 4335.): 4.5 mL (30.5 mmol, 4.0 equiv) of iPr_2NH dissolved in 20 mL of anhydrous THF was cooled to −78 °C and 19.06 mL (30.5 mmol, 4.0 equiv) of a 1.6 M *n*-BuLi solution in hexane was added via dropping funnel over 45 min. After stirring at −78 °C for 30 min, 3.54 g (30.5 mmol, 4.0 equiv) of *tert*-butyl acetate was added via dropping funnel over 45 min. The reaction mixture was stirred

for an additional 30 min and then 2 g (7.63 mmol, 1 equiv) of the starting hydroxy ester dissolved in 10 mL THF was added via a dropping funnel over 45 min. The temperature was allowed to rise until −55 °C during the addition. The cooling bath was removed and the reaction mixture was allowed to warm to −10 °C over 4 h. Then 50 mL of 10% HCl was added over 30 min from a dropping funnel. The temperature was allowed to reach 10 °C during the addition and the pH reached 2. The reaction mixture was transferred into a separation funnel and the phases were separated. The combined aqueous phases were extracted with EtOAc (30 mL × 3). The combined organic layers were washed successively with saturated $NaHCO_3$ (30 mL) and NaCl (30 mL) before being dried over Na_2SO_4 and concentrated in vacuo to give a viscous pale yellow oil. Flash chromatography with 10:1 hexanes/AcOEt provided the desired product as a colorless oil.

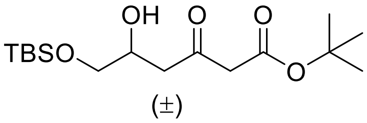

Colorless oil (1.65 g, 64% yield), R_f = 0.29 (5:1 hexanes/ AcOEt). ^{1}H NMR (600 MHz, $CDCl_3$) δ: 4.02–4.01 (m, 1H, C**H**OH), 3.49–3.47 (m, 2H, C**H$_2$**OTBS), 3.31 (s, 2H, COC**H$_2$**CO), 2.94 (s, 1H, OH), 2.62–2.60 (m, 2H, CHOHC**H$_2$**CO), 1.38–1.29 (s, 9H, OC(C**H$_3$**)$_3$), 0.78 (s, 9H, C(C**H$_3$**)$_3$), −0.03 (s, 6H, Si(C**H$_3$**)$_2$).

(3*S,5*R**)-6-(*tert*-Butyldimethylsilyloxy)-3,5-dihydroxyhexanoic acid *tert*-butyl ester**

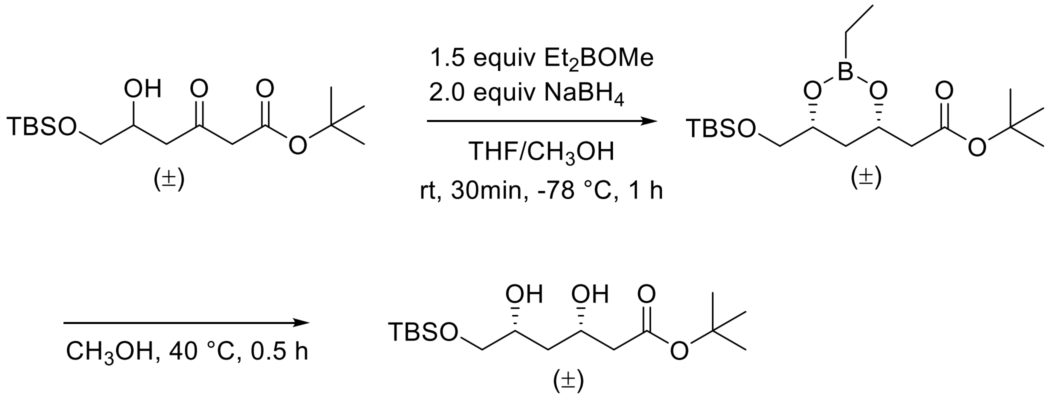

To a solution of the starting β-keto-δ-hydroxy ester (615 mg, 1.85 mmol) in THF (10 mL) and MeOH (2 mL) was added Et_2BOMe (2.78 mL, 2.78 mmol, 1 mol/L in THF), and the solution was stirred for 30 min at room temperature before being cooled to −78 °C. $NaBH_4$ (140 mg, 3.7 mmol) was added portionwise over 10 min (moderate gas evolution observed). After the solution was stirred for 1 h at −78 °C, 20 mL of sat. aq. NH_4Cl was added to the cold solution. The mixture was

allowed to warm to room temperature and then extracted with EtOAc (50 mL × 3). The combined organic layers were washed successively with saturated NaCl (20 mL) before being dried over Na_2SO_4 and concentrated in vacuo to give a viscous pale yellow oil. MeOH (10 mL) was added, the solution was concentrated via rotary evaporation at ca. 40 °C. This was repeated for 4 times. The mixture was concentrated and purified by column chromatography.

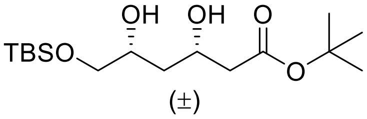

Colorless oil (587 mg, 95% yield), R_f = 0.29 (5:1 hexanes/ AcOEt). ^{1}H NMR (600 MHz, $CDCl_3$) δ: 4.19–4.18 (s, 1H, CHO**H**), 3.85 (s, 2H, C**H₂**OTBS), 3.52–3.45 (m, 2H, two C**H**OH), 3.26–3.25 (m, 1H, O**H**), 2.43–2.33 (m, 2H, C**H₂**COOC(CH₃)₃), 1.63–1.47 (m, 2H, OHCHC**H₂**CHOH), 1.41 (s, 9H, OC(C**H₃**)₃), 0.85 (s, 9H, SiC(C**H₃**)₃), 0.022 (s, 6H, Si(C**H₃**)₂).

[(4*S,6*R**)-6-(*tert*-Butyldimethylsilyloxymethyl-2,2-dimethyl-1,3-dioxan-4-yl]acetic acid *tert*-butyl ester**

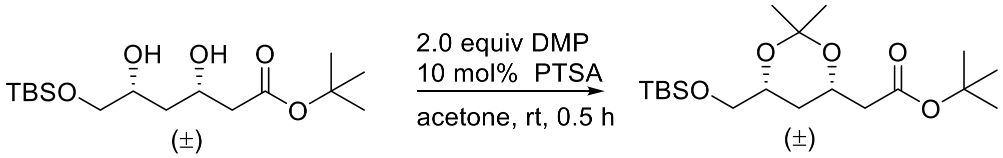

To a solution of the dihydroxy ester (861 mg, 2.57 mmol) in acetone (6 mL) were added 2,2-dimethoxpropane (536 mg, 5.1 mmol) and *p*-toluenesulfonic acid monohydrate (44 mg, 0.26 mmol, 0.1 equiv). The resulting mixture was stirred at room temperature until TLC analysis indicated consumption of the starting material (0.5 h). Et_3N (3 mL) was added, and the mixture was concentrated and purified by column chromatography.

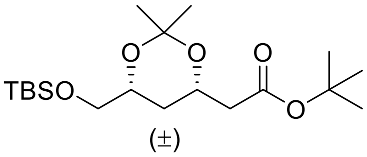

Colorless oil (904 mg, 94% yield), R_f = 0.67 (5:1 hexanes/ AcOEt). ^{1}H NMR (600 MHz, $CDCl_3$) δ: 4.21–4.20 (m, 1H, one of C**H₂**OTBS), 3.89–3.87 (m, 1H, one of C**H₂**OTBS), 3.63–3.39 (m, 2H, OC**H**CH₂C**H**O), 2.41–2.23 (m, 2H, C**H**₂COOC(CH₃)₃), 1.61–1.59 (m, 1H, one of OCHC**H**₂CHO), 1.40–1.39 (m, 9H, OC(C**H**₃)₃), 1.31 (s, 6H, C(C**H**₃)₃), 1.15–1.09 (m, 1H, one of OCHC**H**₂CHO), 0.83 (s, 9H, C(C**H**₃)₃), –0.01 (s, 6H, Si(C**H**₃)₂).

[(4*S,6*R**)-2,2-Dimethyl-6-hydroxymethyl-1,3-dioxan-4-yl]acetic acid *tert*-butyl ester**

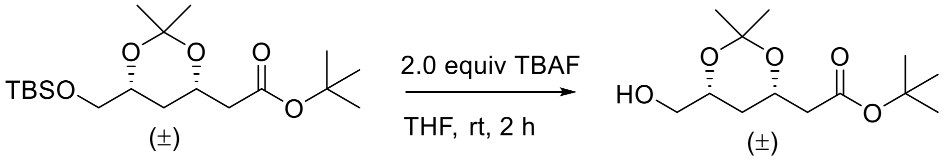

To a stirred solution of the TBS ether (581 mg, 1.55 mmol) in THF (4 mL) was added 1 M tetrabutylammonium fluoride in THF (3.1 mL, 3.1 mmol, 2 equiv), and the reaction mixture was stirred at room temperature for 2 h. The reaction was quenched with aqueous NH_4Cl. The layers were separated, and the aqueous layer was extracted with AcOEt. The combined organic layers were washed with brine and dried over Na_2SO_4. After evaporation of the solvent under reduced pressure, the residue was purified by column chromatography.

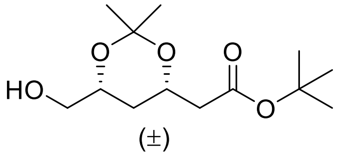

Colorless oil (346 mg, 86% yield), R_f = 0.42 (1:1 hexanes/ AcOEt). ^{1}H NMR (400 MHz, $CDCl_3$) δ: 4.24–3.89 (m, 2H, OHC**H**$_2$), 3.52–3.39 (m, 2H, C**H**OC(CH_3)$_2$ and C**H**OC(CH_3)$_2$), 2.62 (s, 1H, OH), 2.39–2.20 (m, 2H, C**H**$_2$COOC(CH_3)$_3$), 1.44–1.41 (m, 1H, one of OCHC**H**$_2$CHO), 1.39 (s, 3H, C(C**H**$_3$)$_2$), 1.36 (s, 9H, C(CH_3)$_3$), 1.30 (s, 3H, C(C**H**$_3$)$_2$), 1.26–1.17 (m, 1H, one of OCHC**H**$_2$CHO).

[(4*S,6*R**)-6-Benzoyloxymethyl-2,2-dimethyl-1,3-dioxan-4-yl]acetic acid *tert*-butyl ester**

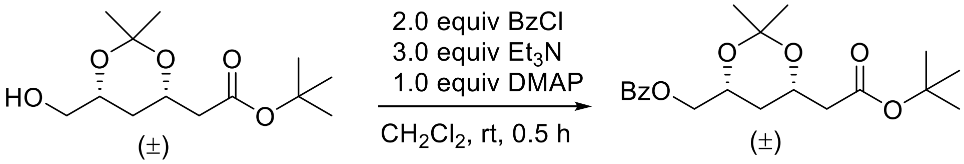

To a solution of the starting material (82 mg, 0.31 mmol) in CH_2Cl_2 (4 mL) were added Et_3N (130 μL, 0.95 mmol), DMAP (38 mg, 0.31 mmol) and benzoyl chloride (71 μL, 0.62 mmol). After stirring for 0.5 h at room temperature, the mixture was purified by column chromatography directly.

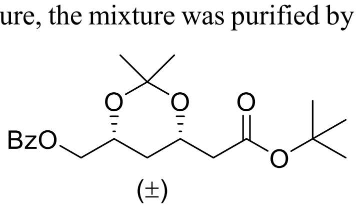

Colorless oil (103 mg, 92% yield), R_f = 0.37 (5:1 hexanes/ AcOEt). ^{1}H NMR (400 MHz, $CDCl_3$) δ: 8.04–8.02 (m, 2H, ArH), 7.56–7.52 (m 1H, ArH), 7.44–7.41 (m, 2H, ArH), 4.32–4.23 (m, 4H, C**H**OC(CH_3)$_2$ and C**H**OC(CH_3)$_2$ and BzOC**H**$_2$), 2.48–2.31 (m, 2H, C**H**$_2$COOC(CH_3)$_3$), 1.68–1.64 (m, 1H, one of OCHC**H**$_2$CHO) 1.48 (s, 3H, C(C**H**$_3$)$_2$), 1.43 (s, 9H, C(CH_3)$_3$), 1.40 (s, 3H, C(C**H**$_3$)$_2$), 1.38–1.32 (m, 1H, one of OCHC**H**$_2$CHO).

3. Crystallographic data of compound 17

Crystals of compound **17** were grown in hexanes at 4 °C by slow evaporation of the solvents. The ellipsoid contour percent probability level was 50%.

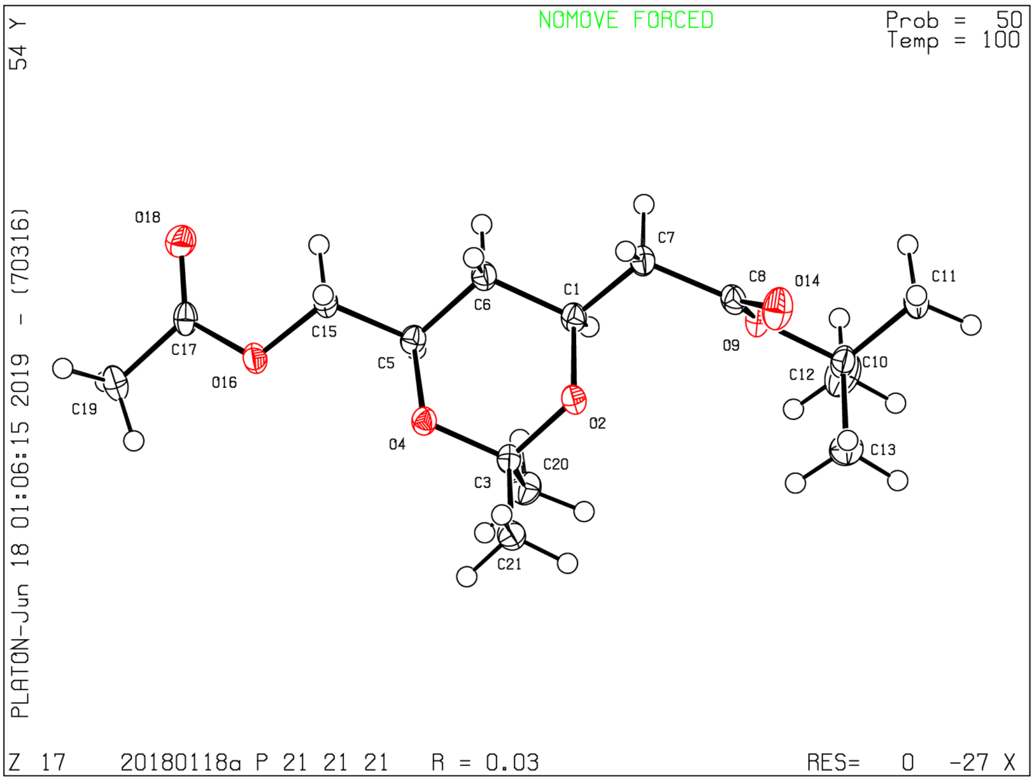

Bond precision:	C-C = 0.0029 A		Wavelength=1.54184
Cell:	a=5.6812(2)	b=12.9135(4)	c=22.1615(6)
	alpha=90	beta=90	gamma=90
Temperature:	100 K		

	Calculated	Reported
Volume	1625.86(9)	1625.86(9)
Space group	P 21 21 21	P 21 21 21
Hall group	P 2ac 2ab	P 2ac 2ab
Moiety formula	C15 H26 O6	C15 H26 O6
Sum formula	C15 H26 O6	C15 H26 O6
Mr	302.36	302.36
Dx,g cm-3	1.235	1.235
Z	4	4
Mu (mm-1)	0.785	0.785
F000	656.0	656.0

F000'	658.20	
h,k,lmax	7,16,27	6,15,27
Nref	3255[1908]	3203
Tmin,Tmax	0.954,0.962	0.453,1.000
Tmin'	0.855	

Correction method= # Reported T Limits: Tmin=0.453 Tmax=1.000 AbsCorr = MULTI-SCAN

Data completeness= 1.68/0.98 Theta(max)= 73.182

R(reflections)= 0.0339(3043) wR2(reflections)= 0.0891(3203)

S = 1.086 Npar= 196

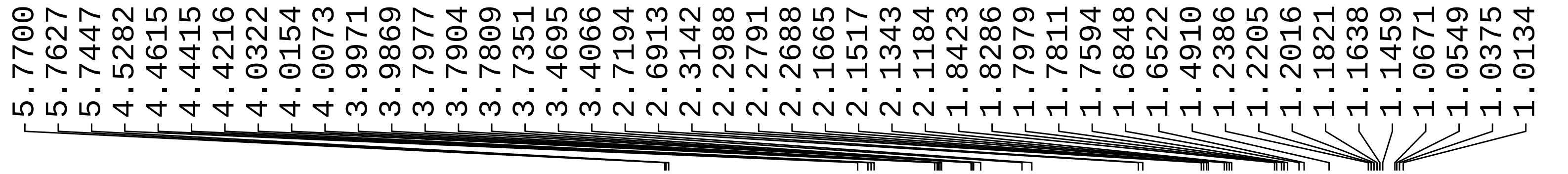

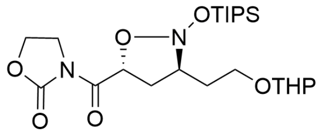

1

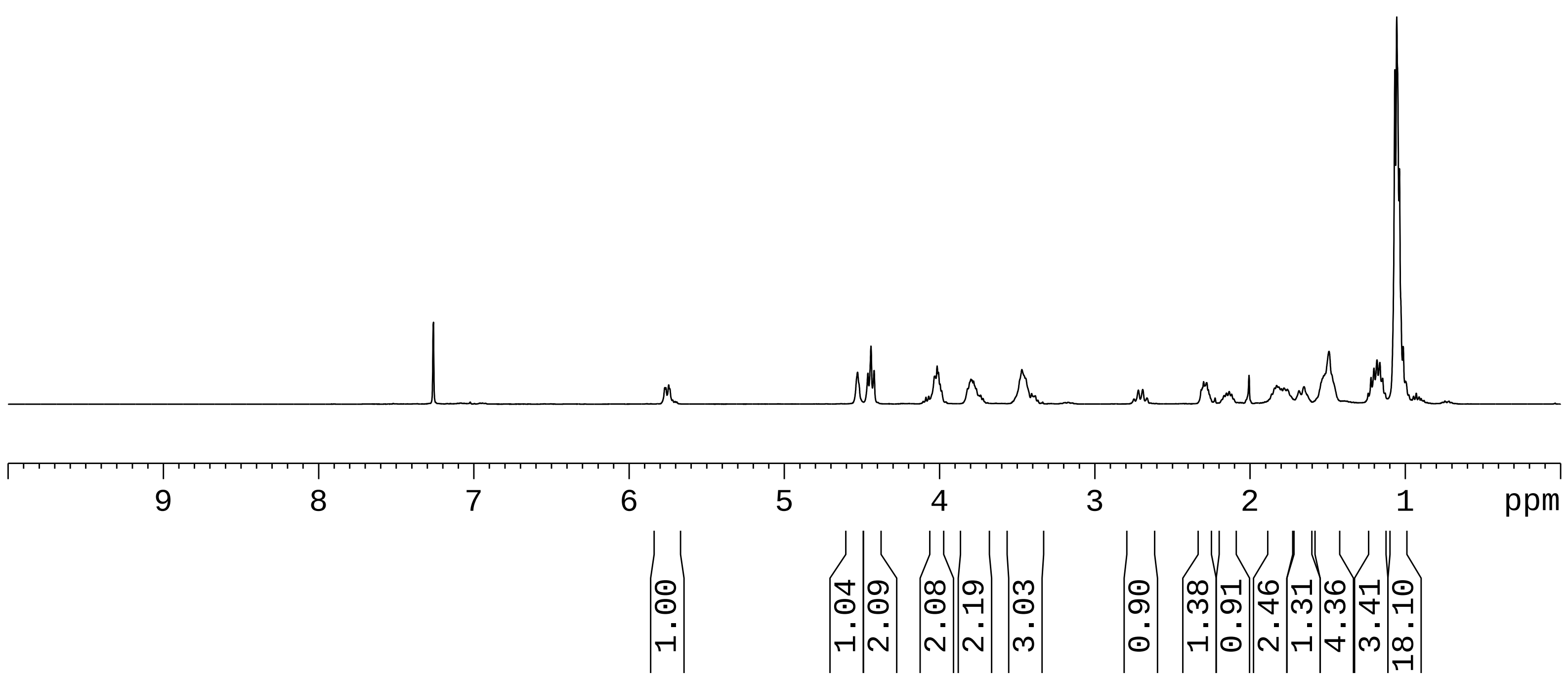

NAME	JMH161215
EXPNO	1
PROCNO	1
Date_	20161217
Time	10.33
INSTRUM	spect
PROBHD	5 mm PABBO BB-
PULPROG	zg30
TD	65536
SOLVENT	CDCl3
NS	16
DS	2
SWH	8223.685 Hz
FIDRES	0.125483 Hz
AQ	3.9846387 sec
RG	40.3
DW	60.800 usec
DE	6.50 usec
TE	291.6 K
D1	1.00000000 sec
TD0	1

======== CHANNEL f1 ========

NUC1	1H
P1	13.80 usec
PL1	-1.00 dB
PL1W	13.18669796 W
SFO1	400.1724712 MHz
SI	32768
SF	400.1700025 MHz
WDW	EM
SSB	0
LB	0.30 Hz
GB	0
PC	1.00

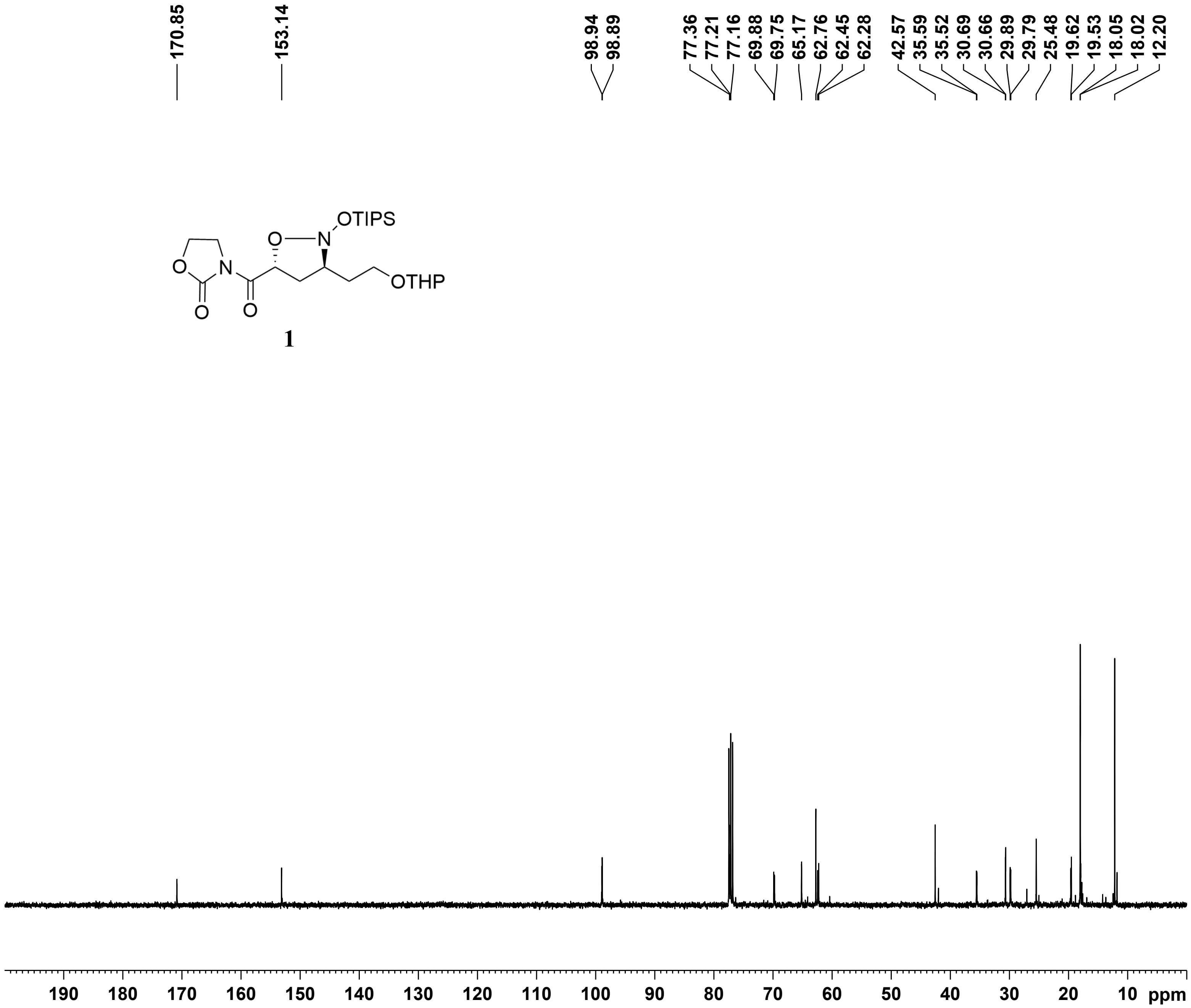

1

NAME	JMH161215-C13	
EXPNO	1	
PROCNO	1	
Date_	20161217	
Time	10.39	
INSTRUM	spect	
PROBHD	5 mm PABBO BB-	
PULPROG	zgpg30	
TD	65536	
SOLVENT	CDCl3	
NS	148	
DS	4	
SWH	24038.461	Hz
FIDRES	0.366798	Hz
AQ	1.3631988	sec
RG	203	
DW	20.800	usec
DE	6.50	usec
TE	292.5	K
D1	2.00000000	sec
D11	0.03000000	sec
TD0	1	
======== CHANNEL f1 ========		
NUC1	13C	
P1	8.50	usec
PL1	-2.00	dB
PL1W	57.32743073	W
SFO1	100.6328888	MHz
======== CHANNEL f2 ========		
CPDPRG2	waltz16	
NUC2	1H	
PCPD2	80.00	usec
PL2	-1.00	dB
PL12	14.26	dB
PL13	14.46	dB
PL2W	13.18669796	W
PL12W	0.39276794	W
PL13W	0.37509048	W
SFO2	400.1716007	MHz
SI	32768	
SF	100.6228201	MHz
WDW	EM	
SSB	0	
LB	1.00	Hz
GB	0	
PC	1.40	

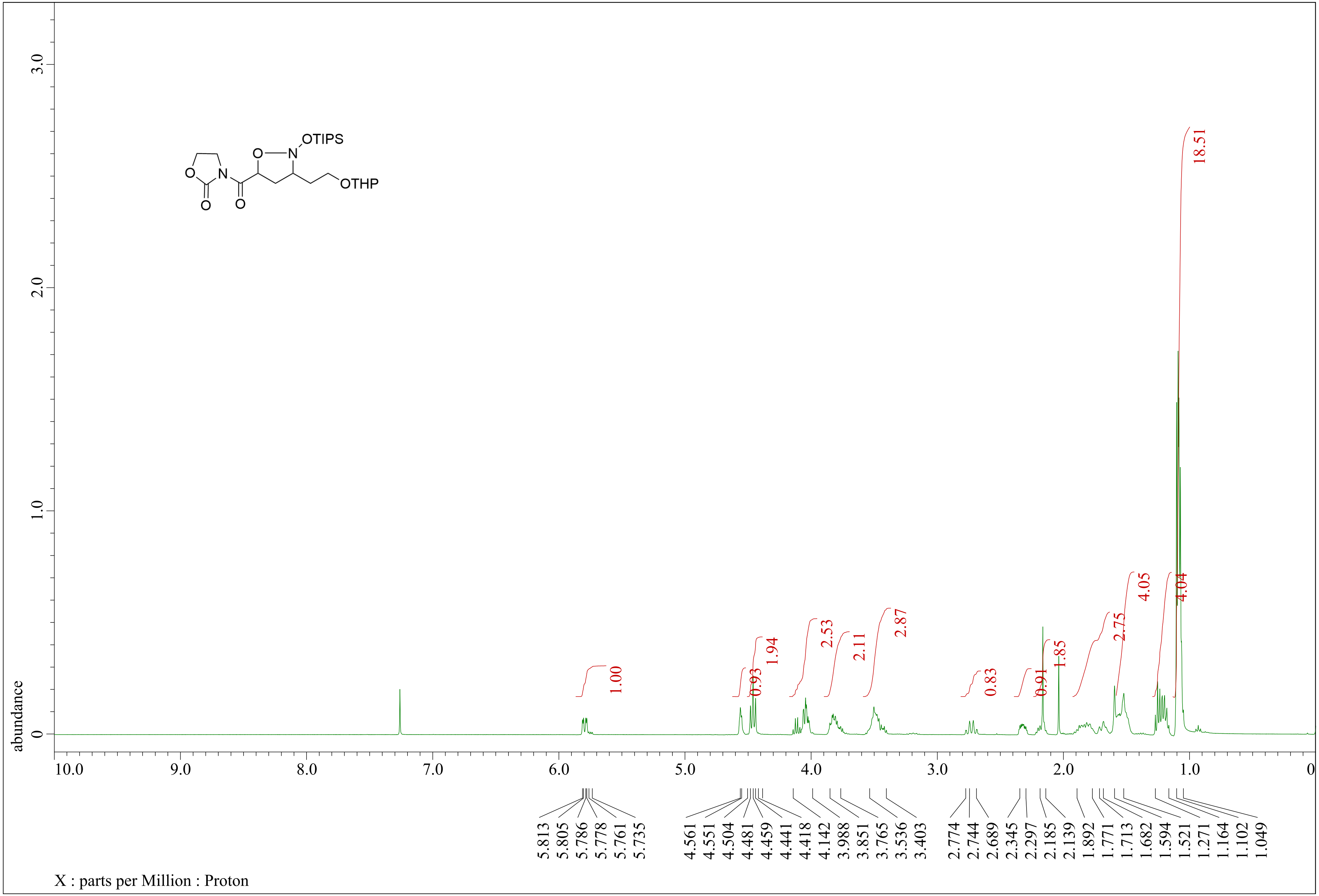
OTIPS
OTHP
abundance
X : parts per Million : Proton
18.51
4.05
4.04
2.75
2.87
2.53
2.11
1.94
1.85
1.00
0.93
0.91
0.83
5.813 5.805 5.786 5.778 5.761 5.735
4.561 4.551 4.504 4.481 4.459 4.441 4.418 4.142 3.988 3.851 3.765 3.536 3.403
2.774 2.744 2.689 2.345 2.297 2.185 2.139 1.892 1.771 1.713 1.682 1.594 1.521 1.271 1.164 1.102 1.049

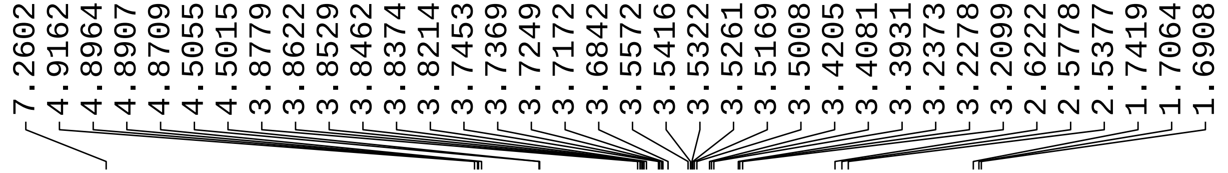

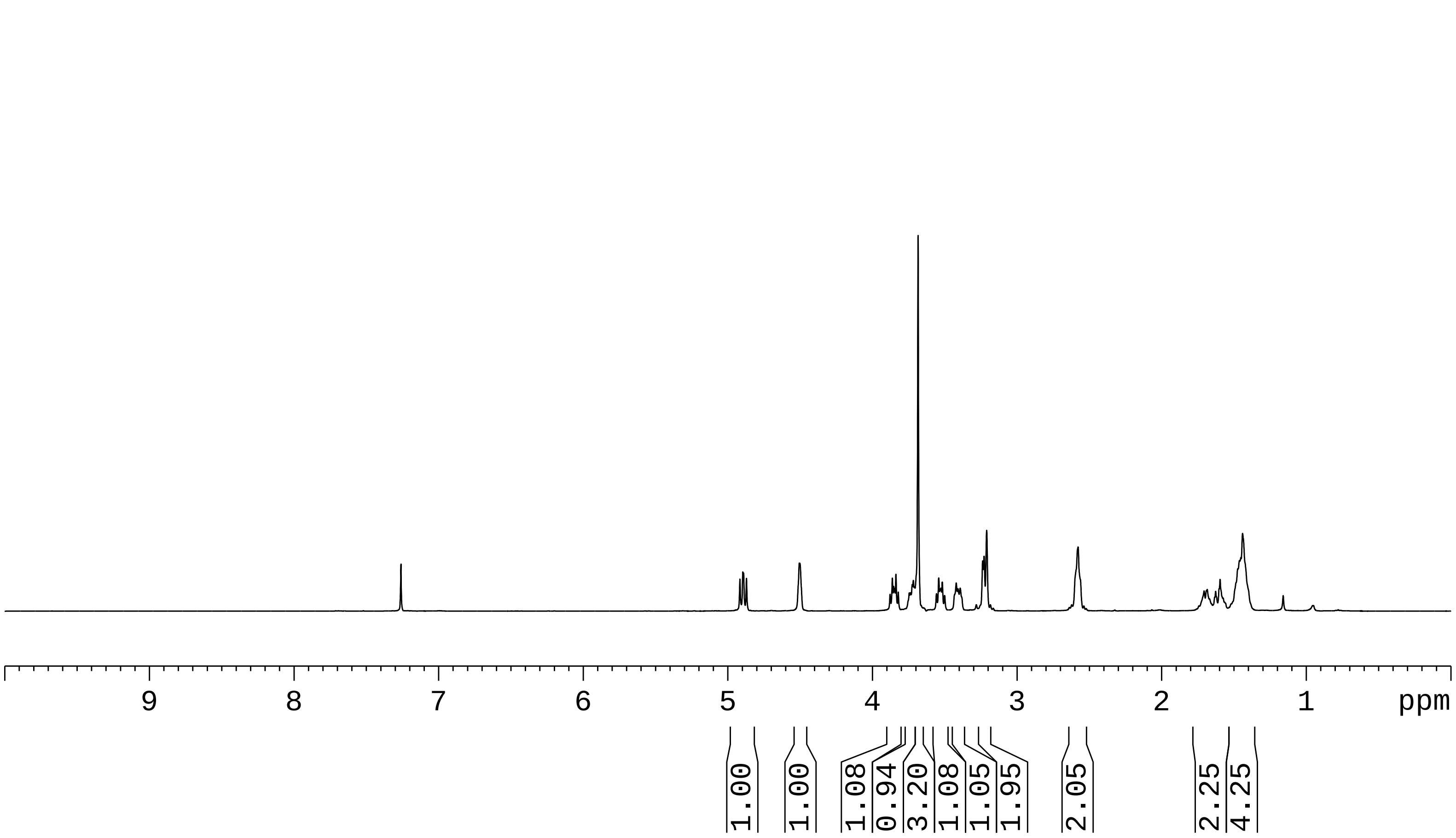

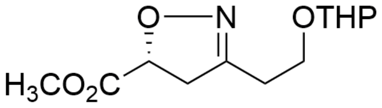

3

NAME	FJJ170510-5
EXPNO	1
PROCNO	1
Date_	20170515
Time	15.59
INSTRUM	spect
PROBHD	5 mm PABBO BB-
PULPROG	zg30
TD	65536
SOLVENT	CDCl3
NS	16
DS	2
SWH	8223.685 Hz
FIDRES	0.125483 Hz
AQ	3.9846387 sec
RG	36
DW	60.800 usec
DE	6.50 usec
TE	301.3 K
D1	1.00000000 sec
TD0	1
======== CHANNEL f1 ========	
NUC1	1H
P1	13.80 usec
PL1	-1.00 dB
PL1W	13.18669796 W
SFO1	400.1724712 MHz
SI	32768
SF	400.1700020 MHz
WDW	EM
SSB	0
LB	0.30 Hz
GB	0
PC	1.00

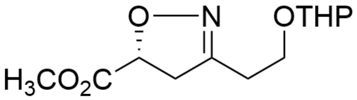

3

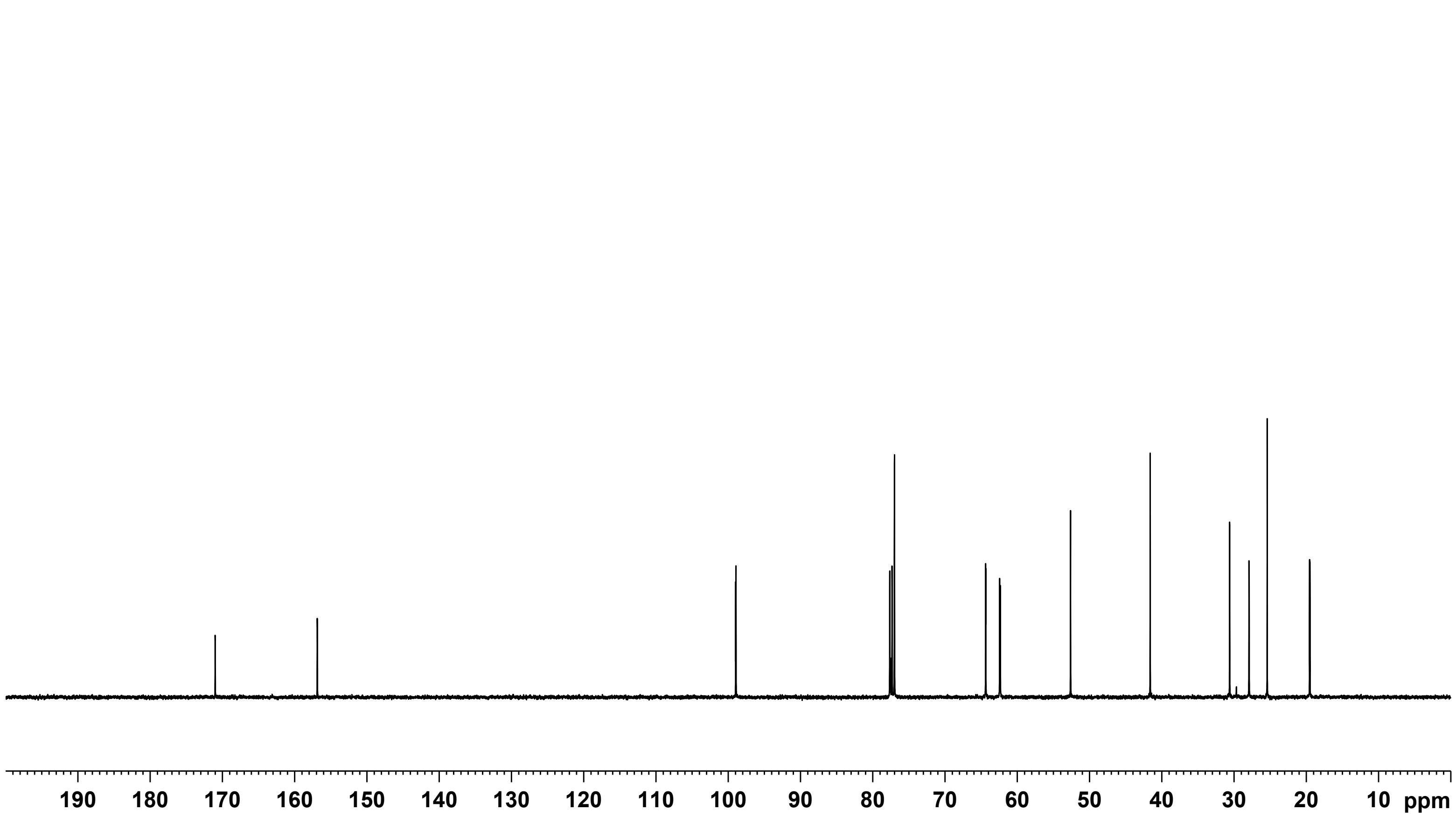

NAME	FJJ170510-5-C13	
EXPNO	1	
PROCNO	1	
Date_	20170515	
Time	16.10	
INSTRUM	spect	
PROBHD	5 mm PABBO BB-	
PULPROG	zgpg30	
TD	65536	
SOLVENT	CDCl3	
NS	137	
DS	4	
SWH	24038.461	Hz
FIDRES	0.366798	Hz
AQ	1.3631988	sec
RG	203	
DW	20.800	usec
DE	6.50	usec
TE	302.2	K
D1	2.00000000	sec
D11	0.03000000	sec
TD0	1	
======== CHANNEL f1 ========		
NUC1	13C	
P1	8.50	usec
PL1	-2.00	dB
PL1W	57.32743073	W
SFO1	100.6328888	MHz
======== CHANNEL f2 ========		
CPDPRG2	waltz16	
NUC2	1H	
PCPD2	80.00	usec
PL2	-1.00	dB
PL12	14.26	dB
PL13	14.46	dB
PL2W	13.18669796	W
PL12W	0.39276794	W
PL13W	0.37509048	W
SFO2	400.1716007	MHz
SI	32768	
SF	100.6228121	MHz
WDW	EM	
SSB	0	
LB	1.00	Hz
GB	0	
PC	1.40	

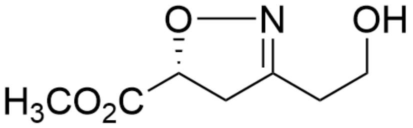

4

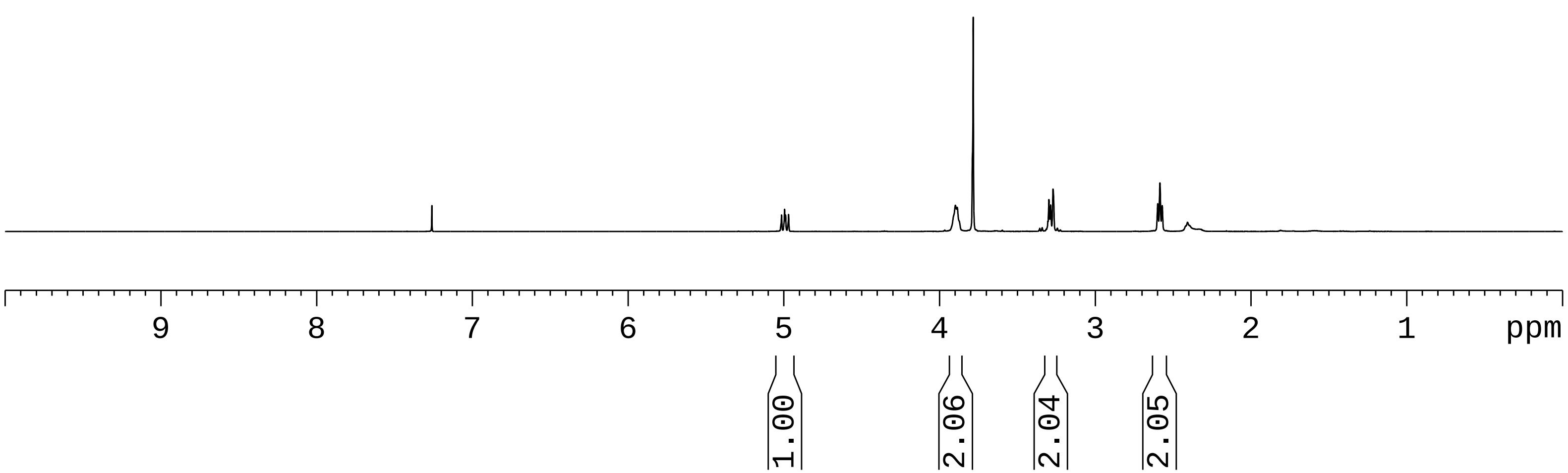

NAME	FJJ170336-3	
EXPNO	1	
PROCNO	1	
Date_	20170308	
Time	11.34	
INSTRUM	spect	
PROBHD	5 mm PABBO BB-	
PULPROG	zg30	
TD	65536	
SOLVENT	CDCl3	
NS	16	
DS	2	
SWH	8223.685	Hz
FIDRES	0.125483	Hz
AQ	3.9846387	sec
RG	144	
DW	60.800	usec
DE	6.50	usec
TE	294.6	K
D1	1.00000000	sec
TD0	1	

======== CHANNEL f1 ========

NUC1	1H	
P1	13.80	usec
PL1	-1.00	dB
PL1W	13.18669796	W
SFO1	400.1724712	MHz
SI	32768	
SF	400.1700030	MHz
WDW	EM	
SSB	0	
LB	0.30	Hz
GB	0	
PC	1.00	

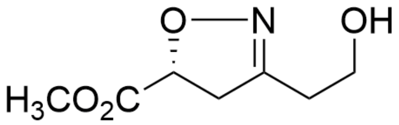

4

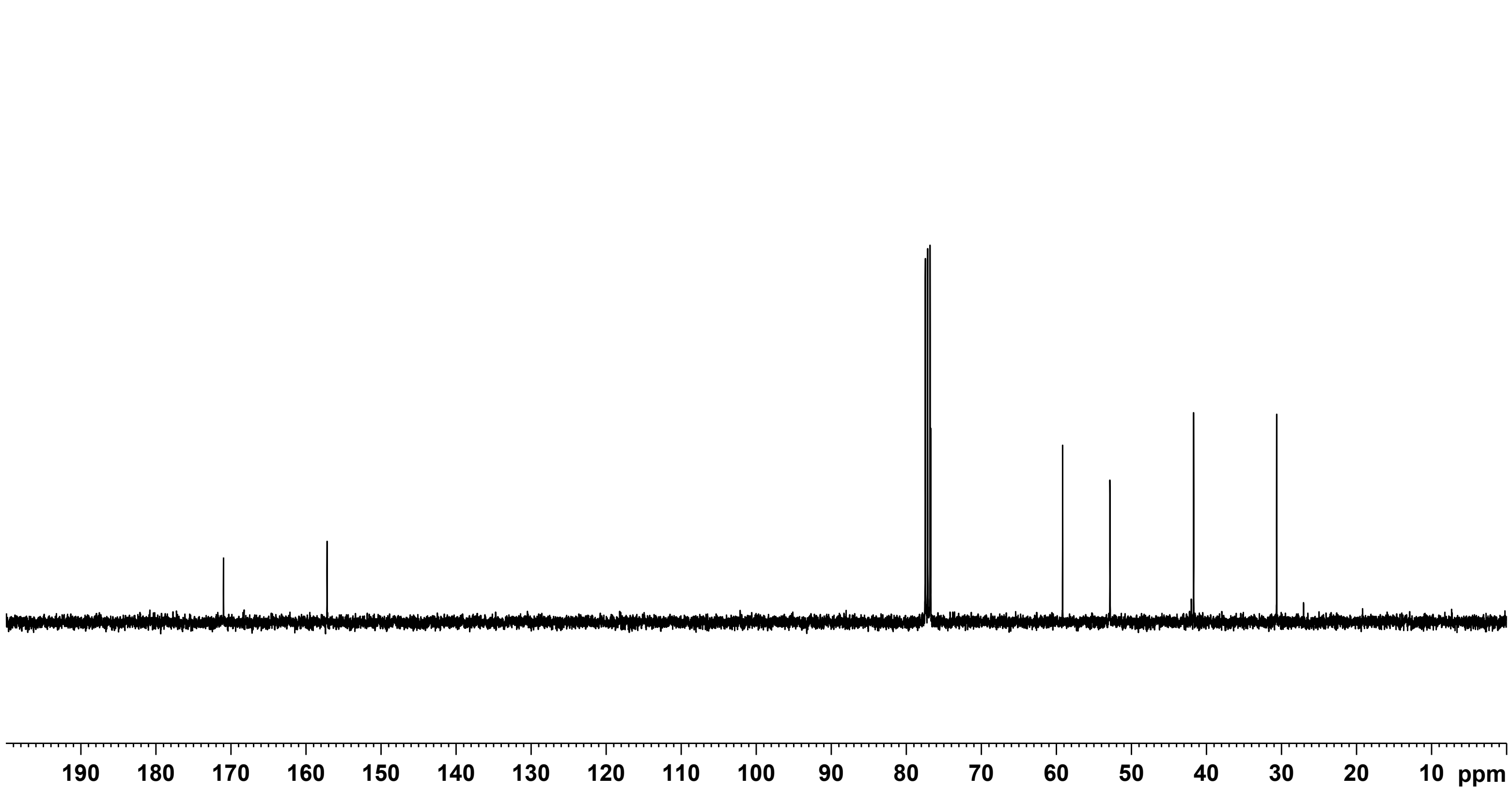

NAME	JMH161218-2-C13	
EXPNO	1	
PROCNO	1	
Date_	20161220	
Time	11.12	
INSTRUM	spect	
PROBHD	5 mm PABBO BB-	
PULPROG	zgpg30	
TD	65536	
SOLVENT	CDCl3	
NS	37	
DS	4	
SWH	24038.461	Hz
FIDRES	0.366798	Hz
AQ	1.3631988	sec
RG	203	
DW	20.800	usec
DE	6.50	usec
TE	292.6	K
D1	2.00000000	sec
D11	0.03000000	sec
TD0	1	
======== CHANNEL f1 ========		
NUC1	13C	
P1	8.50	usec
PL1	-2.00	dB
PL1W	57.32743073	W
SFO1	100.6328888	MHz
======== CHANNEL f2 ========		
CPDPRG2	waltz16	
NUC2	1H	
PCPD2	80.00	usec
PL2	-1.00	dB
PL12	14.26	dB
PL13	14.46	dB
PL2W	13.18669796	W
PL12W	0.39276794	W
PL13W	0.37509048	W
SFO2	400.1716007	MHz
SI	32768	
SF	100.6228201	MHz
WDW	EM	
SSB	0	
LB	1.00	Hz
GB	0	
PC	1.40	

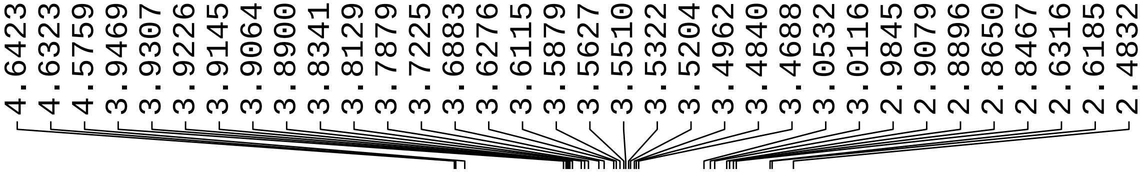

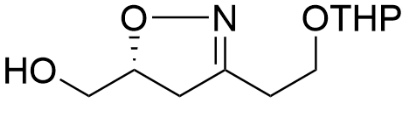

5

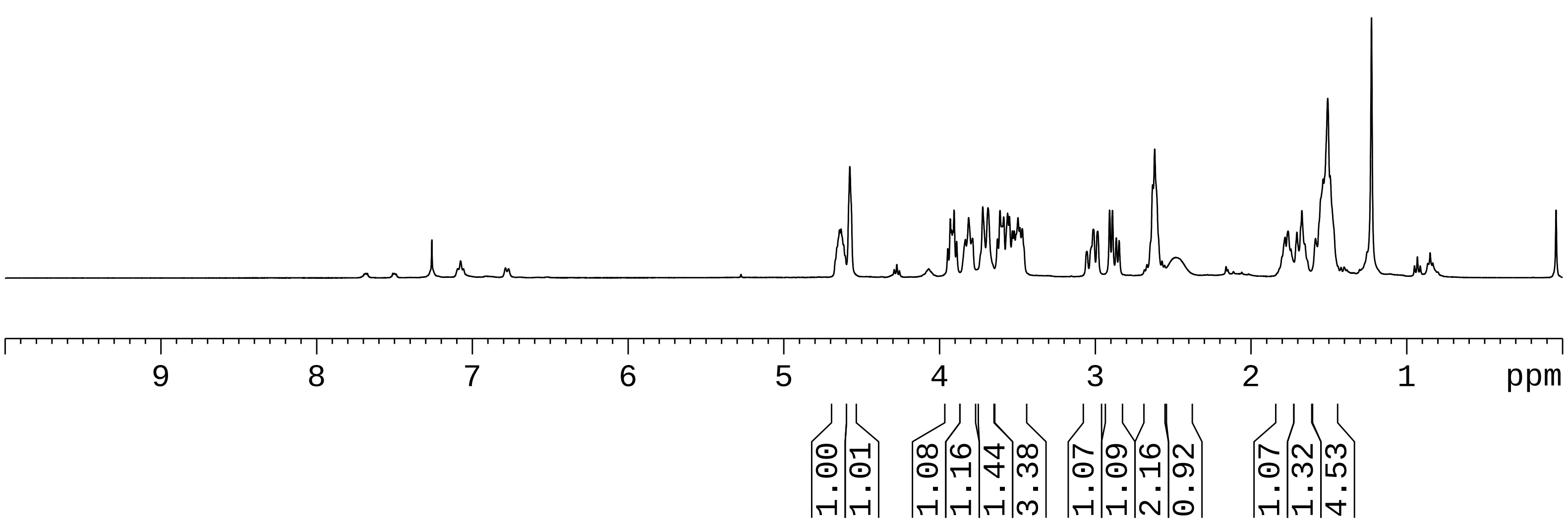

BRUKER

NAME	FJJ170328	
EXPNO	1	
PROCNO	1	
Date_	20170329	
Time	14.49	
INSTRUM	spect	
PROBHD	5 mm PABBO BB-	
PULPROG	zg30	
TD	65536	
SOLVENT	CDCl3	
NS	16	
DS	2	
SWH	8223.685	Hz
FIDRES	0.125483	Hz
AQ	3.9846387	sec
RG	64	
DW	60.800	usec
DE	6.50	usec
TE	299.2	K
D1	1.00000000	sec
TD0	1	

======== CHANNEL f1 ========

NUC1	1H	
P1	13.80	usec
PL1	-1.00	dB
PL1W	13.18669796	W
SFO1	400.1724712	MHz
SI	32768	
SF	400.1700028	MHz
WDW	EM	
SSB	0	
LB	0.30	Hz
GB	0	
PC	1.00	

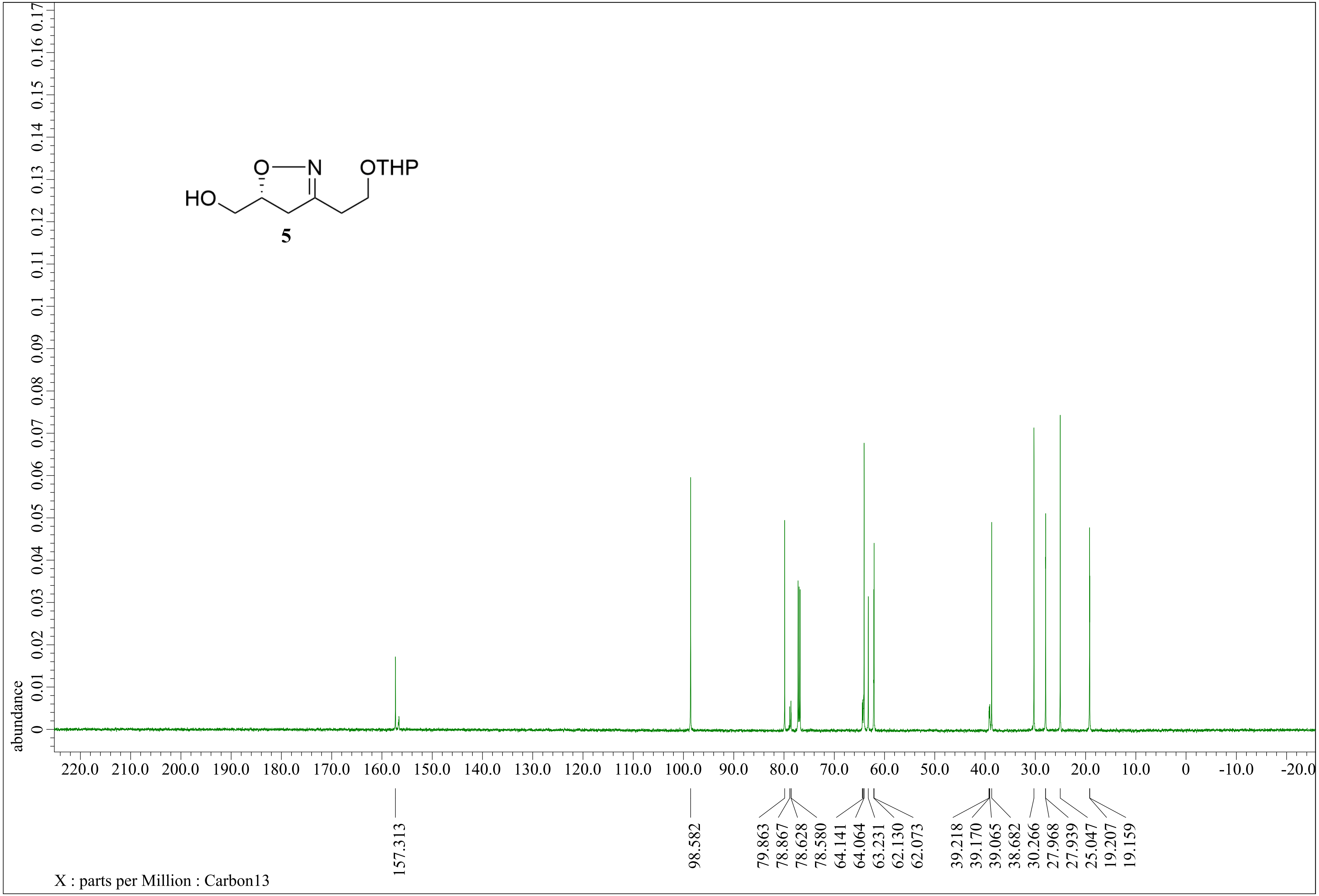

OTHP
HO
O
N
5
abundance
X : parts per Million : Carbon13
157.313
98.582
79.863
78.867
78.628
78.580
64.141
64.064
63.231
62.130
62.073
39.218
39.170
39.065
38.682
30.266
27.968
27.939
25.047
19.207
19.159

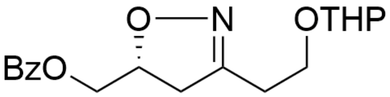

6

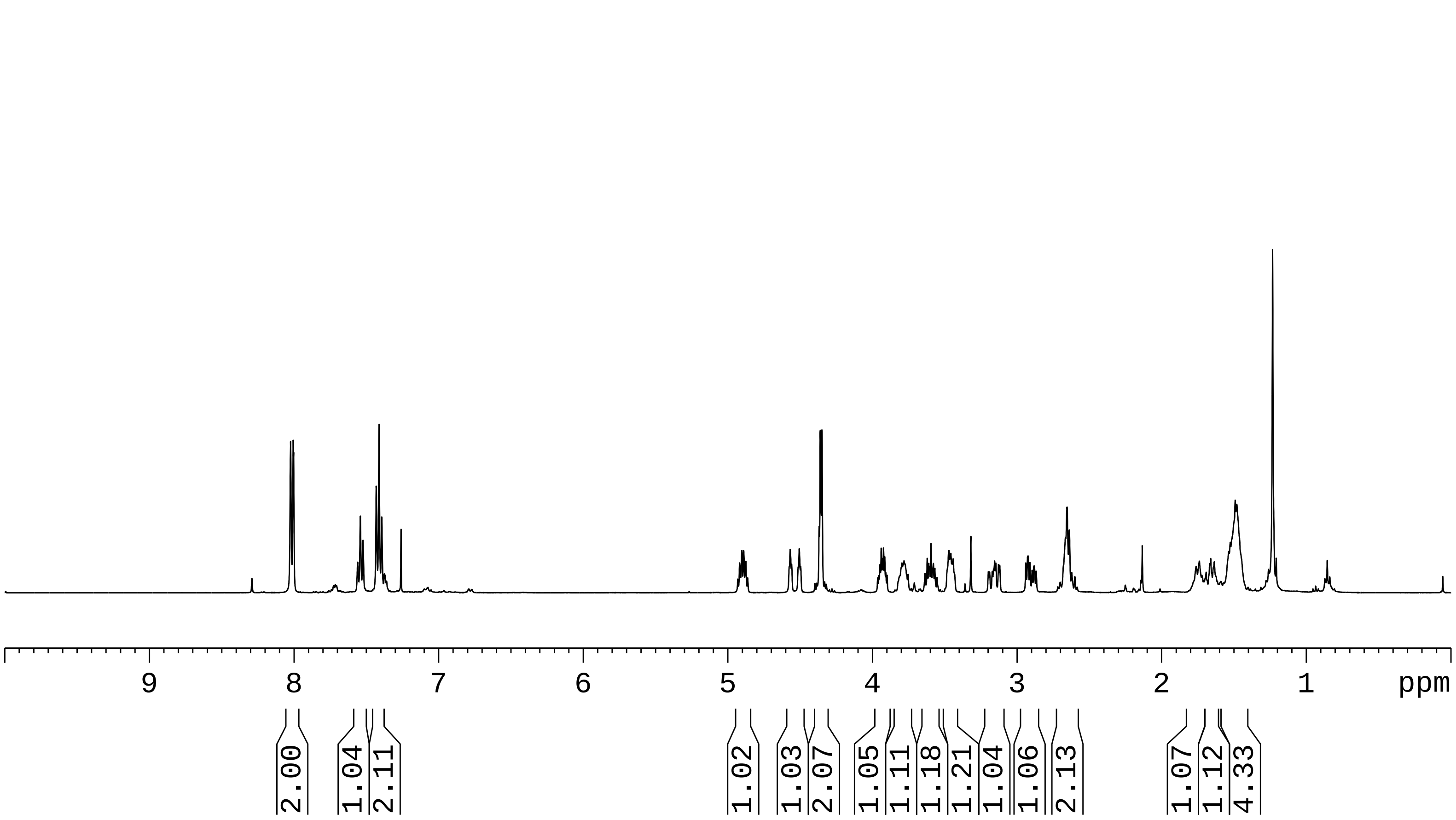

NAME	FJJ170330
EXPNO	1
PROCNO	1
Date_	20170331
Time	22.02
INSTRUM	spect
PROBHD	5 mm PABBO BB-
PULPROG	zg30
TD	65536
SOLVENT	CDCl3
NS	16
DS	2
SWH	8223.685 Hz
FIDRES	0.125483 Hz
AQ	3.9846387 sec
RG	50.8
DW	60.800 usec
DE	6.50 usec
TE	297.3 K
D1	1.00000000 sec
TD0	1

======== CHANNEL f1 ========

NUC1	1H
P1	13.80 usec
PL1	-1.00 dB
PL1W	13.18669796 W
SFO1	400.1724712 MHz
SI	32768
SF	400.1700028 MHz
WDW	EM
SSB	0
LB	0.30 Hz
GB	0
PC	1.00

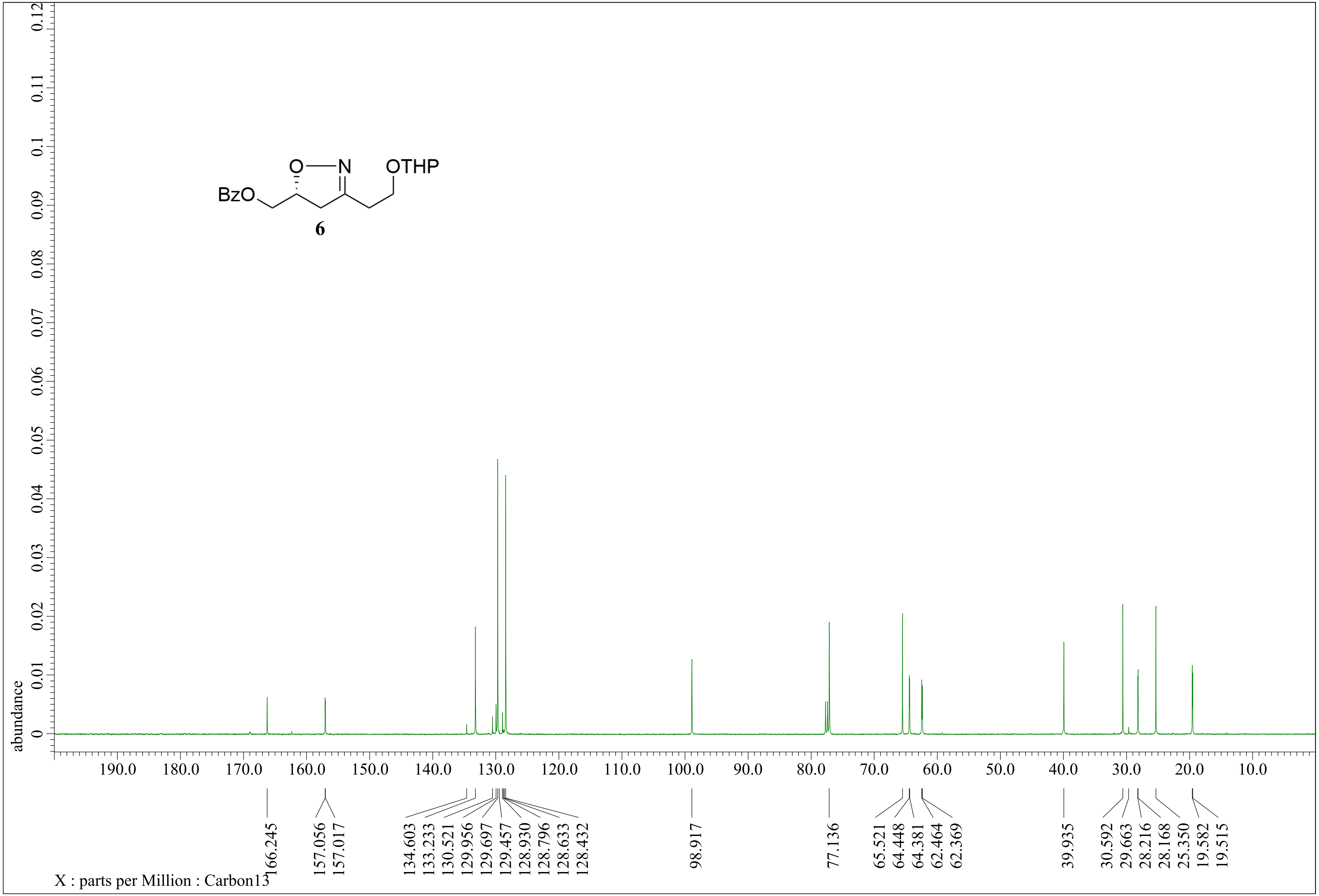
BzO
O
N
OTHP
6
abundance
0
0.01
0.02
0.03
0.04
0.05
0.06
0.07
0.08
0.09
0.1
0.11
0.12
190.0
180.0
170.0
160.0
150.0
140.0
130.0
120.0
110.0
100.0
90.0
80.0
70.0
60.0
50.0
40.0
30.0
20.0
10.0
166.245
157.056
157.017
134.603
133.233
130.521
129.956
129.697
129.457
128.930
128.796
128.633
128.432
98.917
77.136
65.521
64.448
64.381
62.464
62.369
39.935
30.592
29.663
28.216
28.168
25.350
19.582
19.515
X : parts per Million : Carbon13

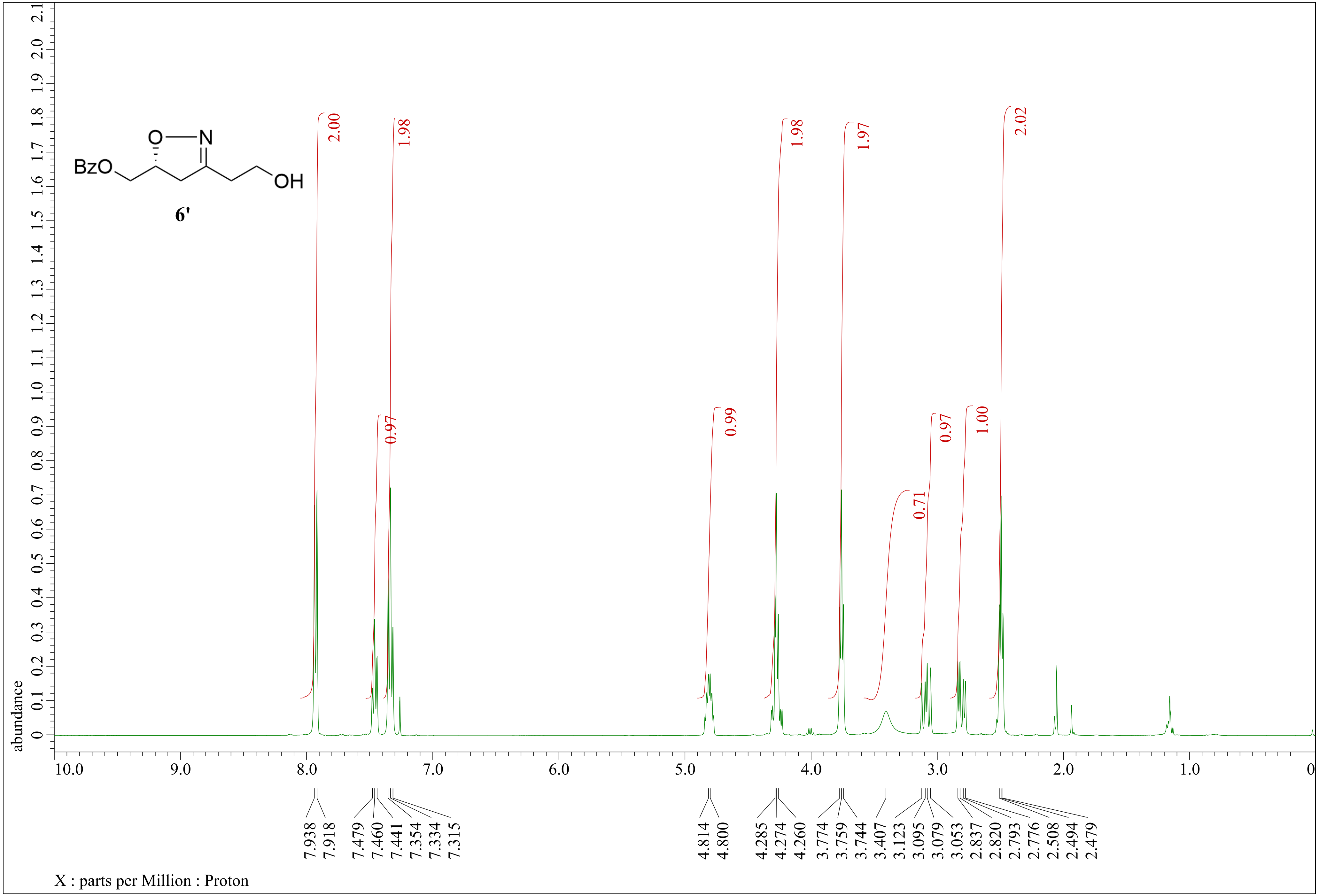
BzO
O
N
OH
6'
2.00
1.98
0.97
0.99
1.98
1.97
0.71
0.97
1.00
2.02
abundance
X : parts per Million : Proton
7.938
7.918
7.479
7.460
7.441
7.354
7.334
7.315
4.814
4.800
4.285
4.274
4.260
3.774
3.759
3.744
3.407
3.123
3.095
3.079
3.053
2.837
2.820
2.793
2.776
2.508
2.494
2.479

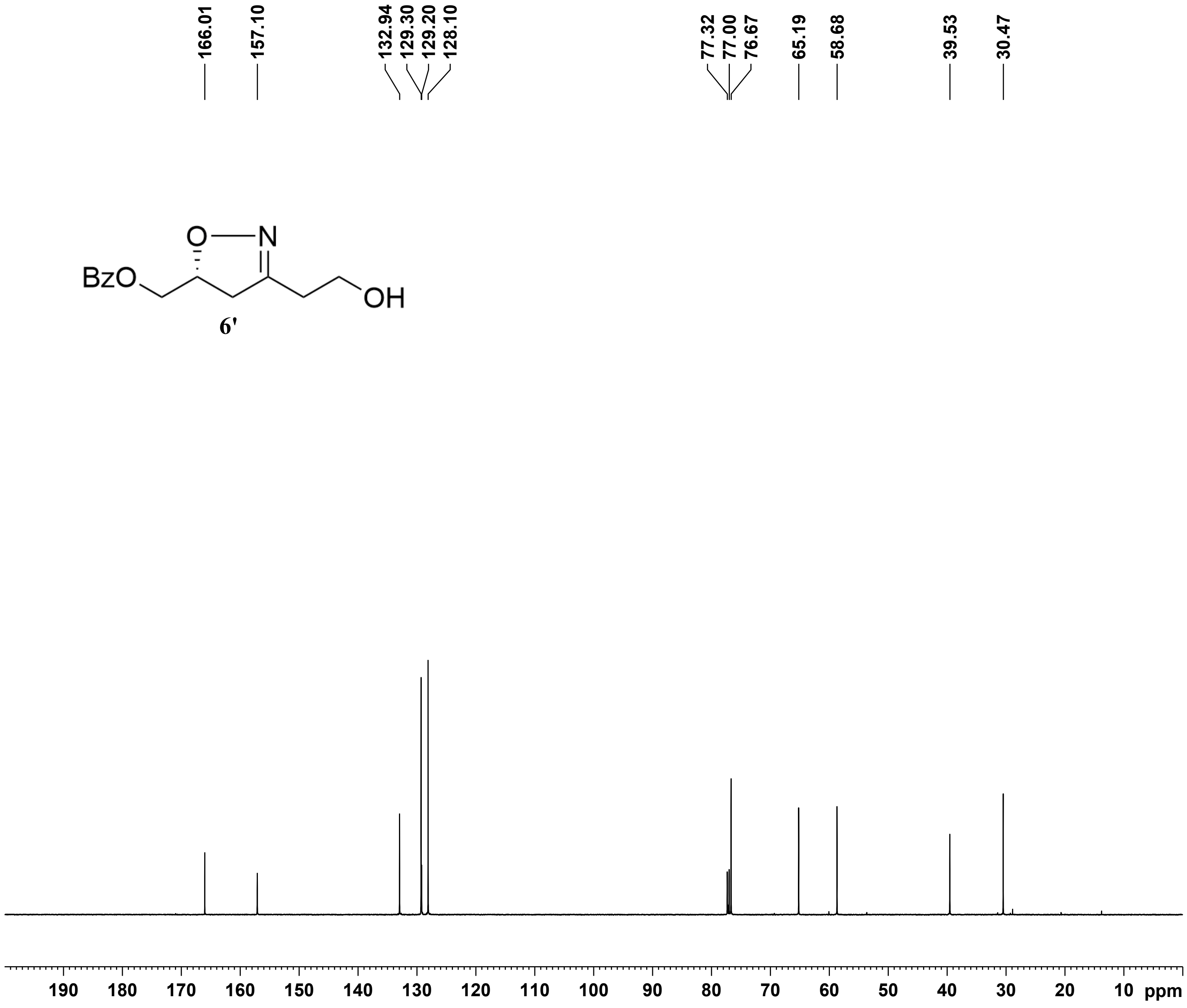

NAME	FJJ170724-C13
EXPNO	1
PROCNO	1
Date_	20170728
Time	11.57
INSTRUM	spect
PROBHD	5 mm PABBO BB-
PULPROG	zgpg30
TD	65536
SOLVENT	CDCl3
NS	400
DS	4
SWH	24038.461 Hz
FIDRES	0.366798 Hz
AQ	1.3631988 sec
RG	203
DW	20.800 usec
DE	6.50 usec
TE	299.0 K
D1	2.00000000 sec
D11	0.03000000 sec
TD0	1

======== CHANNEL f1 ========

NUC1	13C
P1	8.50 usec
PL1	-2.00 dB
PL1W	57.32743073 W
SFO1	100.6328888 MHz

======== CHANNEL f2 ========

CPDPRG2	waltz16
NUC2	1H
PCPD2	80.00 usec
PL2	-1.00 dB
PL12	13.26 dB
PL13	14.46 dB
PL2W	13.18669796 W
PL12W	0.49446553 W
PL13W	0.37509048 W
SFO2	400.1716007 MHz
SI	32768
SF	100.6228581 MHz
WDW	EM
SSB	0
LB	1.00 Hz
GB	0
PC	1.40

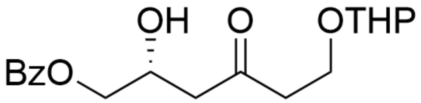

7

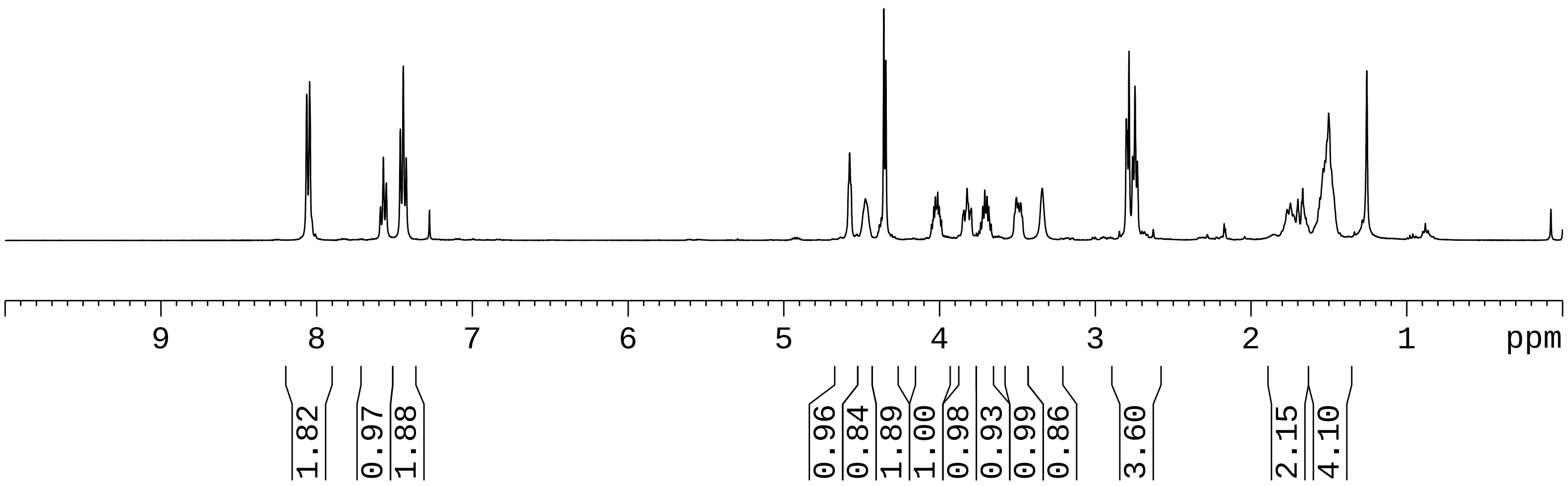

BRUKER

NAME	FJJ170413-XD
EXPNO	1
PROCNO	1
Date_	20170413
Time	21.24
INSTRUM	spect
PROBHD	5 mm PABBO BB-
PULPROG	zg30
TD	65536
SOLVENT	CDCl3
NS	16
DS	2
SWH	8223.685 Hz
FIDRES	0.125483 Hz
AQ	3.9846387 sec
RG	80.6
DW	60.800 usec
DE	6.50 usec
TE	297.6 K
D1	1.00000000 sec
TD0	1

======== CHANNEL f1 ========

NUC1	1H
P1	13.80 usec
PL1	-1.00 dB
PL1W	13.18669796 W
SFO1	400.1724712 MHz
SI	32768
SF	400.1699966 MHz
WDW	EM
SSB	0
LB	0.30 Hz
GB	0
PC	1.00

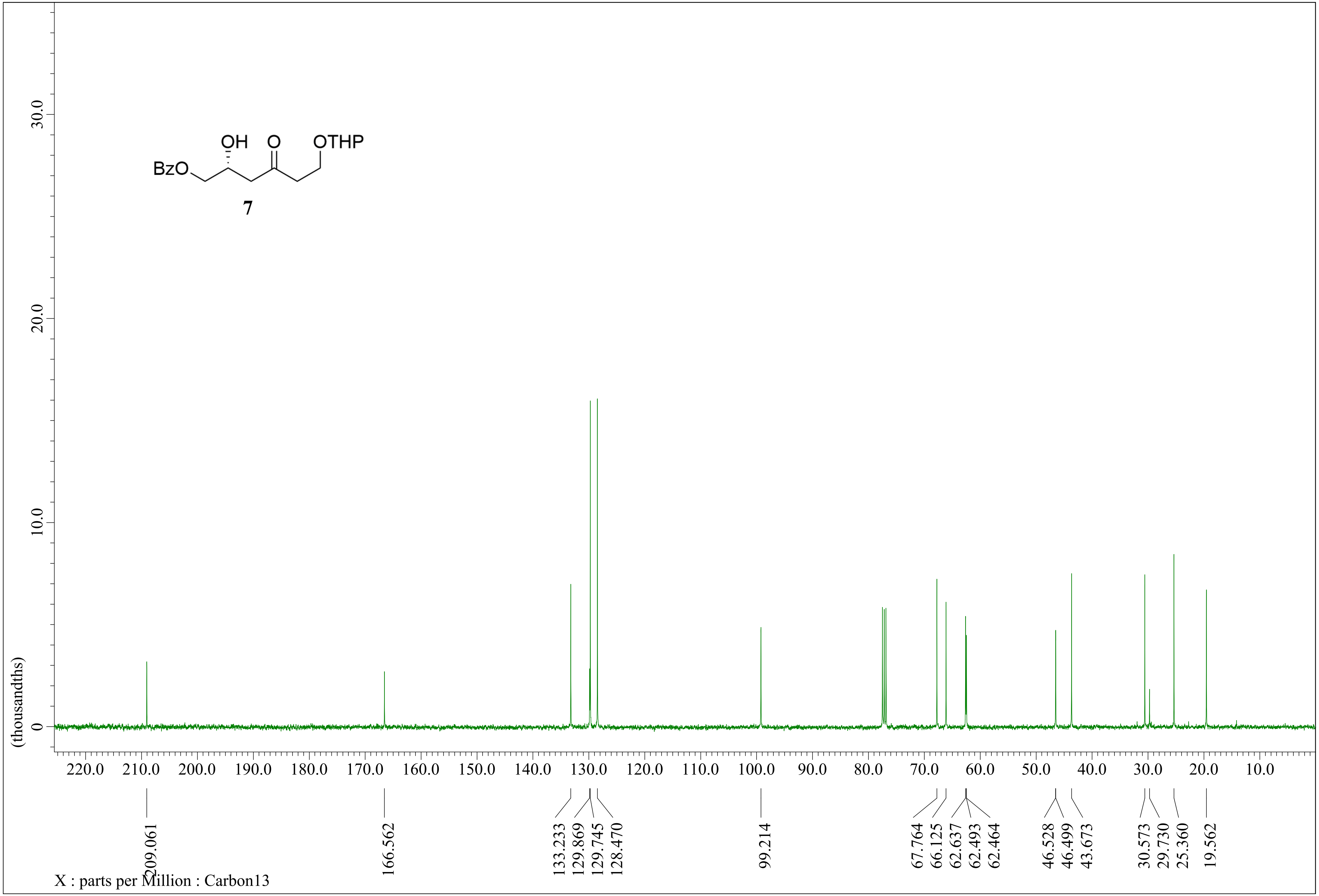

OH
O
OTHP
BzO
7
30.0
20.0
10.0
0
(thousandths)
220.0 210.0 200.0 190.0 180.0 170.0 160.0 150.0 140.0 130.0 120.0 110.0 100.0 90.0 80.0 70.0 60.0 50.0 40.0 30.0 20.0 10.0
209.061
166.562
133.233
129.869
129.745
128.470
99.214
67.764
66.125
62.637
62.493
62.464
46.528
46.499
43.673
30.573
29.730
25.360
19.562
X : parts per Million : Carbon13

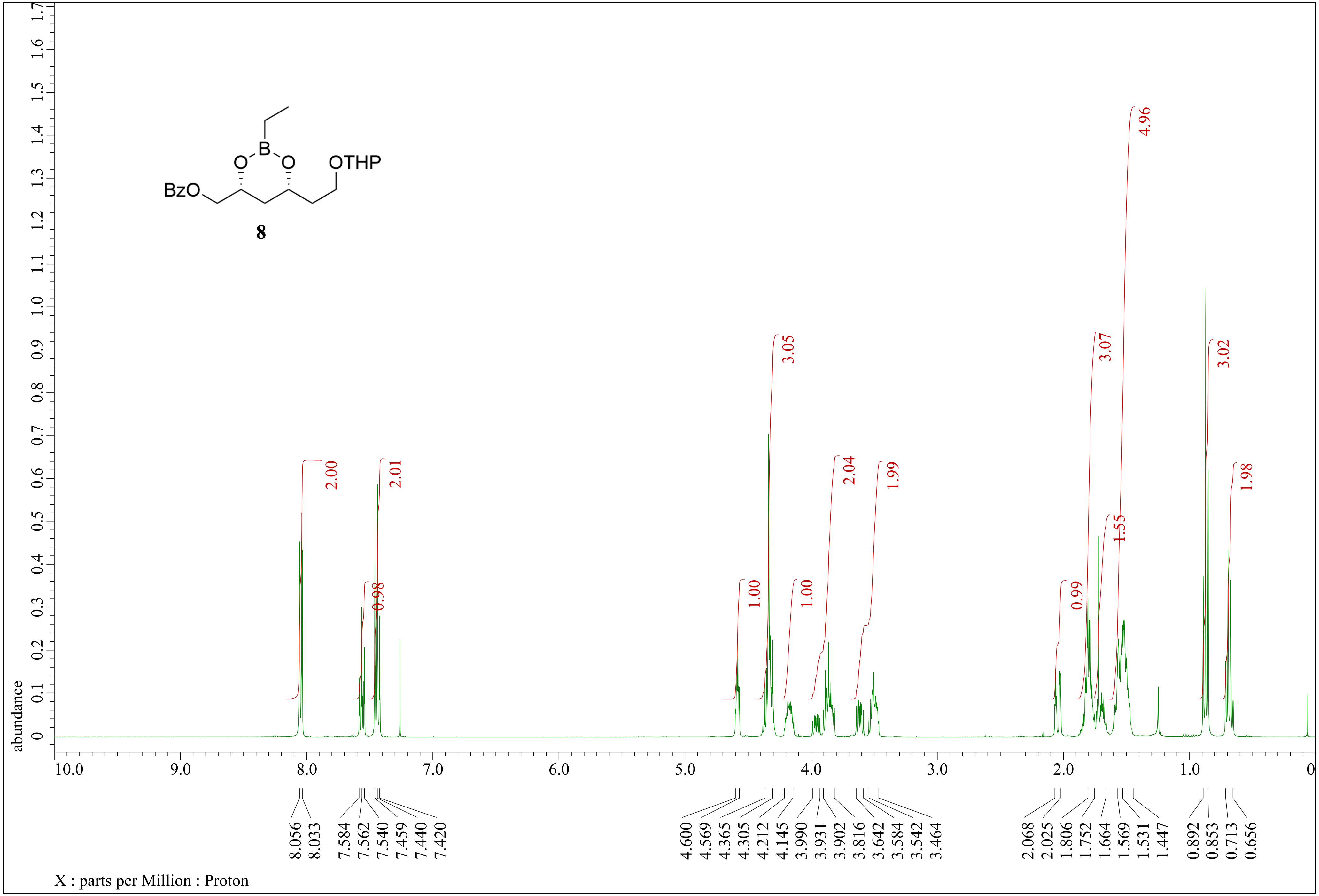

BzO
OTHP
B
O
8
abundance
X : parts per Million : Proton
2.00
0.98
2.01
1.00
3.05
1.00
2.04
1.99
0.99
3.07
1.55
4.96
3.02
1.98
8.056
8.033
7.584
7.562
7.540
7.459
7.440
7.420
4.600
4.569
4.365
4.305
4.212
4.145
3.990
3.931
3.902
3.816
3.642
3.584
3.542
3.464
2.068
2.025
1.806
1.752
1.664
1.569
1.531
1.447
0.892
0.853
0.713
0.656

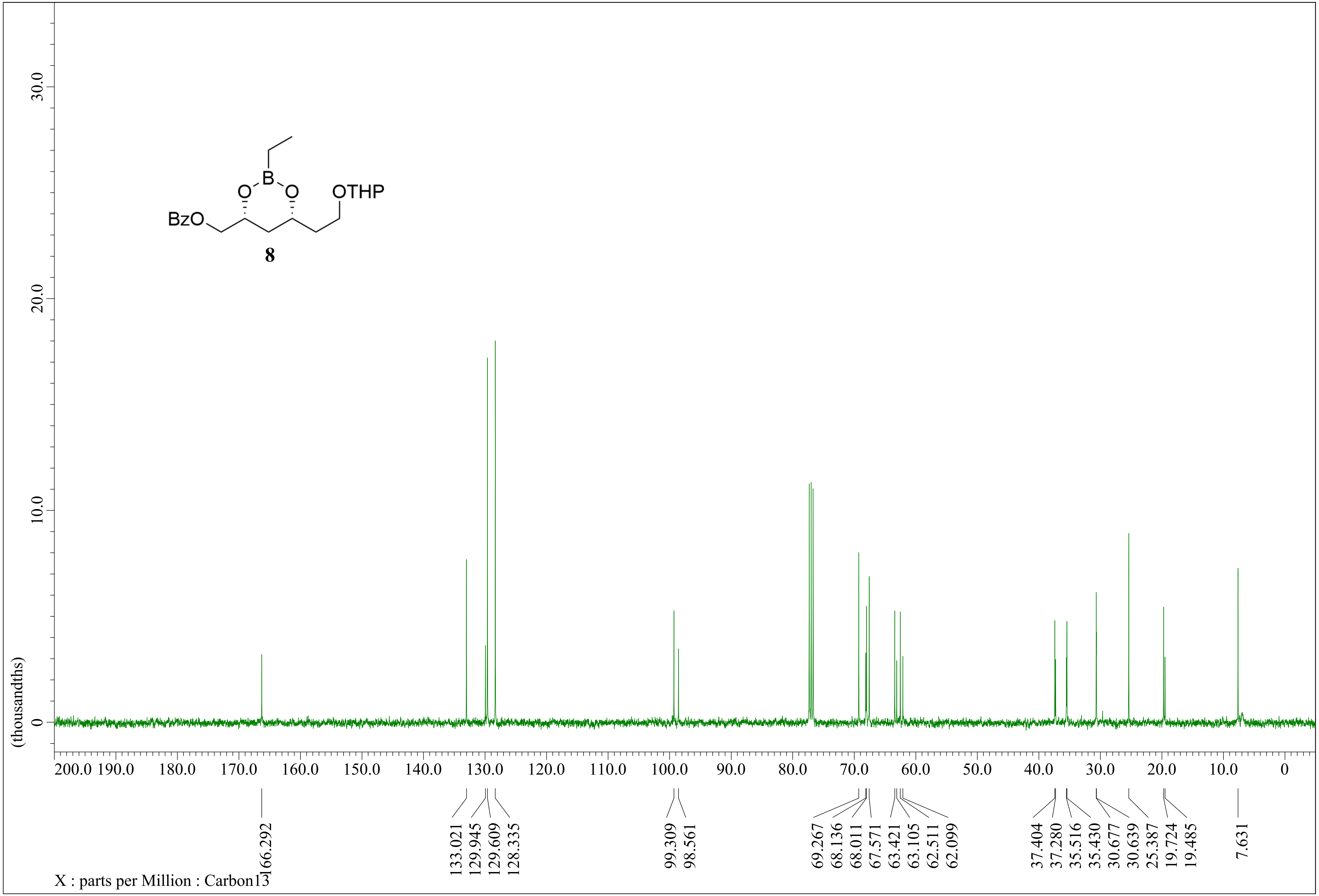

OTHP
BzO
B
O
O
8
30.0
20.0
10.0
0
(thousandths)
200.0 190.0 180.0 170.0 160.0 150.0 140.0 130.0 120.0 110.0 100.0 90.0 80.0 70.0 60.0 50.0 40.0 30.0 20.0 10.0 0
166.292
133.021
129.945
129.609
128.335
99.309
98.561
69.267
68.136
68.011
67.571
63.421
63.105
62.511
62.099
37.404
37.280
35.516
35.430
30.677
30.639
25.387
19.724
19.485
7.631
X : parts per Million : Carbon13

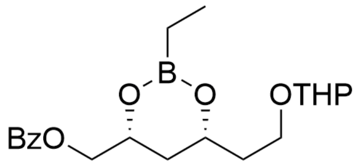

8

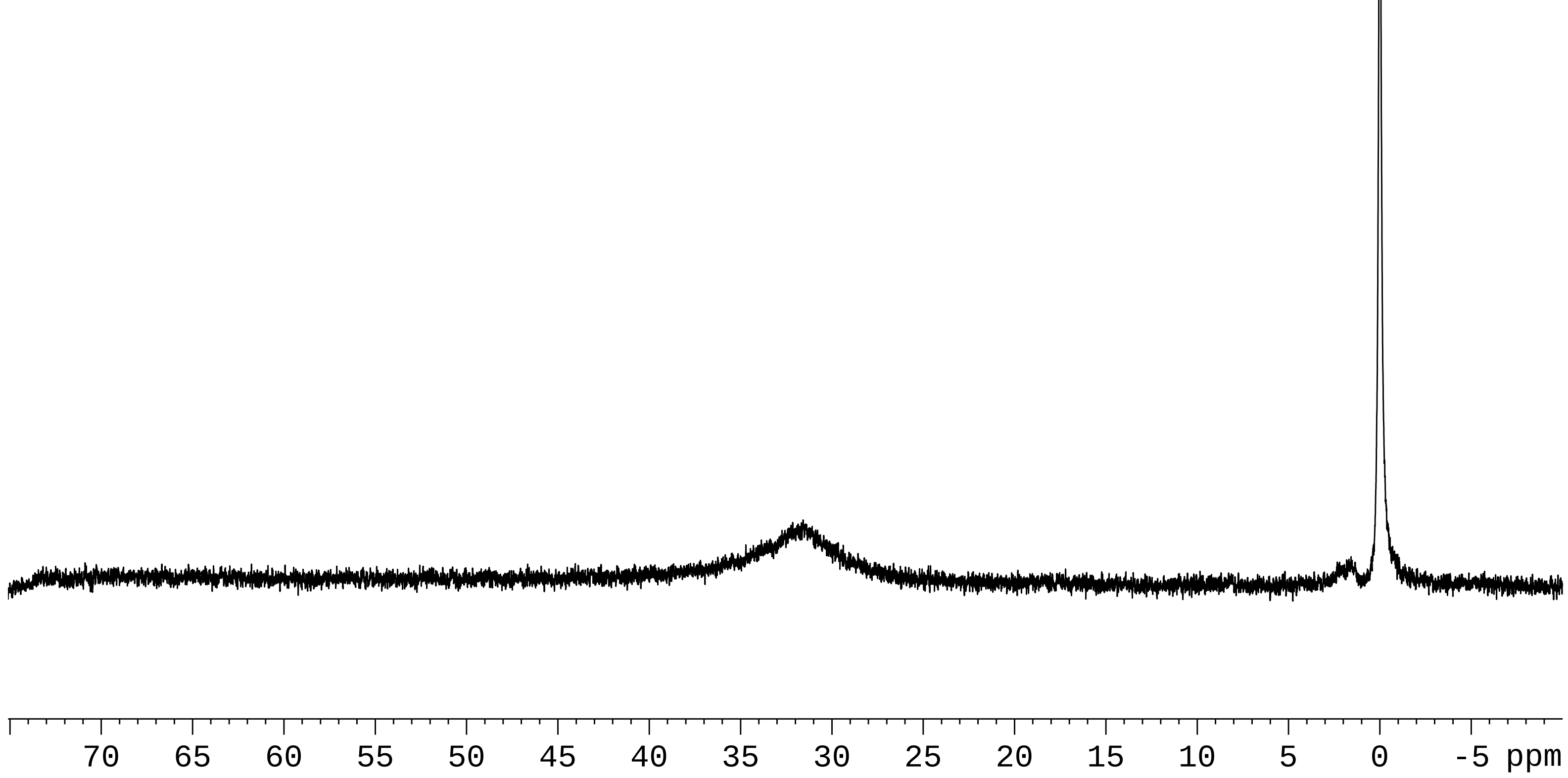


```
NAME       FJJ171207-2-B
EXPNO                  1
PROCNO                 1
Date_           20171207
Time               13.25
INSTRUM            spect
PROBHD   5 mm PABBO BB-
PULPROG               zg
TD                 65536
SOLVENT            CDCl3
NS                  2992
DS                     0
SWH            19230.770 Hz
FIDRES          0.293438 Hz
AQ             1.7039860 sec
RG                   203
DW                26.000 usec
DE                  6.50 usec
TE                 296.7 K
D1            1.00000000 sec
TD0                    1

======== CHANNEL f1 ========
NUC1                 11B
P1                  8.00 usec
PL1                -2.00 dB
SFO1         128.3904390 MHz
SI                 32768
SF           128.3904126 MHz
WDW                   EM
SSB                    0
LB                  1.00 Hz
GB                     0
PC                  1.40
```

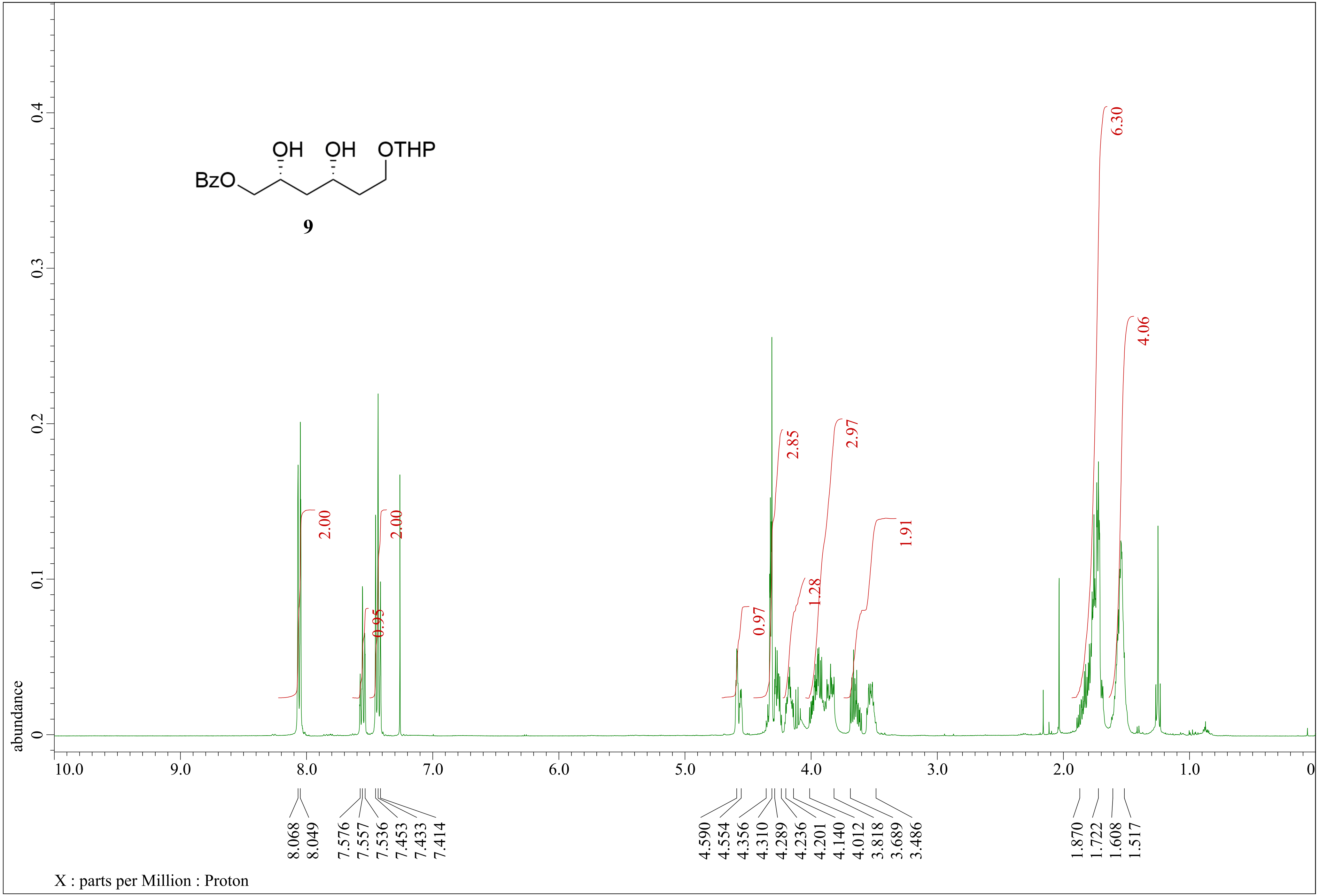
OH OH OTHP
BzO
9
abundance
0.4
0.3
0.2
0.1
0
2.00
2.00
0.95
0.97
2.85
1.28
2.97
1.91
6.30
4.06
10.0
9.0
8.0
7.0
6.0
5.0
4.0
3.0
2.0
1.0
0
8.068
8.049
7.576
7.557
7.536
7.453
7.433
7.414
4.590
4.554
4.356
4.310
4.289
4.236
4.201
4.140
4.012
3.818
3.689
3.486
1.870
1.722
1.608
1.517
X : parts per Million : Proton

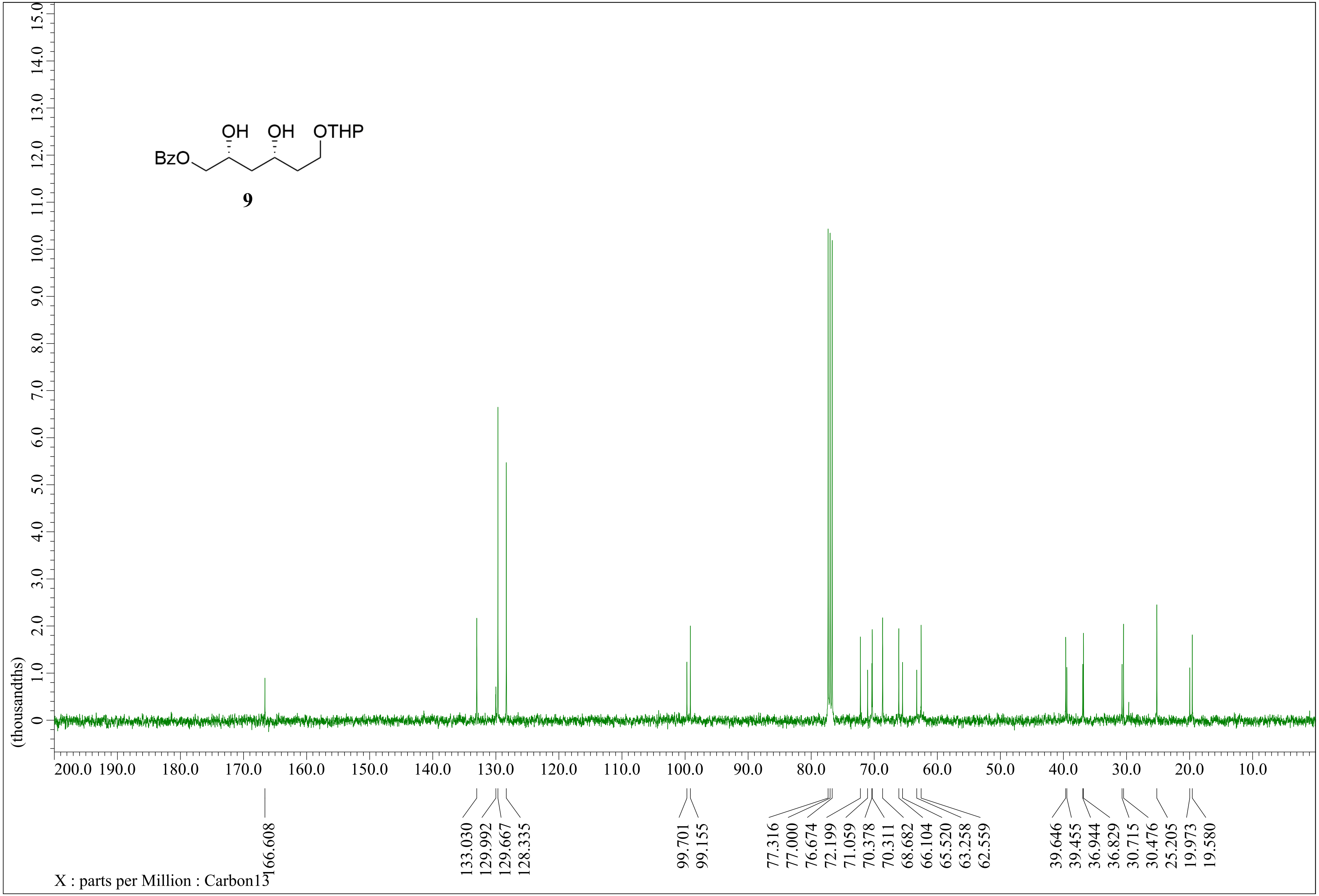

OH
OH
OTHP
BzO
9
(thousandths)
166.608
133.030
129.992
129.667
128.335
99.701
99.155
77.316
77.000
76.674
72.199
71.059
70.378
70.311
68.682
66.104
65.520
63.258
62.559
39.646
39.455
36.944
36.829
30.715
30.476
25.205
19.973
19.580
X : parts per Million : Carbon13

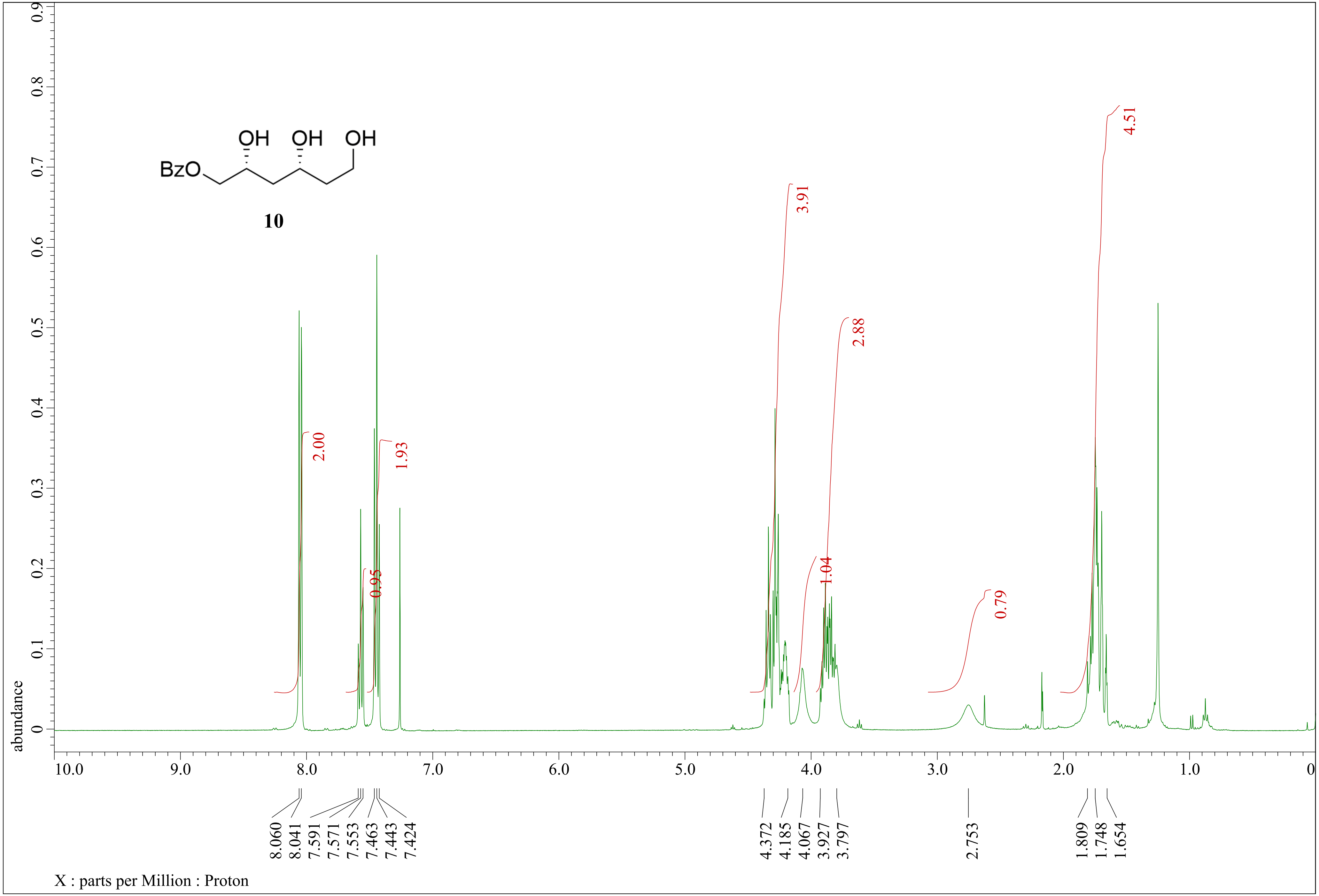
OH OH OH
BzO
10
2.00
1.93
0.95
3.91
2.88
1.04
0.79
4.51
abundance
8.060 8.041 7.591 7.571 7.553 7.463 7.443 7.424
4.372 4.185 4.067 3.927 3.797
2.753
1.809 1.748 1.654
X : parts per Million : Proton

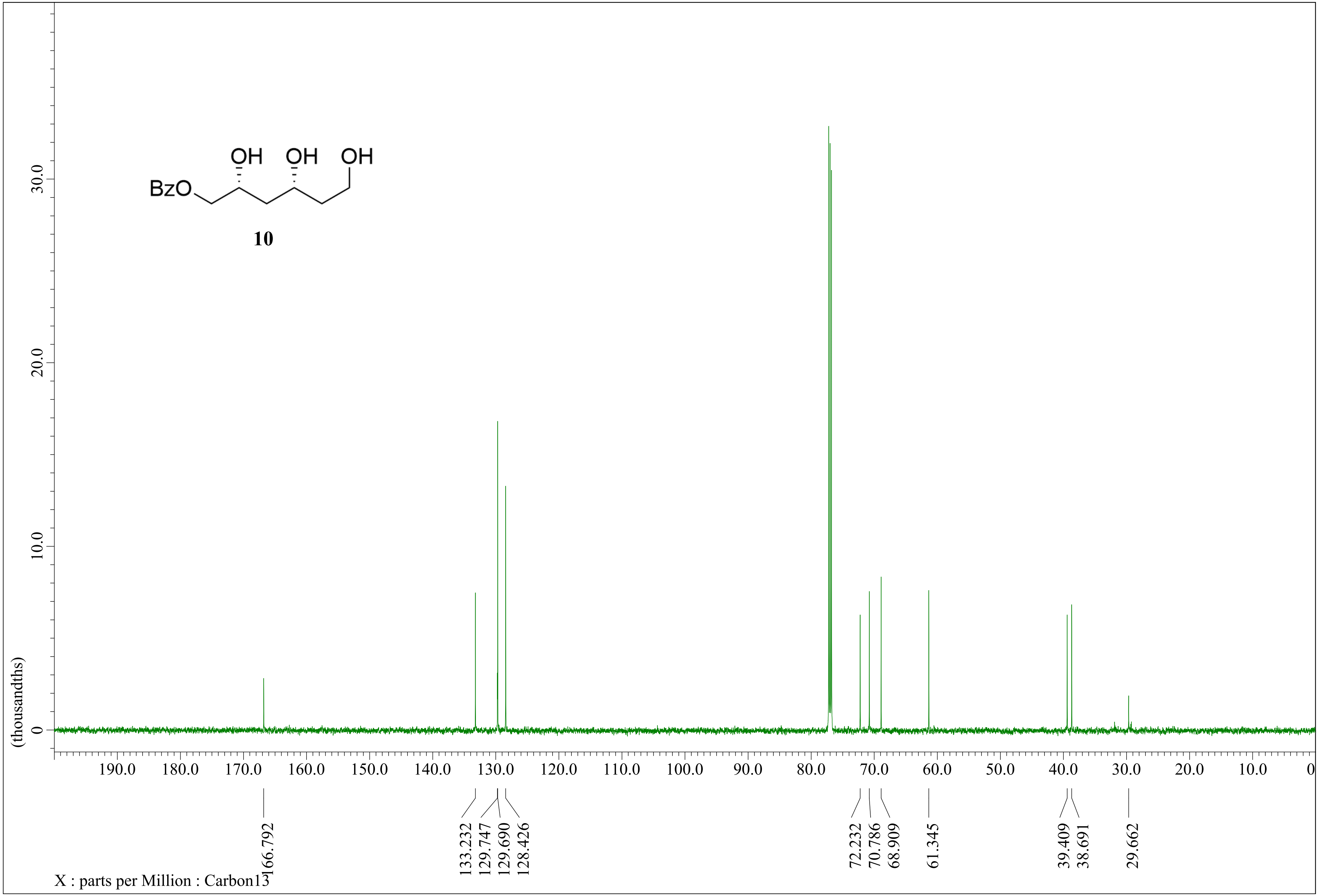
BzO
OH
OH
OH
10
30.0
20.0
10.0
0
(thousandths)
190.0
180.0
170.0
160.0
150.0
140.0
130.0
120.0
110.0
100.0
90.0
80.0
70.0
60.0
50.0
40.0
30.0
20.0
10.0
0
166.792
133.232
129.747
129.690
128.426
72.232
70.786
68.909
61.345
39.409
38.691
29.662
X : parts per Million : Carbon13

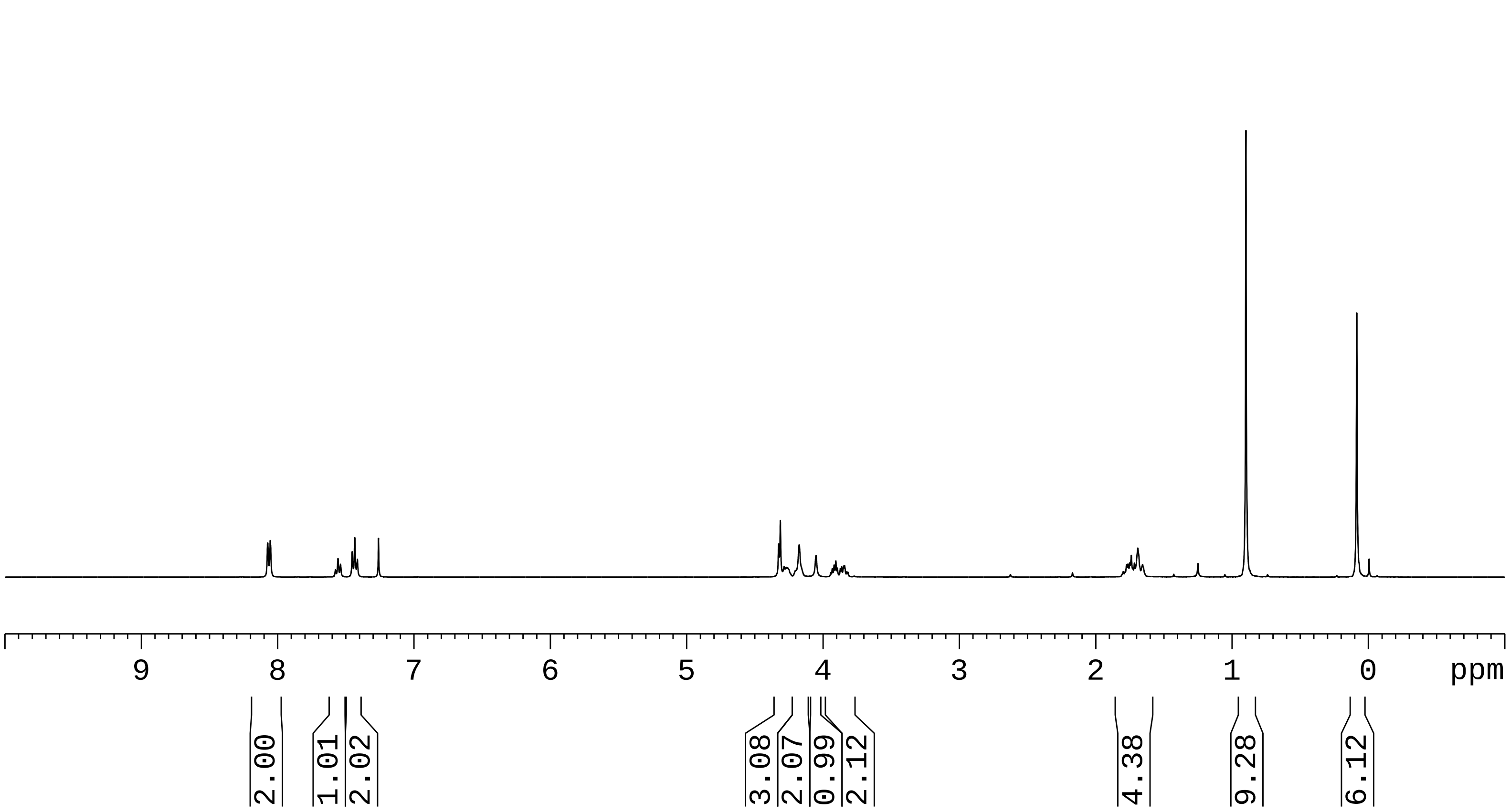

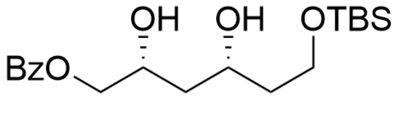

11

BRUKER

NAME	FJJ171121-1
EXPNO	1
PROCNO	1
Date_	20171122
Time	17.07
INSTRUM	spect
PROBHD	5 mm PABBO BB-
PULPROG	zg30
TD	65536
SOLVENT	CDCl3
NS	16
DS	2
SWH	8223.685 Hz
FIDRES	0.125483 Hz
AQ	3.9846387 sec
RG	90.5
DW	60.800 usec
DE	6.50 usec
TE	296.3 K
D1	1.00000000 sec
TD0	1

======== CHANNEL f1 ========

NUC1	1H
P1	15.50 usec
PL1	-1.00 dB
PL1W	13.18669796 W
SFO1	400.1724712 MHz
SI	32768
SF	400.1700028 MHz
WDW	EM
SSB	0
LB	0.30 Hz
GB	0
PC	1.00

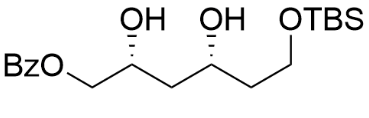

11

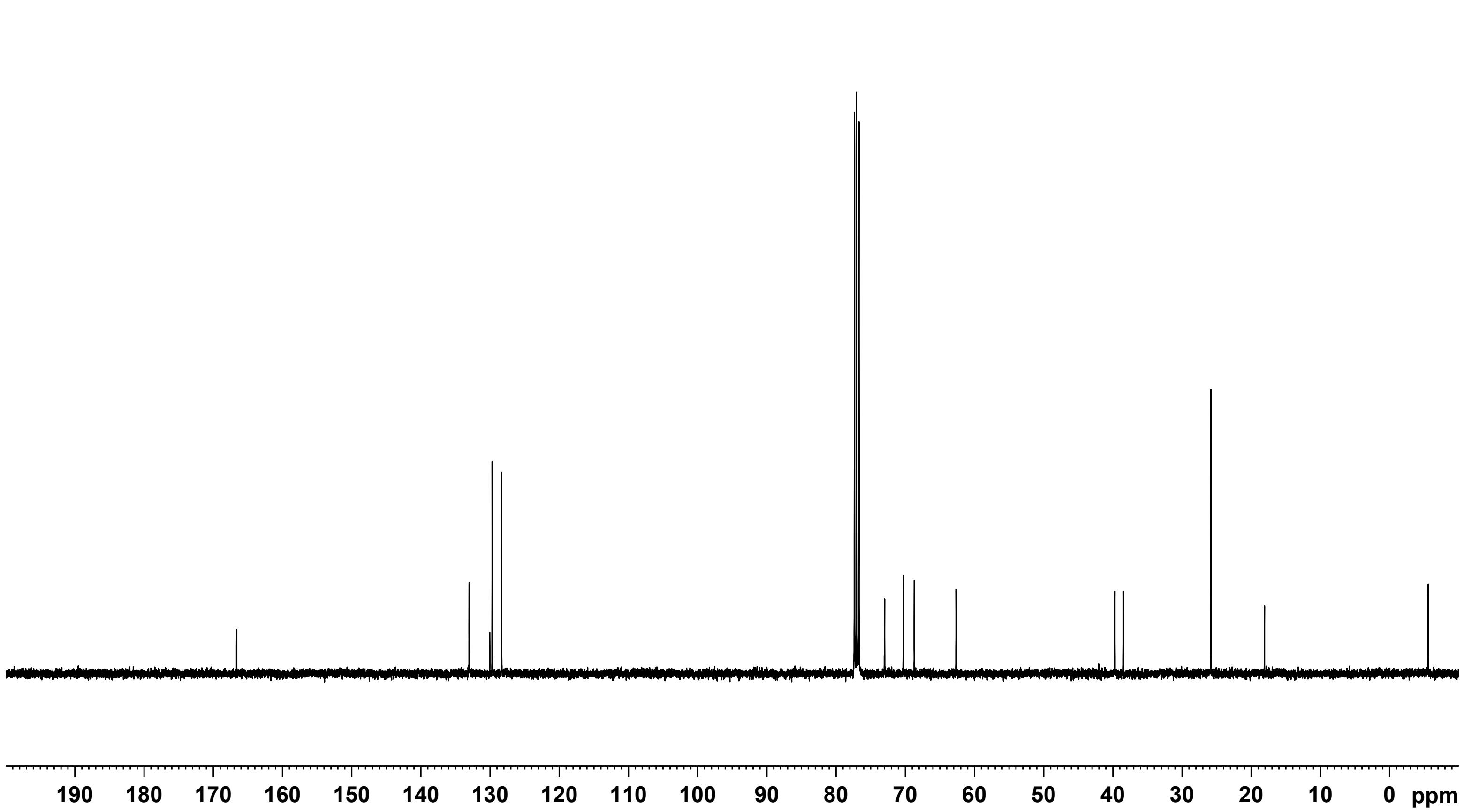

NAME	FJJ171121-1-C13
EXPNO	1
PROCNO	1
Date_	20171122
Time	17.24
INSTRUM	spect
PROBHD	5 mm PABBO BB-
PULPROG	zgpg30
TD	65536
SOLVENT	CDCl3
NS	205
DS	4
SWH	24038.461 Hz
FIDRES	0.366798 Hz
AQ	1.3631988 sec
RG	203
DW	20.800 usec
DE	6.50 usec
TE	296.5 K
D1	2.00000000 sec
D11	0.03000000 sec
TD0	1

======== CHANNEL f1 ========

NUC1	13C
P1	8.50 usec
PL1	-2.00 dB
PL1W	57.32743073 W
SFO1	100.6328888 MHz

======== CHANNEL f2 ========

CPDPRG2	waltz16
NUC2	1H
PCPD2	80.00 usec
PL2	-1.00 dB
PL12	13.26 dB
PL13	14.46 dB
PL2W	13.18669796 W
PL12W	0.49446553 W
PL13W	0.37509048 W
SFO2	400.1716007 MHz
SI	32768
SF	100.6228282 MHz
WDW	EM
SSB	0
LB	1.00 Hz
GB	0
PC	1.40

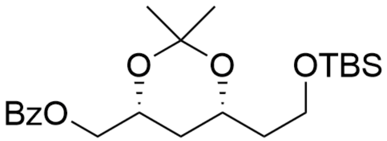

12

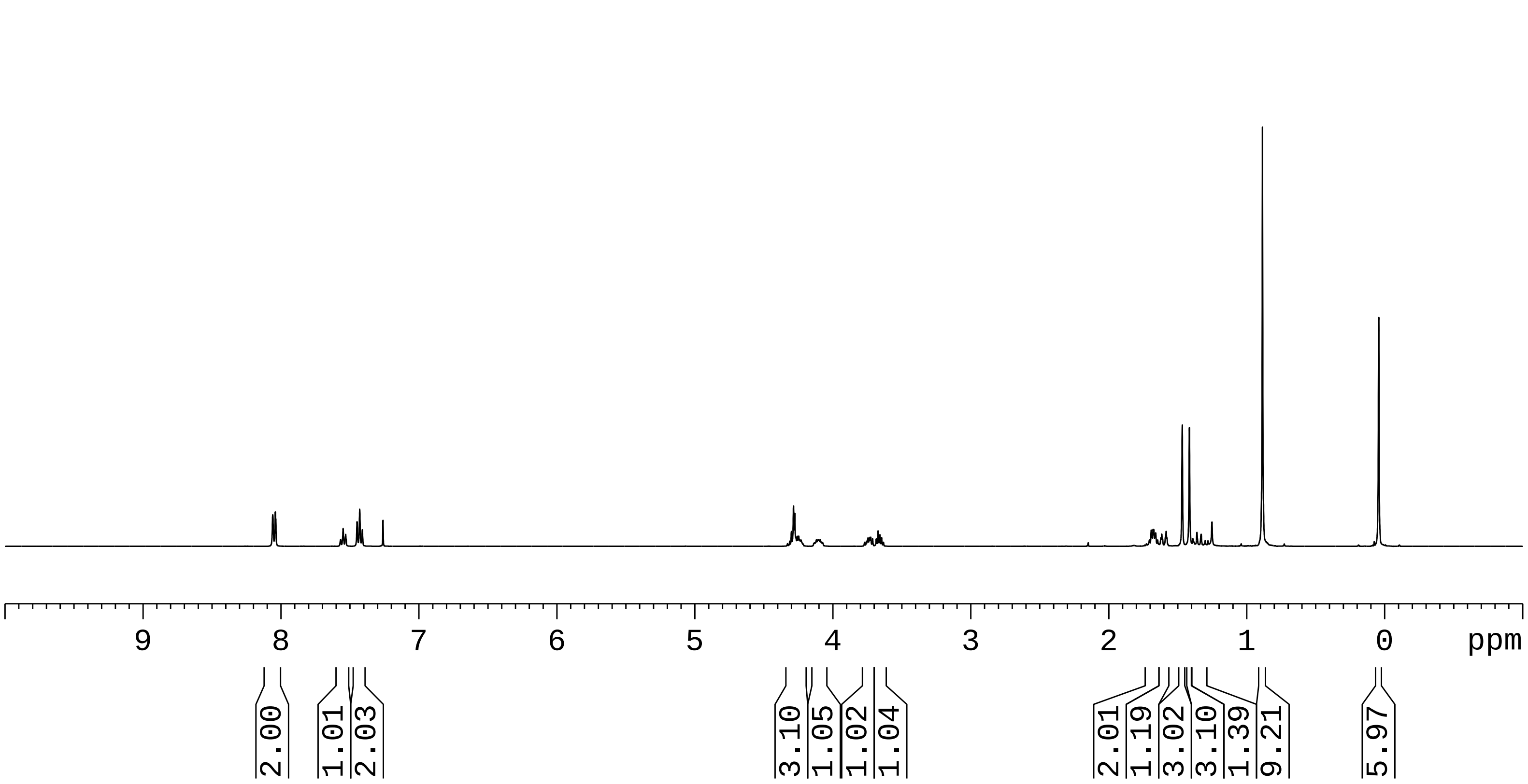

BRUKER

NAME	FJJ171113
EXPNO	1
PROCNO	1
Date_	20171113
Time	11.06
INSTRUM	spect
PROBHD	5 mm PABBO BB-
PULPROG	zg30
TD	65536
SOLVENT	CDCl3
NS	16
DS	2
SWH	8223.685 Hz
FIDRES	0.125483 Hz
AQ	3.9846387 sec
RG	32
DW	60.800 usec
DE	6.50 usec
TE	296.7 K
D1	1.00000000 sec
TD0	1

======== CHANNEL f1 ========

NUC1	1H
P1	15.50 usec
PL1	-1.00 dB
PL1W	13.18669796 W
SFO1	400.1724712 MHz
SI	32768
SF	400.1700028 MHz
WDW	EM
SSB	0
LB	0.30 Hz
GB	0
PC	1.00

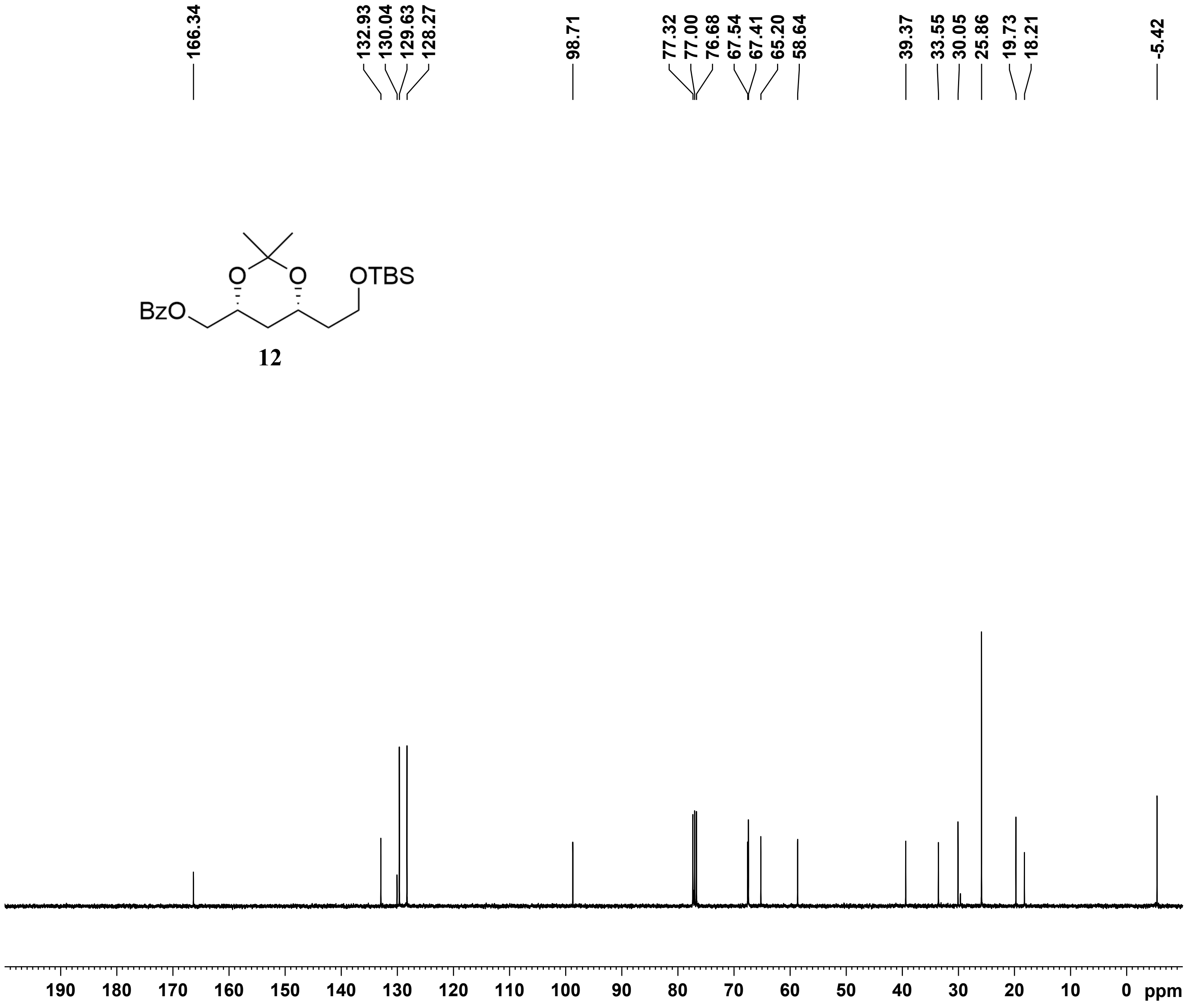

12

NAME	FJJ171113-C13	
EXPNO	1	
PROCNO	1	
Date_	20171113	
Time	11.13	
INSTRUM	spect	
PROBHD	5 mm PABBO BB-	
PULPROG	zgpg30	
TD	65536	
SOLVENT	CDCl3	
NS	57	
DS	4	
SWH	24038.461	Hz
FIDRES	0.366798	Hz
AQ	1.3631988	sec
RG	203	
DW	20.800	usec
DE	6.50	usec
TE	297.2	K
D1	2.00000000	sec
D11	0.03000000	sec
TD0	1	
======== CHANNEL f1 ========		
NUC1	13C	
P1	8.50	usec
PL1	-2.00	dB
PL1W	57.32743073	W
SFO1	100.6328888	MHz
======== CHANNEL f2 ========		
CPDPRG2	waltz16	
NUC2	1H	
PCPD2	80.00	usec
PL2	-1.00	dB
PL12	13.26	dB
PL13	14.46	dB
PL2W	13.18669796	W
PL12W	0.49446553	W
PL13W	0.37509048	W
SFO2	400.1716007	MHz
SI	32768	
SF	100.6228318	MHz
WDW	EM	
SSB	0	
LB	1.00	Hz
GB	0	
PC	1.40	

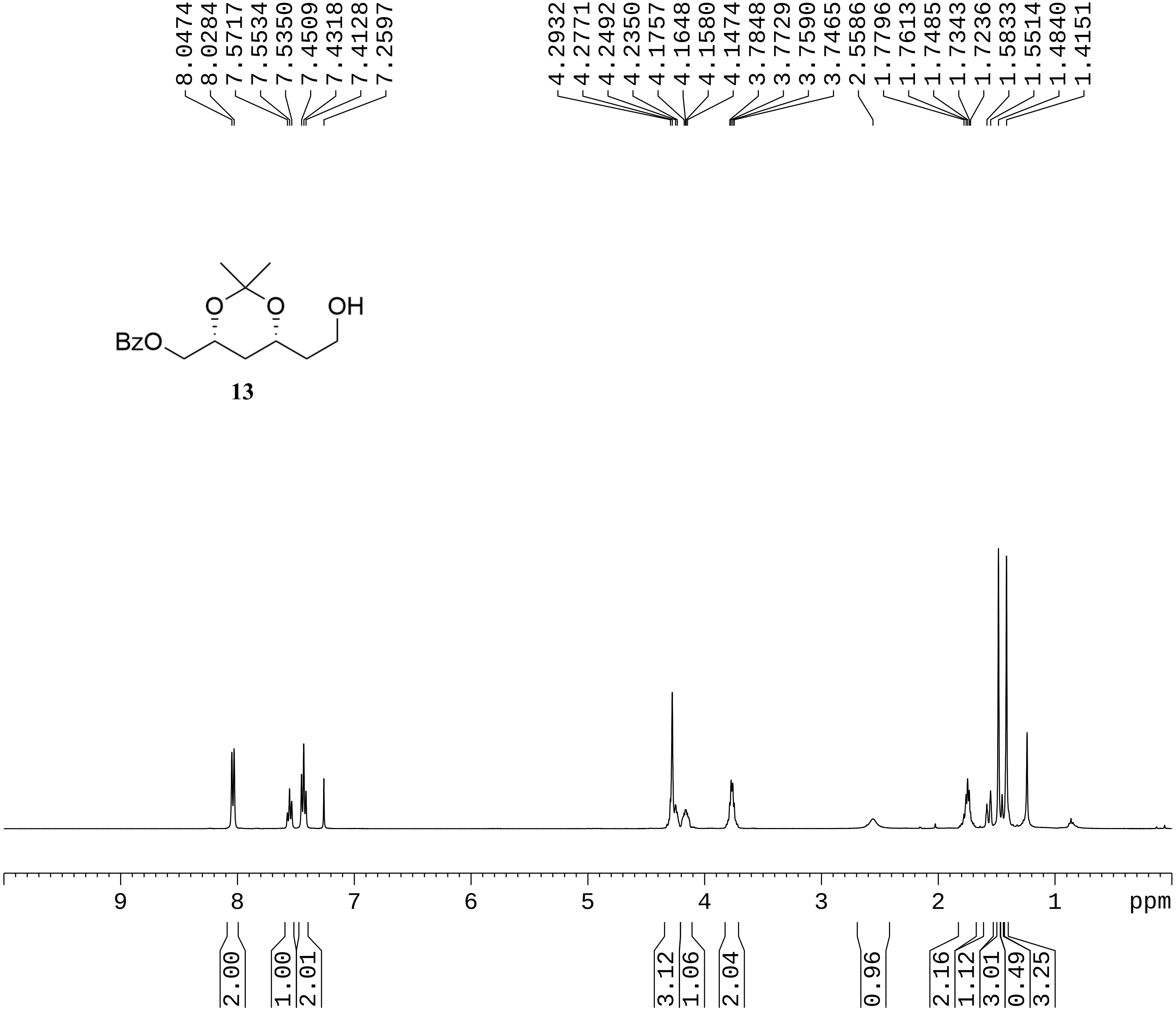


```
NAME          FJJ171113-1
EXPNO                   1
PROCNO                  1
Date_            20171114
Time                 9.43
INSTRUM             spect
PROBHD     5 mm PABBO BB-
PULPROG              zg30
TD                  65536
SOLVENT             CDCl3
NS                     16
DS                      2
SWH              8223.685 Hz
FIDRES           0.125483 Hz
AQ              3.9846387 sec
RG                     57
DW                 60.800 usec
DE                   6.50 usec
TE                  295.1 K
D1             1.00000000 sec
TD0                     1

======== CHANNEL f1 ========
NUC1                   1H
P1                  15.50 usec
PL1                 -1.00 dB
PL1W          13.18669796 W
SFO1          400.1724712 MHz
SI                  32768
SF            400.1700028 MHz
WDW                    EM
SSB                     0
LB                   0.30 Hz
GB                      0
PC                   1.00
```

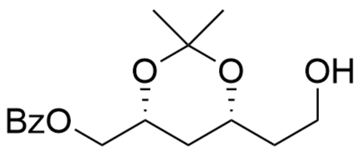

13

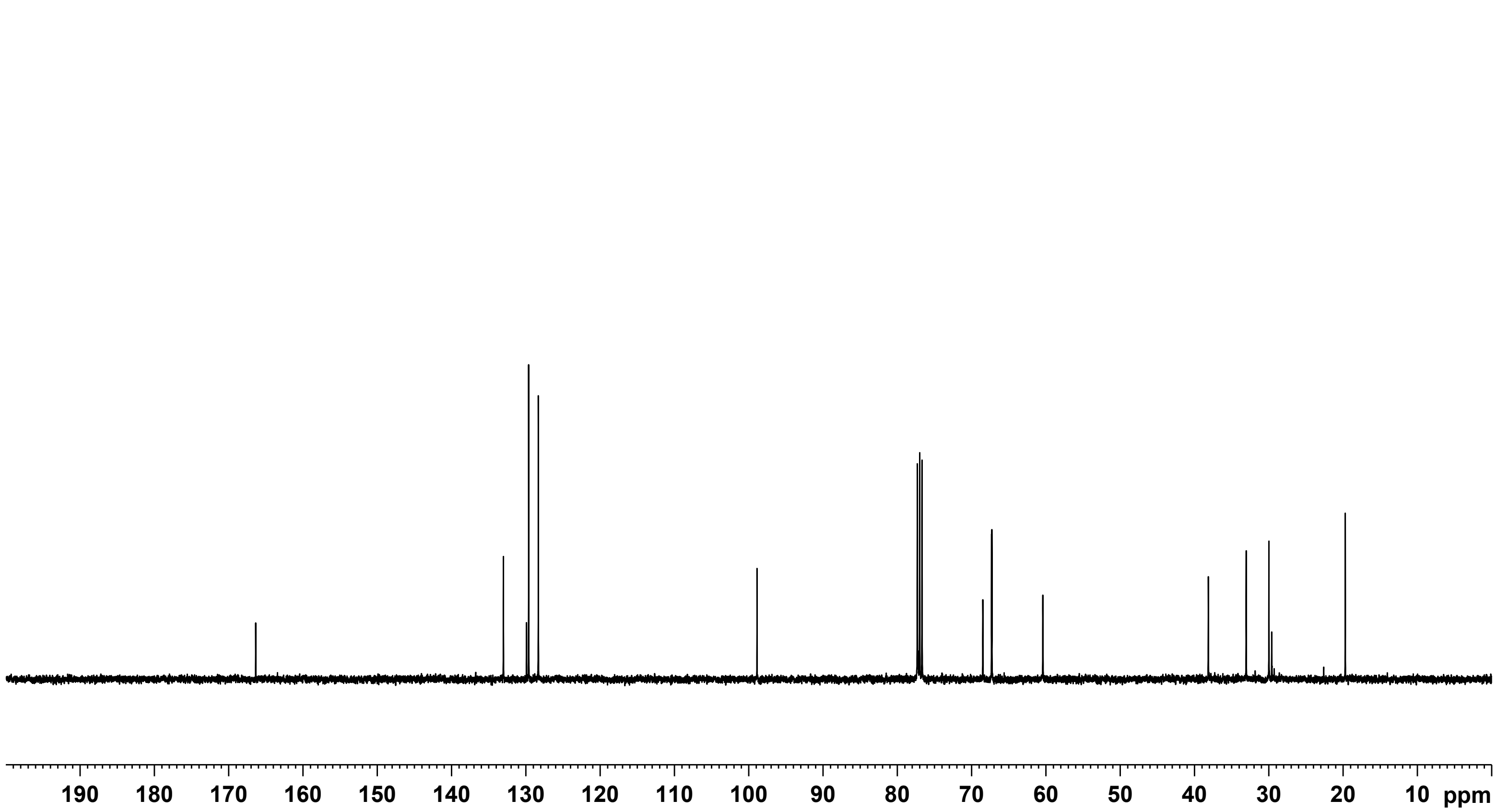

BRUKER

NAME	FJJ171113-1-C13
EXPNO	1
PROCNO	1
Date_	20171114
Time	9.53
INSTRUM	spect
PROBHD	5 mm PABBO BB-
PULPROG	zgpg30
TD	65536
SOLVENT	CDCl3
NS	76
DS	4
SWH	24038.461 Hz
FIDRES	0.366798 Hz
AQ	1.3631988 sec
RG	203
DW	20.800 usec
DE	6.50 usec
TE	296.2 K
D1	2.00000000 sec
D11	0.03000000 sec
TD0	1

======== CHANNEL f1 ========

NUC1	13C
P1	8.50 usec
PL1	-2.00 dB
PL1W	57.32743073 W
SFO1	100.6328888 MHz

======== CHANNEL f2 ========

CPDPRG2	waltz16
NUC2	1H
PCPD2	80.00 usec
PL2	-1.00 dB
PL12	13.26 dB
PL13	14.46 dB
PL2W	13.18669796 W
PL12W	0.49446553 W
PL13W	0.37509048 W
SFO2	400.1716007 MHz
SI	32768
SF	100.6228333 MHz
WDW	EM
SSB	0
LB	1.00 Hz
GB	0
PC	1.40

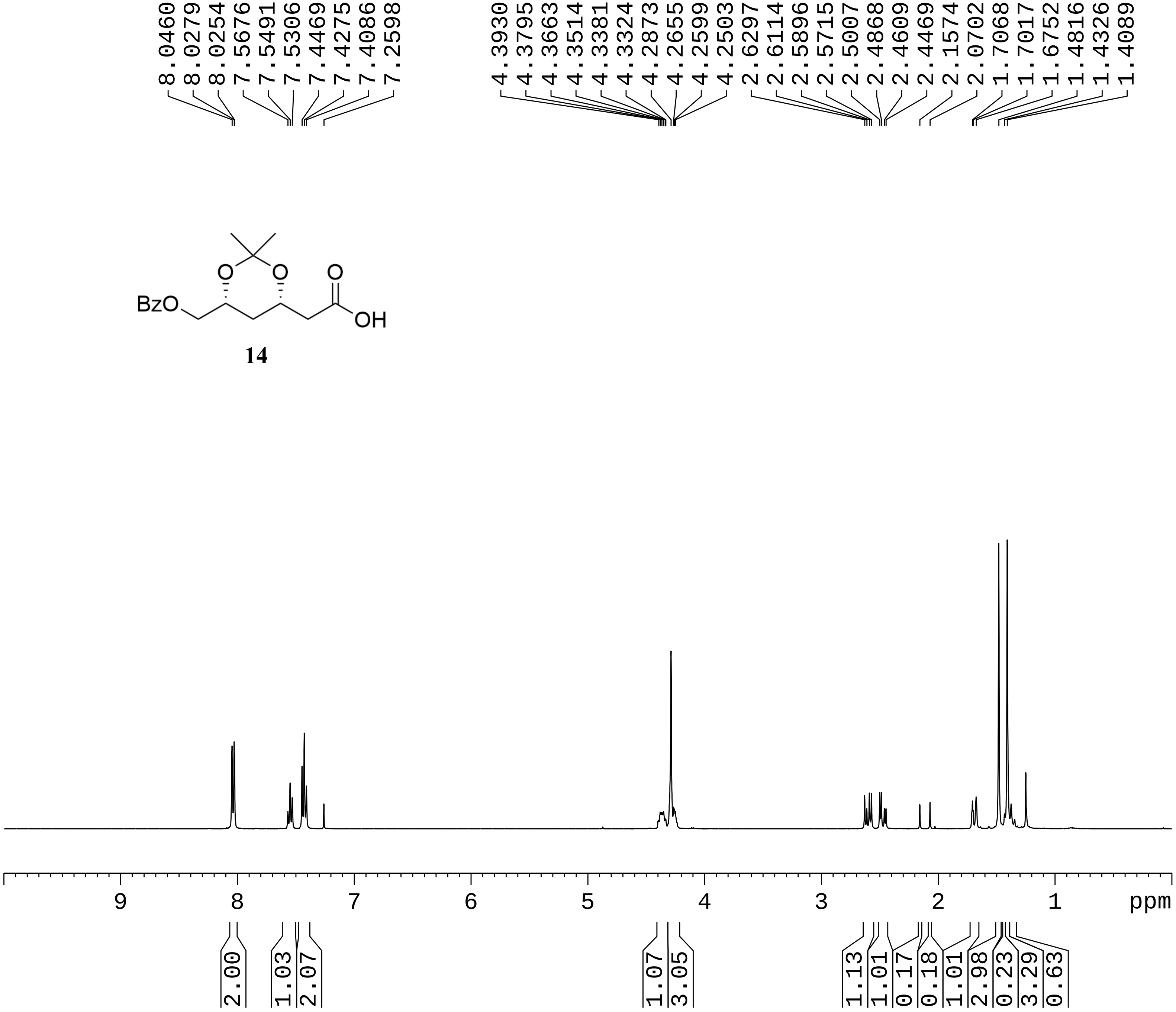

14

BRUKER

NAME	fjj171114
EXPNO	1
PROCNO	1
Date_	20171114
Time	19.37
INSTRUM	spect
PROBHD	5 mm PABBO BB-
PULPROG	zg30
TD	65536
SOLVENT	CDCl3
NS	16
DS	2
SWH	8223.685 Hz
FIDRES	0.125483 Hz
AQ	3.9846387 sec
RG	36
DW	60.800 usec
DE	6.50 usec
TE	295.6 K
D1	1.00000000 sec
TD0	1

======== CHANNEL f1 ========

NUC1	1H
P1	15.50 usec
PL1	-1.00 dB
PL1W	13.18669796 W
SFO1	400.1724712 MHz
SI	32768
SF	400.1700030 MHz
WDW	EM
SSB	0
LB	0.30 Hz
GB	0
PC	1.00

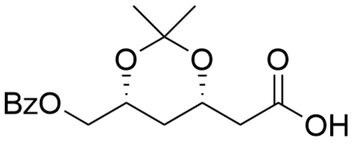

14

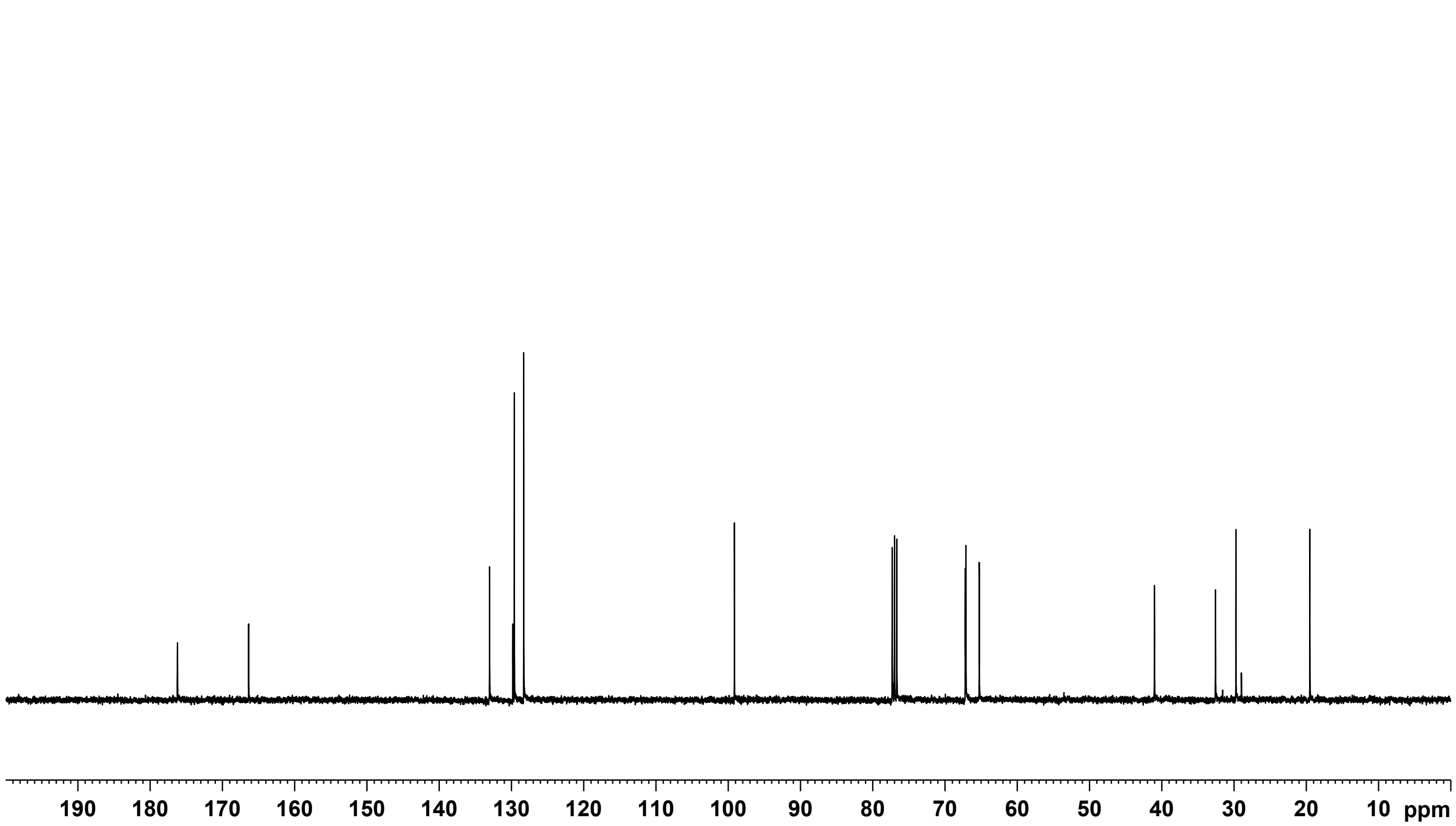


```
NAME          fjj171114-C13
EXPNO                     1
PROCNO                    1
Date_              20171114
Time                  19.48
INSTRUM               spect
PROBHD   5 mm PABBO BB-
PULPROG             zgpg30
TD                    65536
SOLVENT               CDCl3
NS                       42
DS                        4
SWH           24038.461 Hz
FIDRES         0.366798 Hz
AQ            1.3631988 sec
RG                      203
DW               20.800 usec
DE                 6.50 usec
TE                296.1 K
D1           2.00000000 sec
D11          0.03000000 sec
TD0                       1

======== CHANNEL f1 ========
NUC1                    13C
P1                 8.50 usec
PL1               -2.00 dB
PL1W        57.32743073 W
SFO1        100.6328888 MHz

======== CHANNEL f2 ========
CPDPRG2             waltz16
NUC2                     1H
PCPD2             80.00 usec
PL2               -1.00 dB
PL12              13.26 dB
PL13              14.46 dB
PL2W        13.18669796 W
PL12W        0.49446553 W
PL13W        0.37509048 W
SFO2        400.1716007 MHz
SI                    32768
SF          100.6228370 MHz
WDW                      EM
SSB                       0
LB                 1.00 Hz
GB                        0
PC                     1.40
```

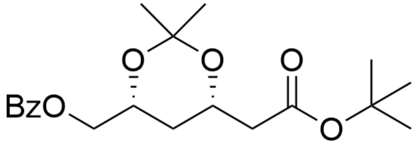

15

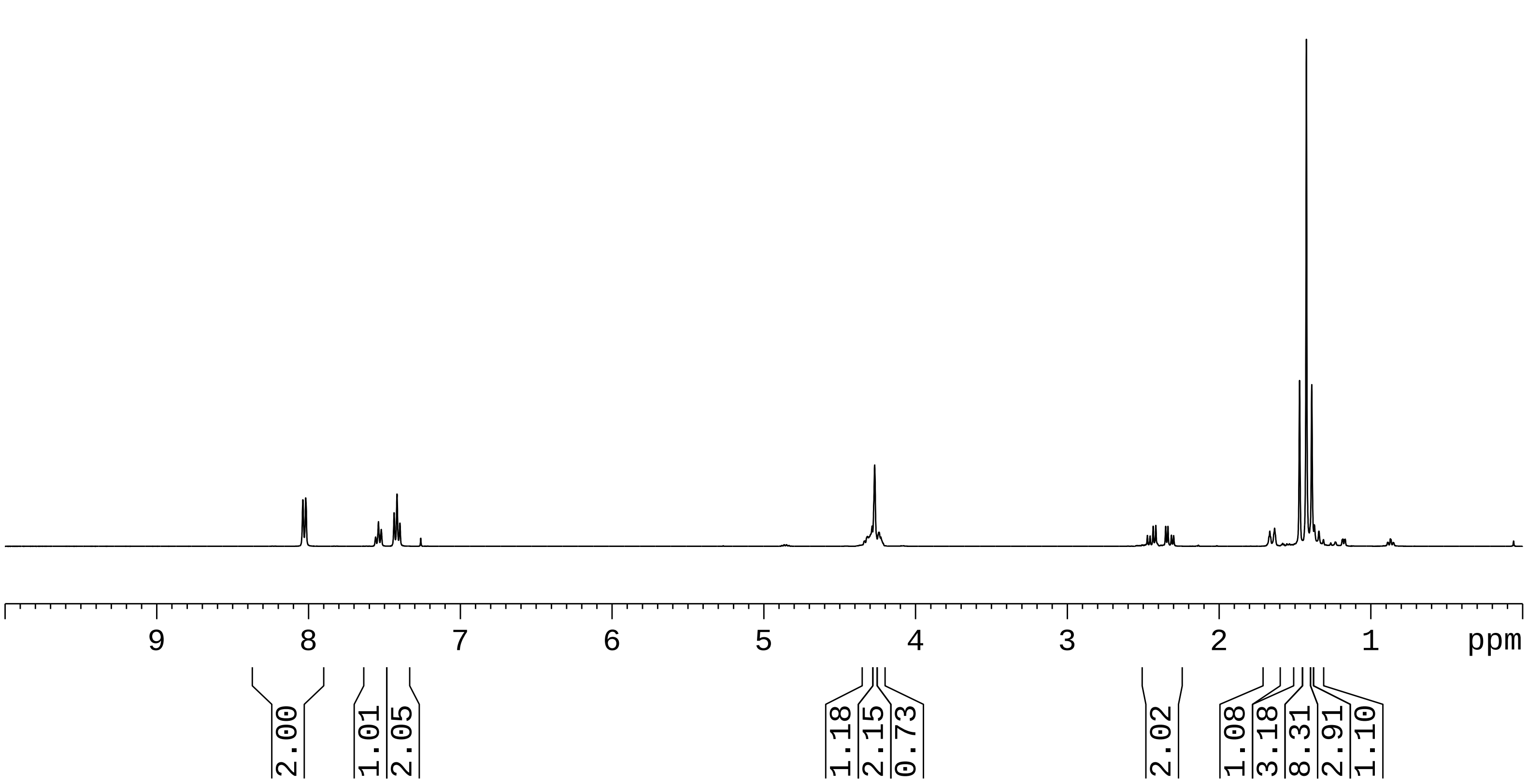


```
NAME            FJJ171115
EXPNO                   1
PROCNO                  1
Date_            20171115
Time                20.40
INSTRUM             spect
PROBHD     5 mm PABBO BB-
PULPROG              zg30
TD                  65536
SOLVENT             CDCl3
NS                     16
DS                      2
SWH              8223.685 Hz
FIDRES           0.125483 Hz
AQ              3.9846387 sec
RG                     36
DW                 60.800 usec
DE                   6.50 usec
TE                  295.6 K
D1             1.00000000 sec
TD0                     1

======== CHANNEL f1 ========
NUC1                   1H
P1                  15.50 usec
PL1                 -1.00 dB
PL1W          13.18669796 W
SFO1          400.1724712 MHz
SI                  32768
SF            400.1700025 MHz
WDW                    EM
SSB                     0
LB                   0.30 Hz
GB                      0
PC                   1.00
```

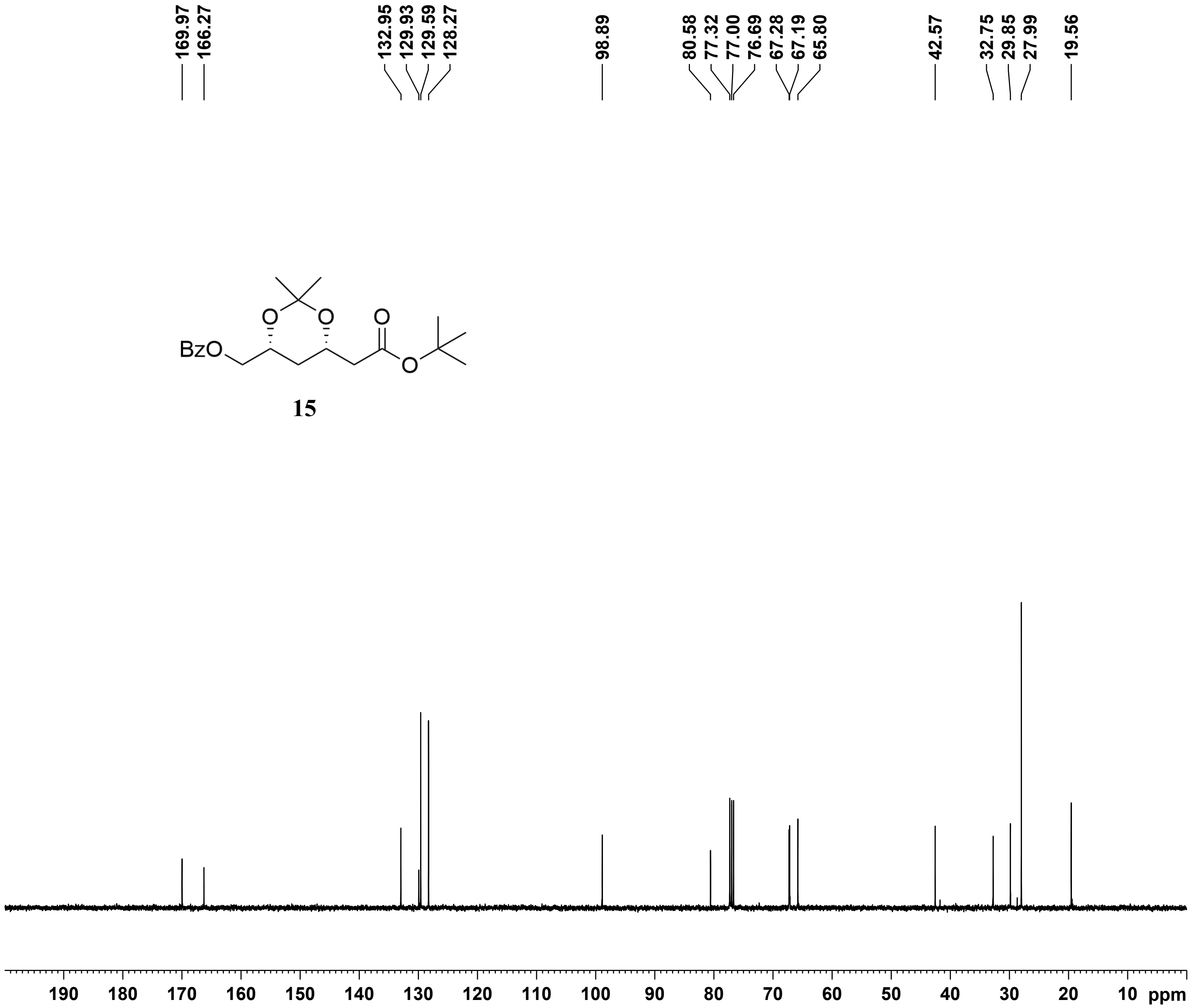

NAME	FJJ171115-C13
EXPNO	1
PROCNO	1
Date_	20171115
Time	20.48
INSTRUM	spect
PROBHD	5 mm PABBO BB-
PULPROG	zgpg30
TD	65536
SOLVENT	CDCl3
NS	36
DS	4
SWH	24038.461 Hz
FIDRES	0.366798 Hz
AQ	1.3631988 sec
RG	203
DW	20.800 usec
DE	6.50 usec
TE	296.1 K
D1	2.00000000 sec
D11	0.03000000 sec
TD0	1

======== CHANNEL f1 ========

NUC1	13C
P1	8.50 usec
PL1	-2.00 dB
PL1W	57.32743073 W
SFO1	100.6328888 MHz

======== CHANNEL f2 ========

CPDPRG2	waltz16
NUC2	1H
PCPD2	80.00 usec
PL2	-1.00 dB
PL12	13.26 dB
PL13	14.46 dB
PL2W	13.18669796 W
PL12W	0.49446553 W
PL13W	0.37509048 W
SFO2	400.1716007 MHz
SI	32768
SF	100.6228341 MHz
WDW	EM
SSB	0
LB	1.00 Hz
GB	0
PC	1.40

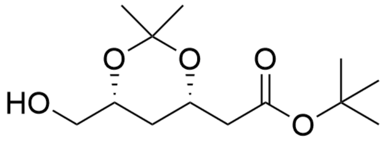

16

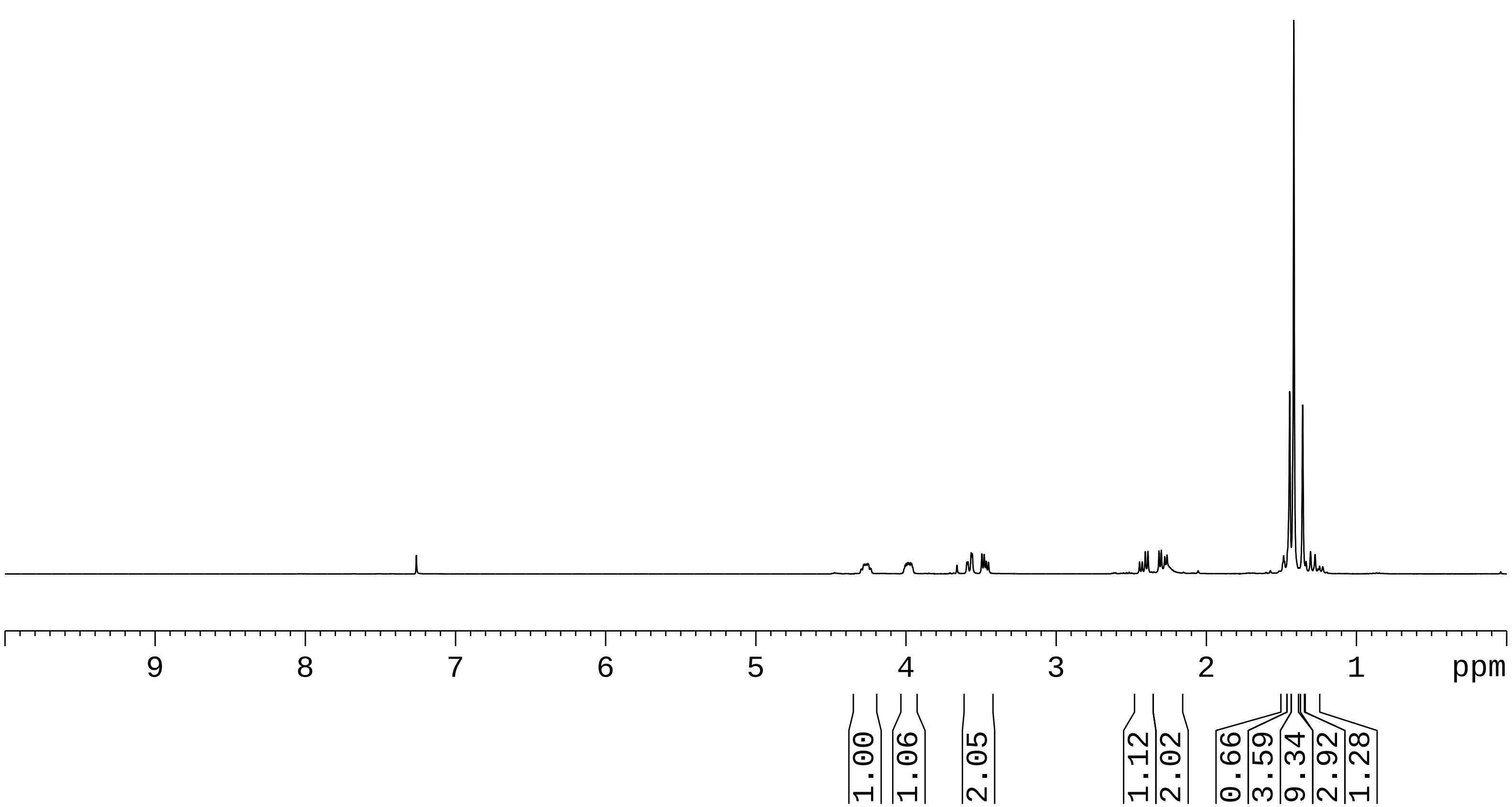

BRUKER

NAME	FJJ171116-1
EXPNO	1
PROCNO	1
Date_	20171118
Time	9.41
INSTRUM	spect
PROBHD	5 mm PABBO BB-
PULPROG	zg30
TD	65536
SOLVENT	CDCl3
NS	16
DS	2
SWH	8223.685 Hz
FIDRES	0.125483 Hz
AQ	3.9846387 sec
RG	64
DW	60.800 usec
DE	6.50 usec
TE	292.8 K
D1	1.00000000 sec
TD0	1

======== CHANNEL f1 ========

NUC1	1H
P1	15.50 usec
PL1	-1.00 dB
PL1W	13.18669796 W
SFO1	400.1724712 MHz
SI	32768
SF	400.1700028 MHz
WDW	EM
SSB	0
LB	0.30 Hz
GB	0
PC	1.00

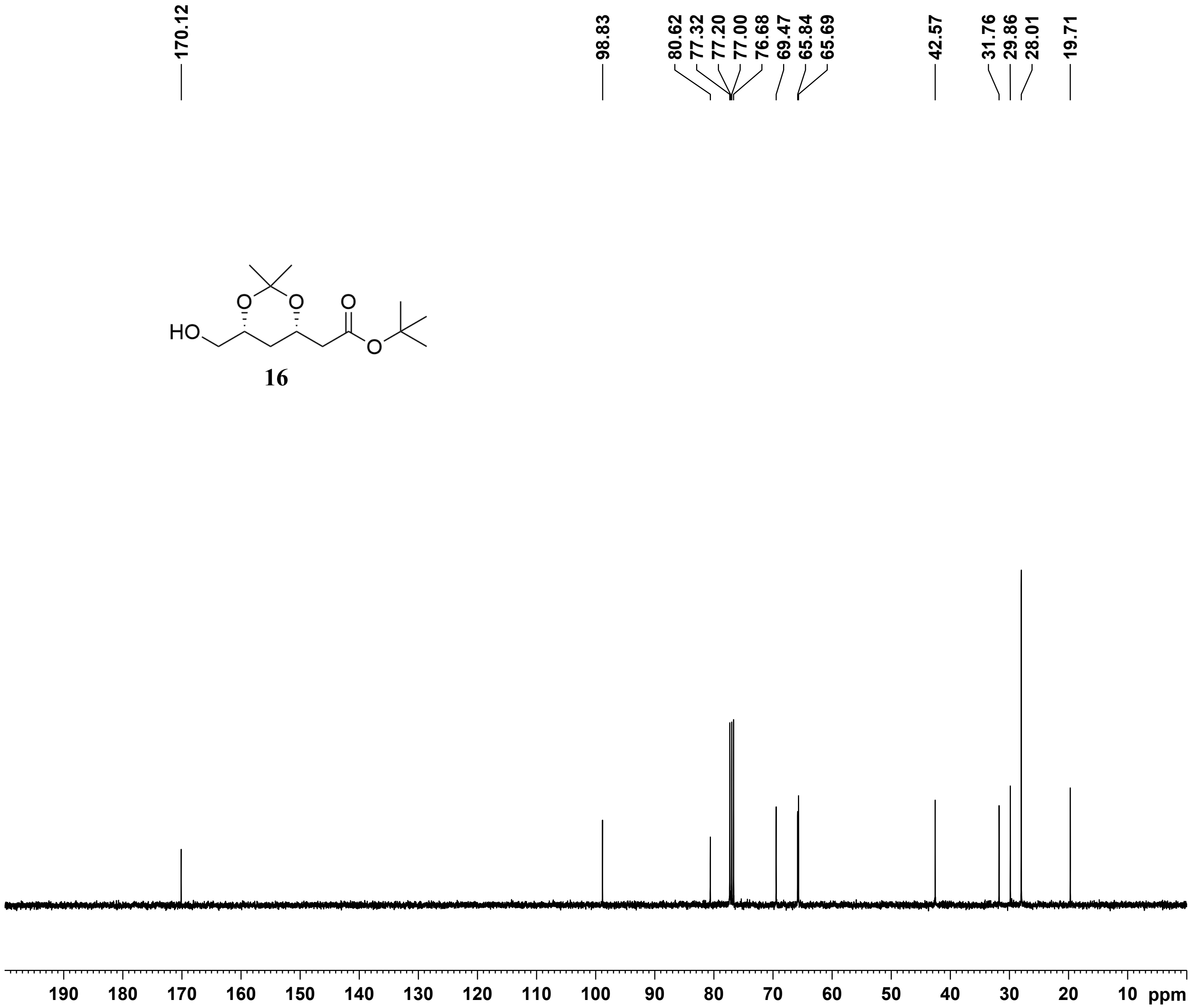

16

NAME	FJJ171116-1-C13	
EXPNO	1	
PROCNO	1	
Date_	20171118	
Time	9.47	
INSTRUM	spect	
PROBHD	5 mm PABBO BB-	
PULPROG	zgpg30	
TD	65536	
SOLVENT	CDCl3	
NS	56	
DS	4	
SWH	24038.461	Hz
FIDRES	0.366798	Hz
AQ	1.3631988	sec
RG	203	
DW	20.800	usec
DE	6.50	usec
TE	293.7	K
D1	2.00000000	sec
D11	0.03000000	sec
TD0	1	
======== CHANNEL f1 ========		
NUC1	13C	
P1	8.50	usec
PL1	-2.00	dB
PL1W	57.32743073	W
SFO1	100.6328888	MHz
======== CHANNEL f2 ========		
CPDPRG2	waltz16	
NUC2	1H	
PCPD2	80.00	usec
PL2	-1.00	dB
PL12	14.26	dB
PL13	14.46	dB
PL2W	13.18669796	W
PL12W	0.39276794	W
PL13W	0.37509048	W
SFO2	400.1716007	MHz
SI	32768	
SF	100.6228318	MHz
WDW	EM	
SSB	0	
LB	1.00	Hz
GB	0	
PC	1.40	

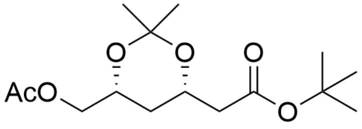

17

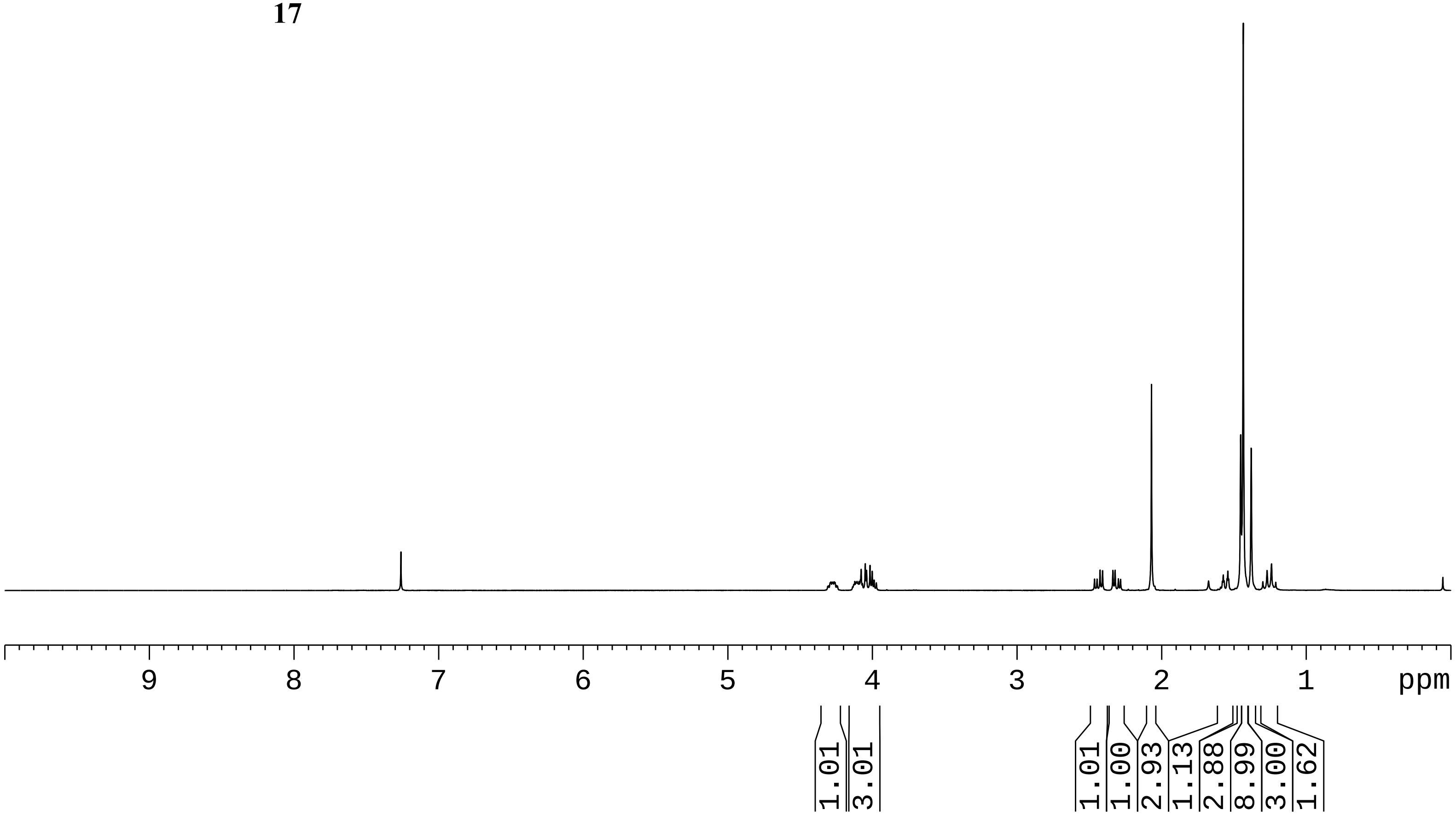

BRUKER

NAME	FJJ171218	
EXPNO	1	
PROCNO	1	
Date_	20171218	
Time	14.46	
INSTRUM	spect	
PROBHD	5 mm PABBO BB-	
PULPROG	zg30	
TD	65536	
SOLVENT	CDCl3	
NS	16	
DS	2	
SWH	8223.685	Hz
FIDRES	0.125483	Hz
AQ	3.9846387	sec
RG	80.6	
DW	60.800	usec
DE	6.50	usec
TE	294.6	K
D1	1.00000000	sec
TD0	1	

======== CHANNEL f1 ========

NUC1	1H	
P1	15.50	usec
PL1	-1.00	dB
PL1W	13.18669796	W
SFO1	400.1724712	MHz
SI	32768	
SF	400.1700028	MHz
WDW	EM	
SSB	0	
LB	0.30	Hz
GB	0	
PC	1.00	

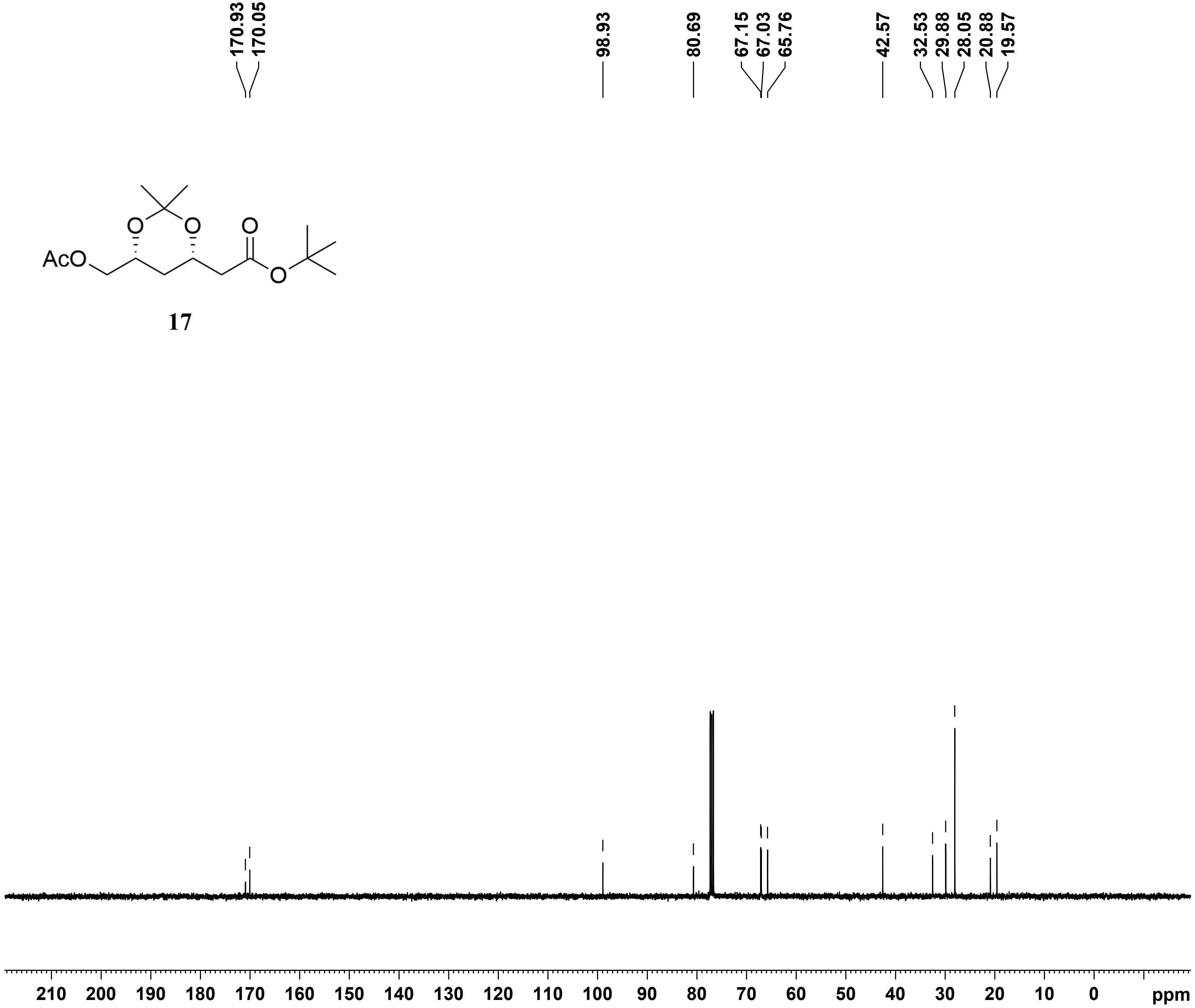

17

NAME	FJJ171218-C13	
EXPNO	1	
PROCNO	1	
Date_	20171218	
Time	14.56	
INSTRUM	spect	
PROBHD	5 mm PABBO BB-	
PULPROG	zgpg30	
TD	65536	
SOLVENT	CDCl3	
NS	81	
DS	4	
SWH	24038.461	Hz
FIDRES	0.366798	Hz
AQ	1.3631988	sec
RG	203	
DW	20.800	usec
DE	6.50	usec
TE	294.8	K
D1	2.00000000	sec
D11	0.03000000	sec
TD0	1	

======== CHANNEL f1 ========

NUC1	13C	
P1	8.50	usec
PL1	-2.00	dB
PL1W	57.32743073	W
SFO1	100.6328888	MHz

======== CHANNEL f2 ========

CPDPRG2	waltz16	
NUC2	1H	
PCPD2	80.00	usec
PL2	-1.00	dB
PL12	13.26	dB
PL13	14.46	dB
PL2W	13.18669796	W
PL12W	0.49446553	W
PL13W	0.37509048	W
SFO2	400.1716007	MHz
SI	32768	
SF	100.6228289	MHz
WDW	EM	
SSB	0	
LB	1.00	Hz
GB	0	
PC	1.40	

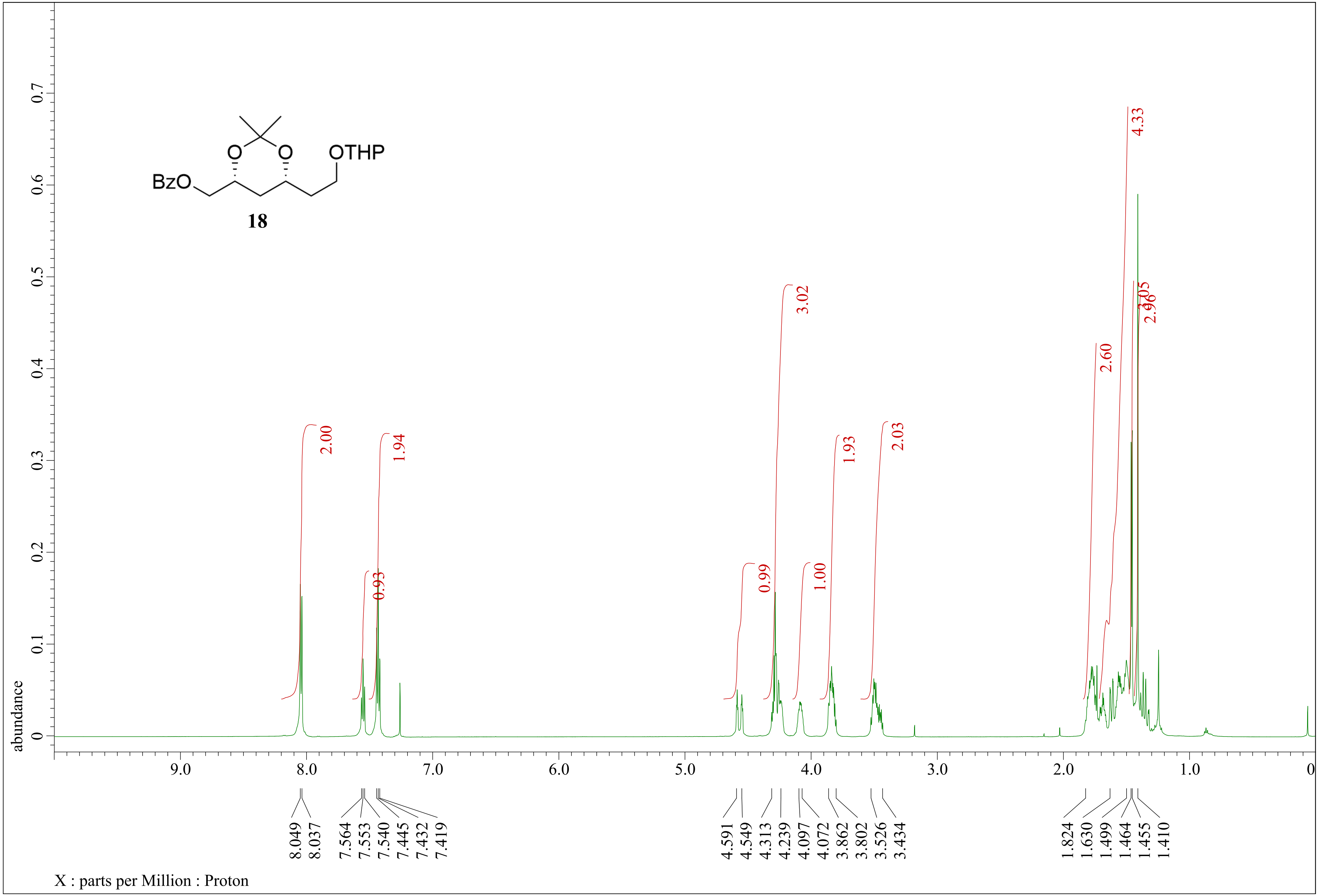

BzO
O
O
OTHP
18
abundance
X : parts per Million : Proton
9.0
8.0
7.0
6.0
5.0
4.0
3.0
2.0
1.0
2.00
1.94
0.93
0.99
3.02
1.00
1.93
2.03
2.60
4.33
3.05
2.96
8.049
8.037
7.564
7.553
7.540
7.445
7.432
7.419
4.591
4.549
4.313
4.239
4.097
4.072
3.862
3.802
3.526
3.434
1.824
1.630
1.499
1.464
1.455
1.410

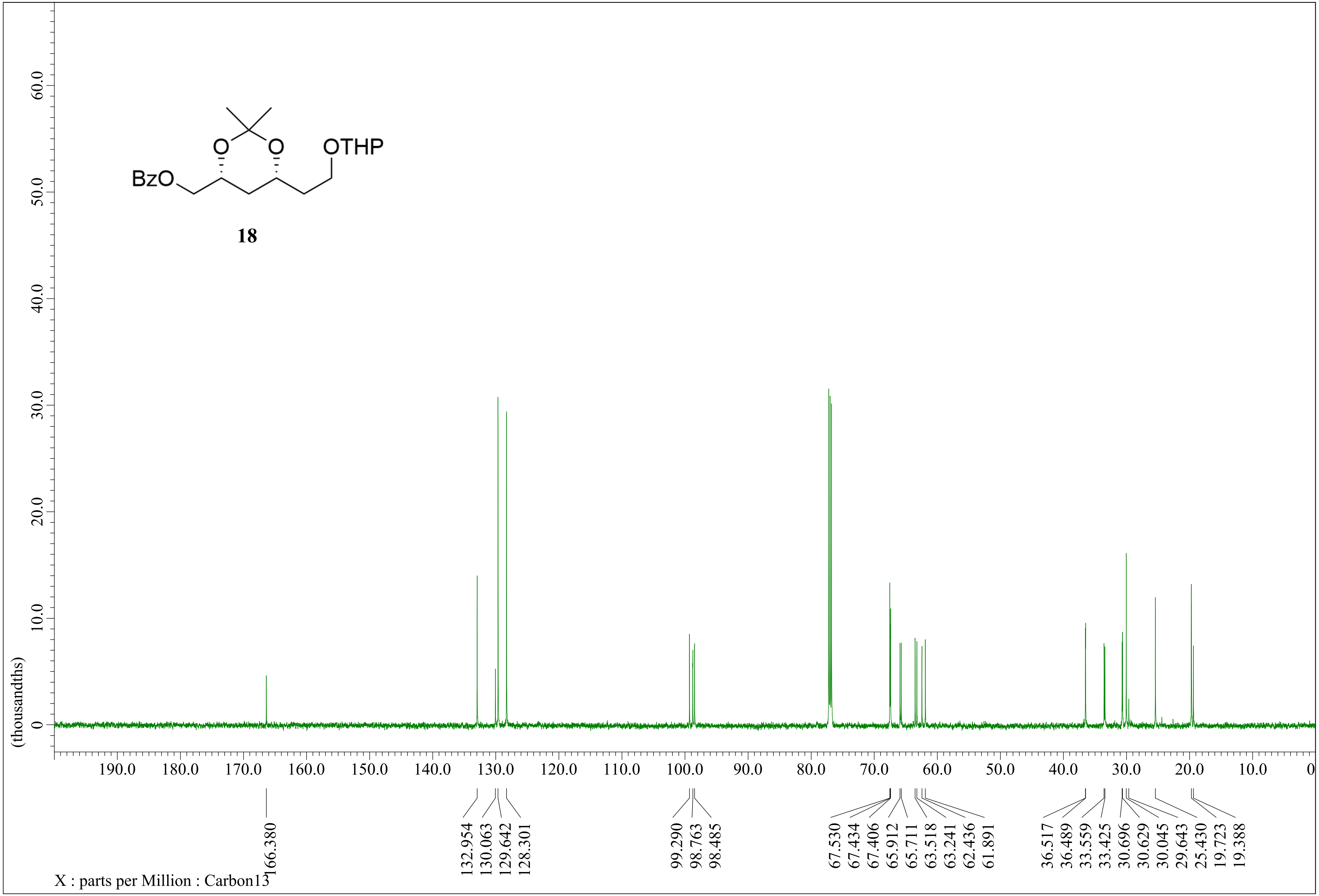

OTHP
BzO
O
O
18
(thousandths)
0
10.0
20.0
30.0
40.0
50.0
60.0
190.0
180.0
170.0
160.0
150.0
140.0
130.0
120.0
110.0
100.0
90.0
80.0
70.0
60.0
50.0
40.0
30.0
20.0
10.0
0
166.380
132.954
130.063
129.642
128.301
99.290
98.763
98.485
67.530
67.434
67.406
65.912
65.711
63.518
63.241
62.436
61.891
36.517
36.489
33.559
33.425
30.696
30.629
30.045
29.643
25.430
19.723
19.388
X : parts per Million : Carbon13

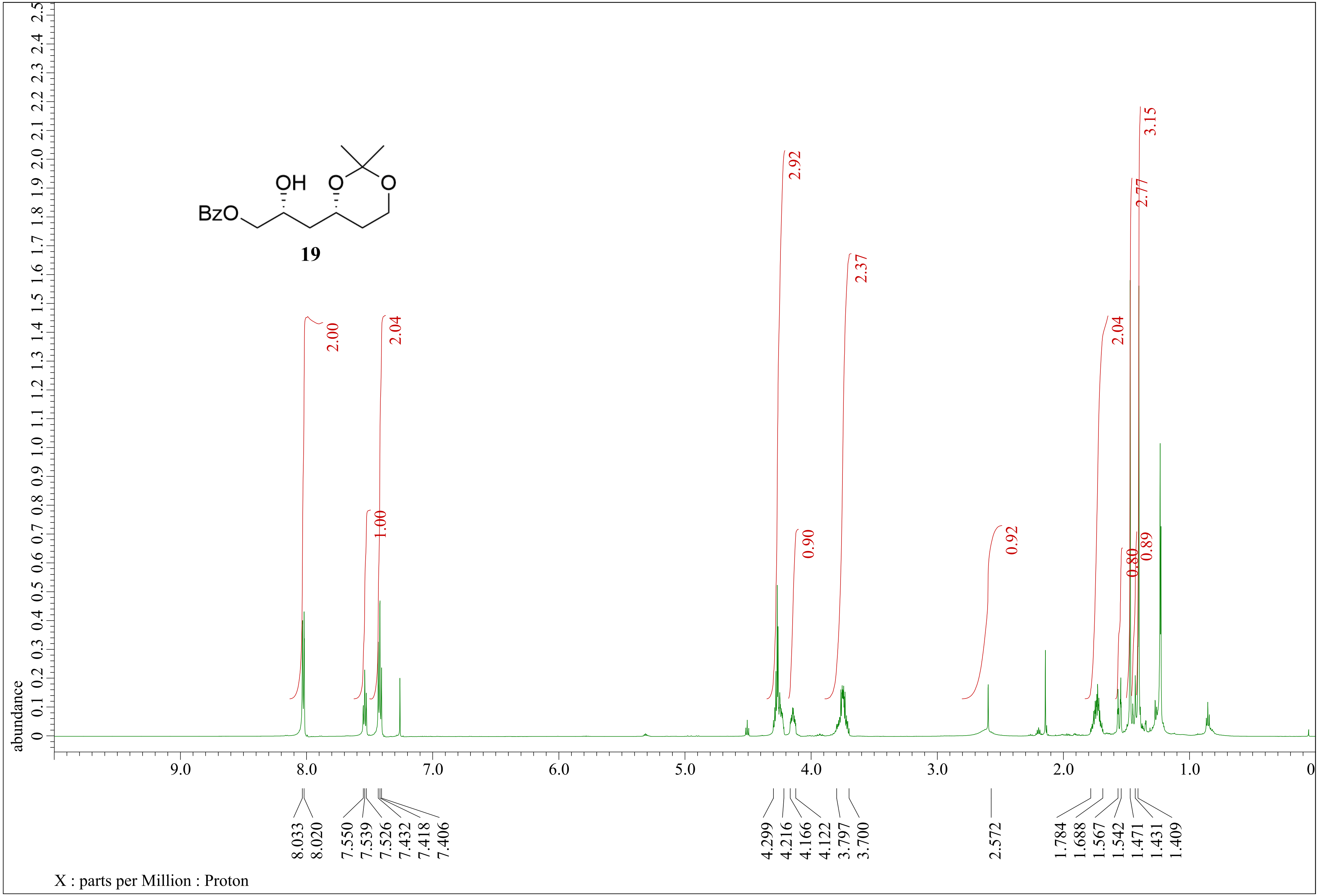
OH
O
O
BzO
19
abundance
X : parts per Million : Proton
9.0
8.0
7.0
6.0
5.0
4.0
3.0
2.0
1.0
2.00
2.04
1.00
2.92
0.90
2.37
0.92
2.04
2.77
3.15
0.80
0.89
8.033
8.020
7.550
7.539
7.526
7.432
7.418
7.406
4.299
4.216
4.166
4.122
3.797
3.700
2.572
1.784
1.688
1.567
1.542
1.471
1.431
1.409

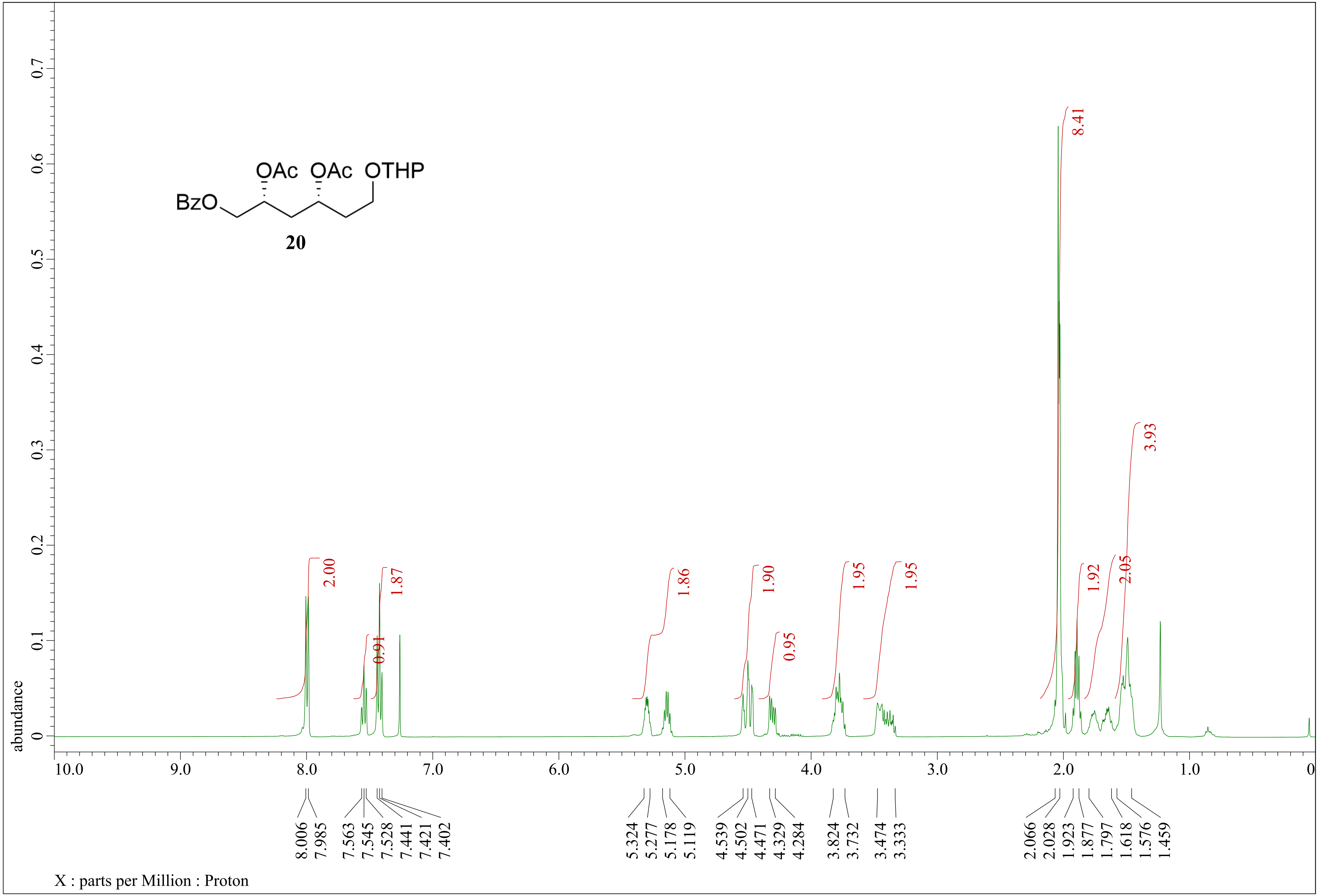

BzO
OAc
OAc
OTHP
20
abundance
X : parts per Million : Proton
8.006
7.985
7.563
7.545
7.528
7.441
7.421
7.402
5.324
5.277
5.178
5.119
4.539
4.502
4.471
4.329
4.284
3.824
3.732
3.474
3.333
2.066
2.028
1.923
1.877
1.797
1.618
1.576
1.459
2.00
0.91
1.87
1.86
1.90
0.95
1.95
1.95
8.41
1.92
2.05
3.93

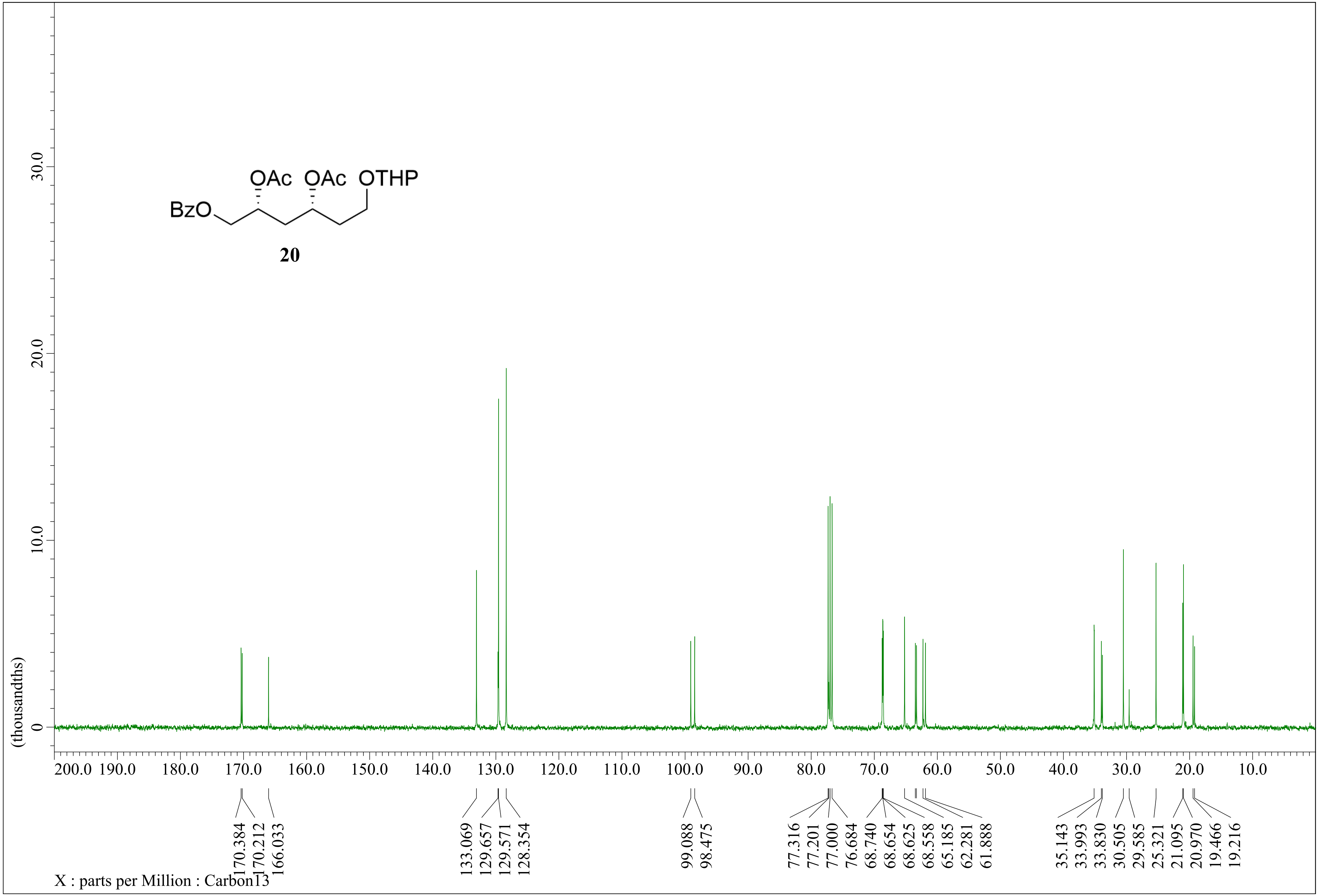
OAc OAc OTHP
BzO
20
(thousandths)
30.0
20.0
10.0
0
200.0 190.0 180.0 170.0 160.0 150.0 140.0 130.0 120.0 110.0 100.0 90.0 80.0 70.0 60.0 50.0 40.0 30.0 20.0 10.0
170.384
170.212
166.033
133.069
129.657
129.571
128.354
99.088
98.475
77.316
77.201
77.000
76.684
68.740
68.654
68.625
68.558
65.185
62.281
61.888
35.143
33.993
33.830
30.505
29.585
25.321
21.095
20.970
19.466
19.216
X : parts per Million : Carbon13

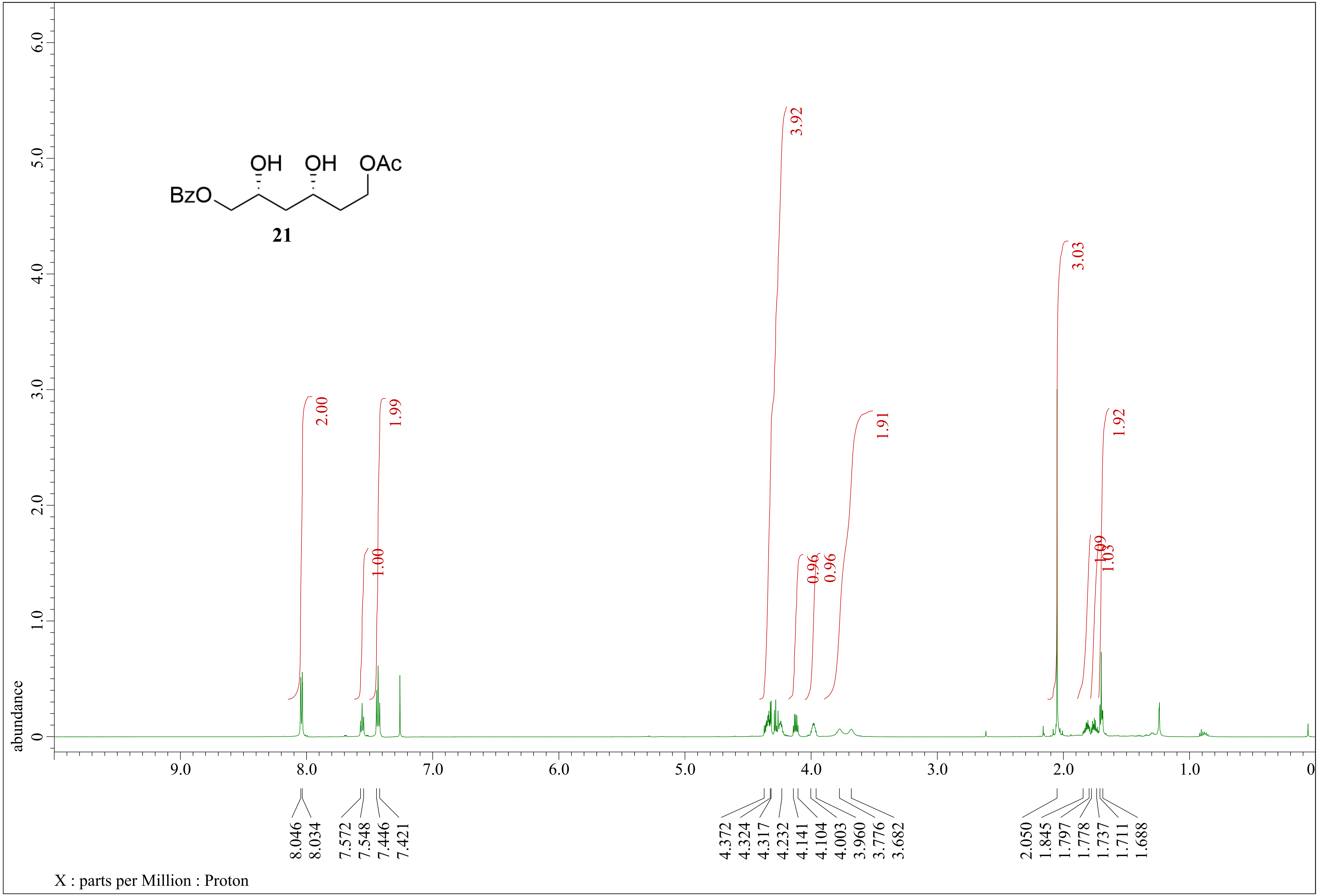

OH
OH
OAc
BzO
21
abundance
X : parts per Million : Proton
3.92
3.03
2.00
1.99
1.91
1.92
1.00
0.96
0.96
1.09
1.03
8.046
8.034
7.572
7.548
7.446
7.421
4.372
4.324
4.317
4.232
4.141
4.104
4.003
3.960
3.776
3.682
2.050
1.845
1.797
1.778
1.737
1.711
1.688

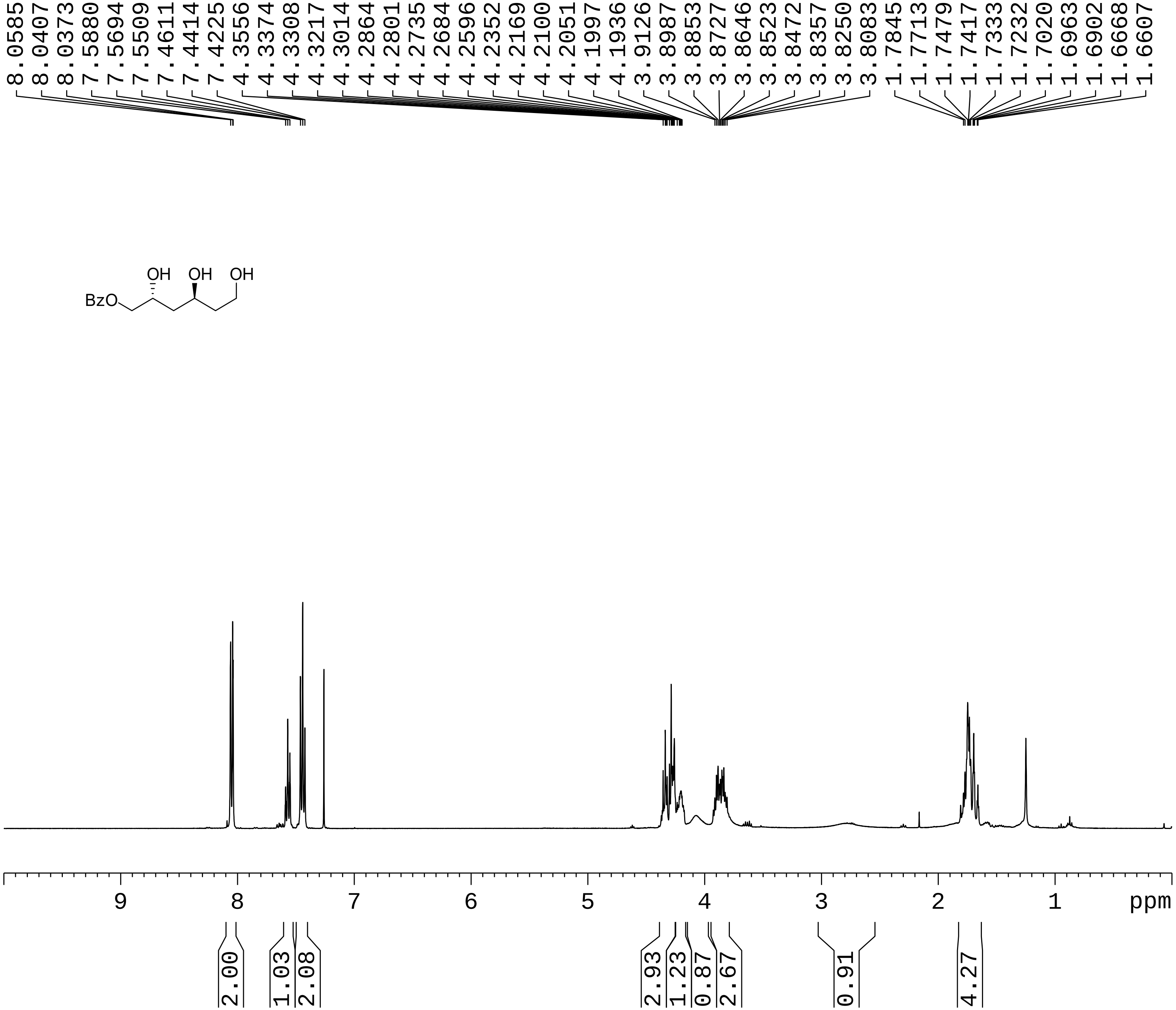

NAME	FJJ170717-2
EXPNO	1
PROCNO	1
Date_	20170722
Time	9.31
INSTRUM	spect
PROBHD	5 mm PABBO BB-
PULPROG	zg30
TD	65536
SOLVENT	CDCl3
NS	16
DS	2
SWH	8223.685 Hz
FIDRES	0.125483 Hz
AQ	3.9846387 sec
RG	161
DW	60.800 usec
DE	6.50 usec
TE	298.7 K
D1	1.00000000 sec
TD0	1

======== CHANNEL f1 ========

NUC1	1H
P1	15.50 usec
PL1	-1.00 dB
PL1W	13.18669796 W
SFO1	400.1724712 MHz
SI	32768
SF	400.1700030 MHz
WDW	EM
SSB	0
LB	0.30 Hz
GB	0
PC	1.00

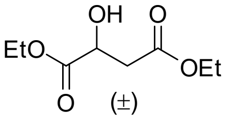

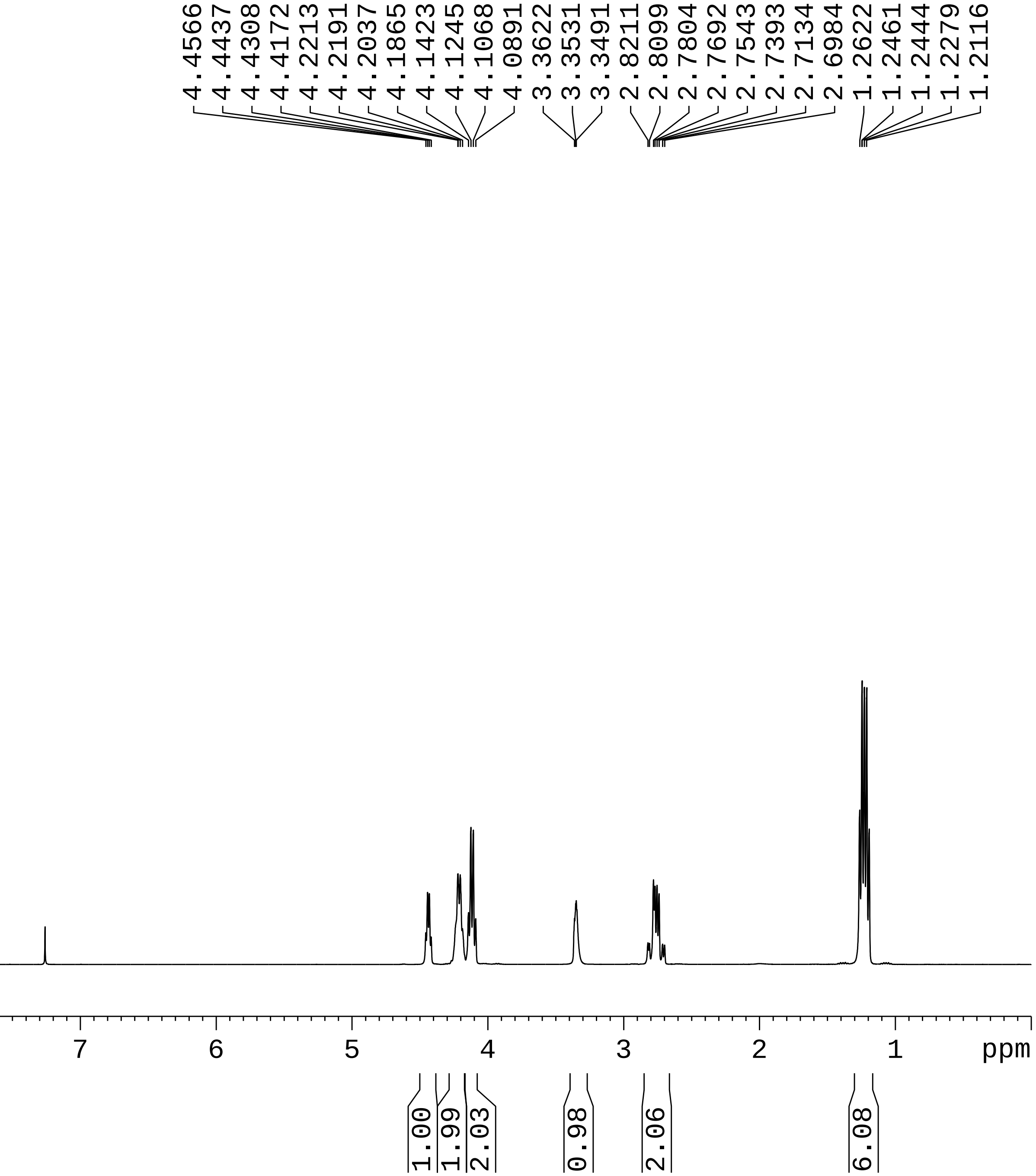


```
NAME       FJJ171128-YUAN
EXPNO                   1
PROCNO                  1
Date_            20171129
Time                16.35
INSTRUM             spect
PROBHD     5 mm PABBO BB-
PULPROG              zg30
TD                  65536
SOLVENT             CDCl3
NS                     16
DS                      2
SWH              8223.685 Hz
FIDRES           0.125483 Hz
AQ              3.9846387 sec
RG                   28.5
DW                 60.800 usec
DE                   6.50 usec
TE                  294.6 K
D1             1.00000000 sec
TD0                     1

======== CHANNEL f1 ========
NUC1                   1H
P1                  15.50 usec
PL1                 -1.00 dB
PL1W          13.18669796 W
SFO1          400.1724712 MHz
SI                  32768
SF            400.1700025 MHz
WDW                    EM
SSB                     0
LB                   0.30 Hz
GB                      0
PC                   1.00
```

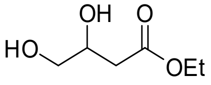

(±)

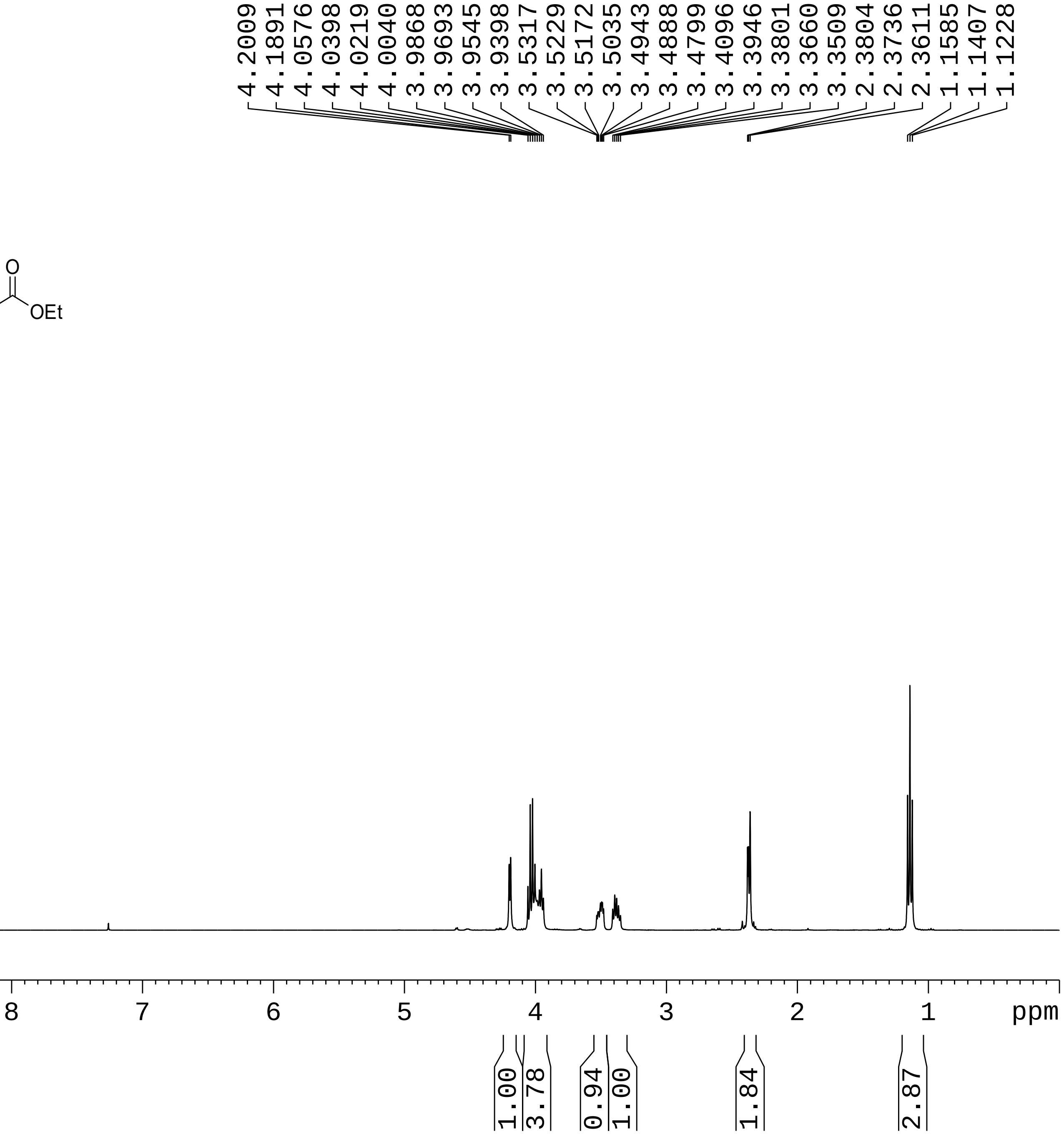

NAME	fjj171203
EXPNO	1
PROCNO	1
Date_	20171204
Time	15.18
INSTRUM	spect
PROBHD	5 mm PABBO BB-
PULPROG	zg30
TD	65536
SOLVENT	CDCl3
NS	16
DS	2
SWH	8223.685 Hz
FIDRES	0.125483 Hz
AQ	3.9846387 sec
RG	20.2
DW	60.800 usec
DE	6.50 usec
TE	297.4 K
D1	1.00000000 sec
TD0	1

======== CHANNEL f1 ========

NUC1	1H
P1	15.50 usec
PL1	-1.00 dB
PL1W	13.18669796 W
SFO1	400.1724712 MHz
SI	32768
SF	400.1700020 MHz
WDW	EM
SSB	0
LB	0.30 Hz
GB	0
PC	1.00

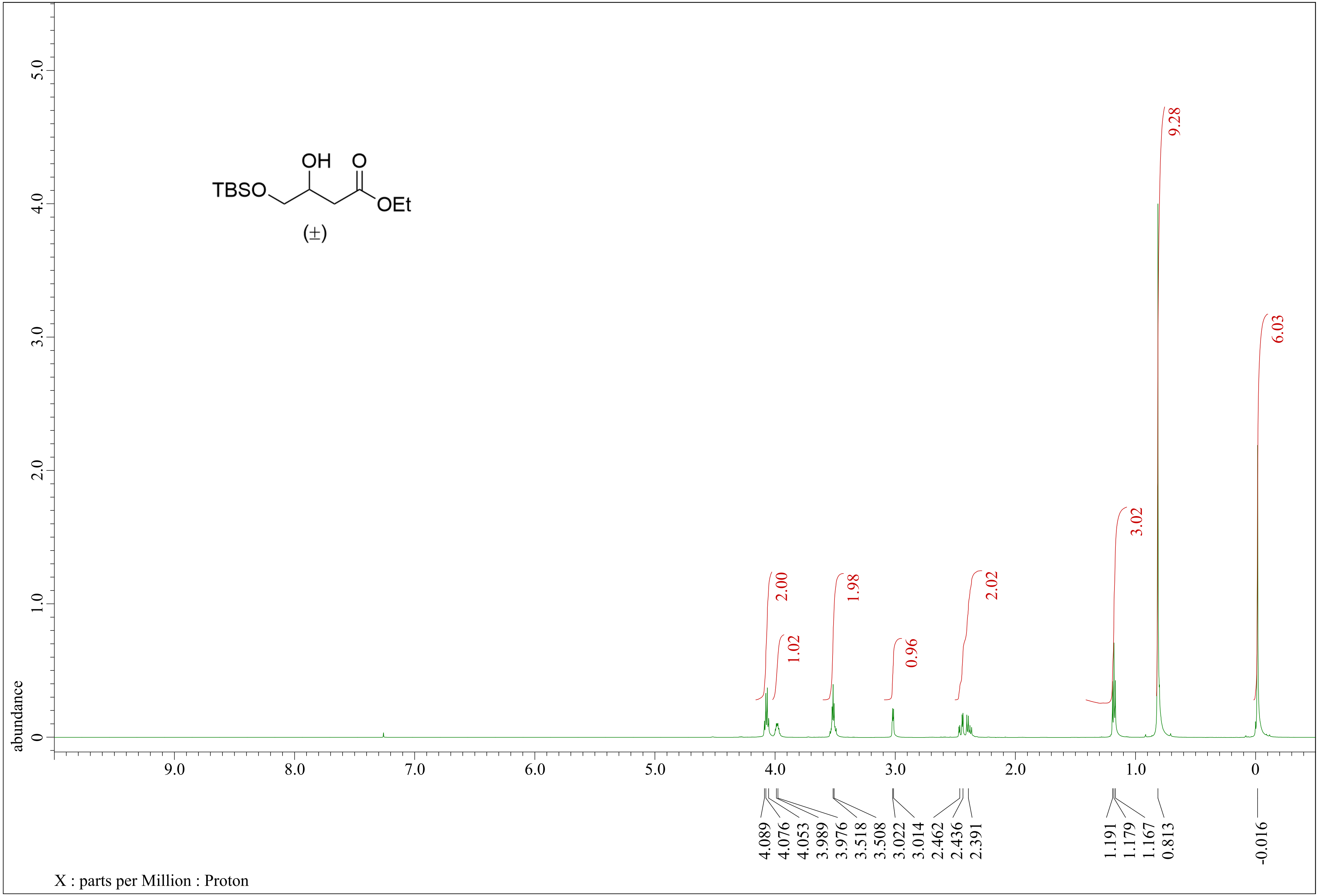

TBSO
OH
O
OEt
(±)
abundance
X : parts per Million : Proton
2.00
1.02
1.98
0.96
2.02
3.02
9.28
6.03
4.089
4.076
4.053
3.989
3.976
3.518
3.508
3.022
3.014
2.462
2.436
2.391
1.191
1.179
1.167
0.813
-0.016

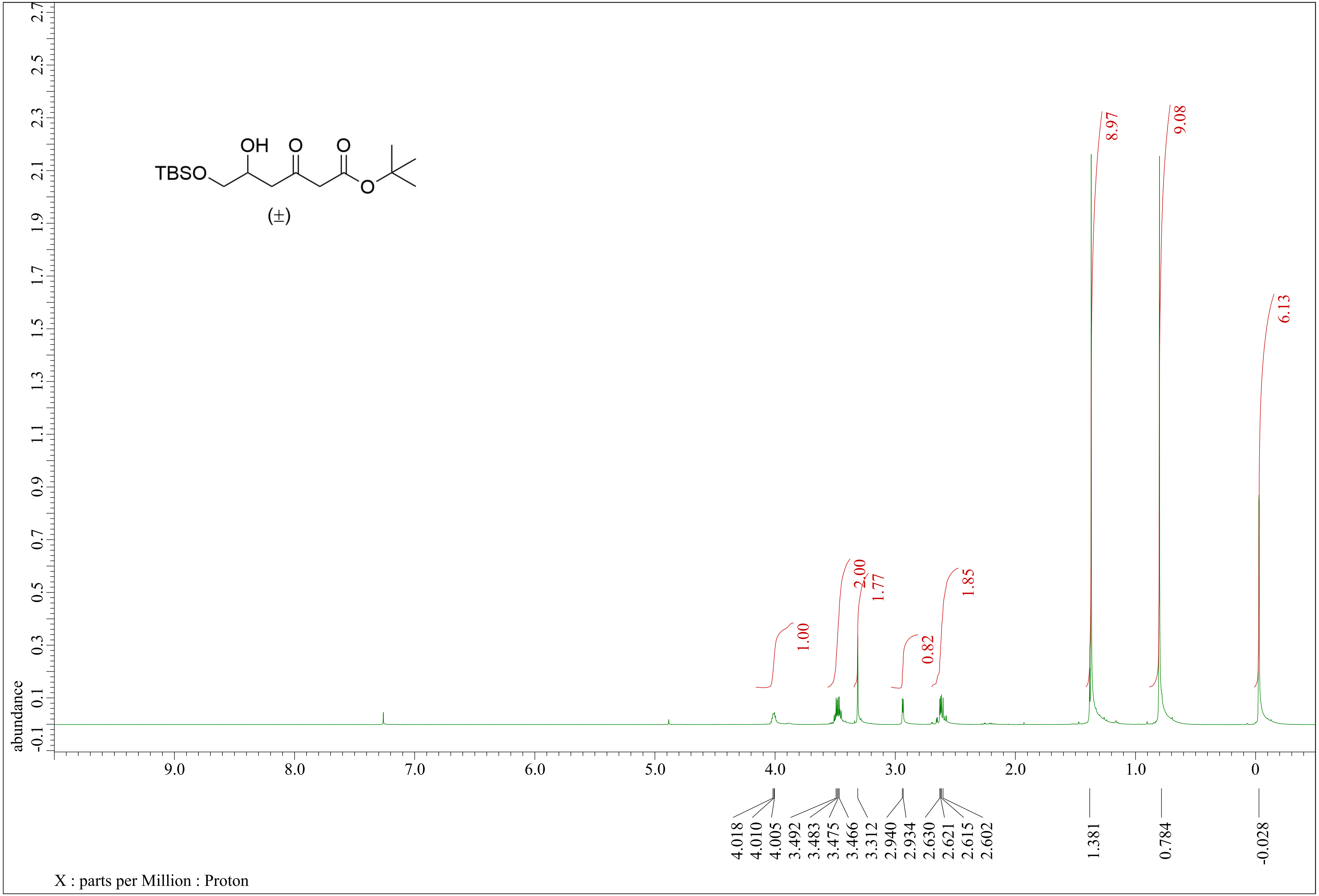

TBSO
OH
O
O
(±)
abundance
X : parts per Million : Proton
1.00
2.00
1.77
0.82
1.85
8.97
9.08
6.13
4.018
4.010
4.005
3.492
3.483
3.475
3.466
3.312
2.940
2.934
2.630
2.621
2.615
2.602
1.381
0.784
-0.028

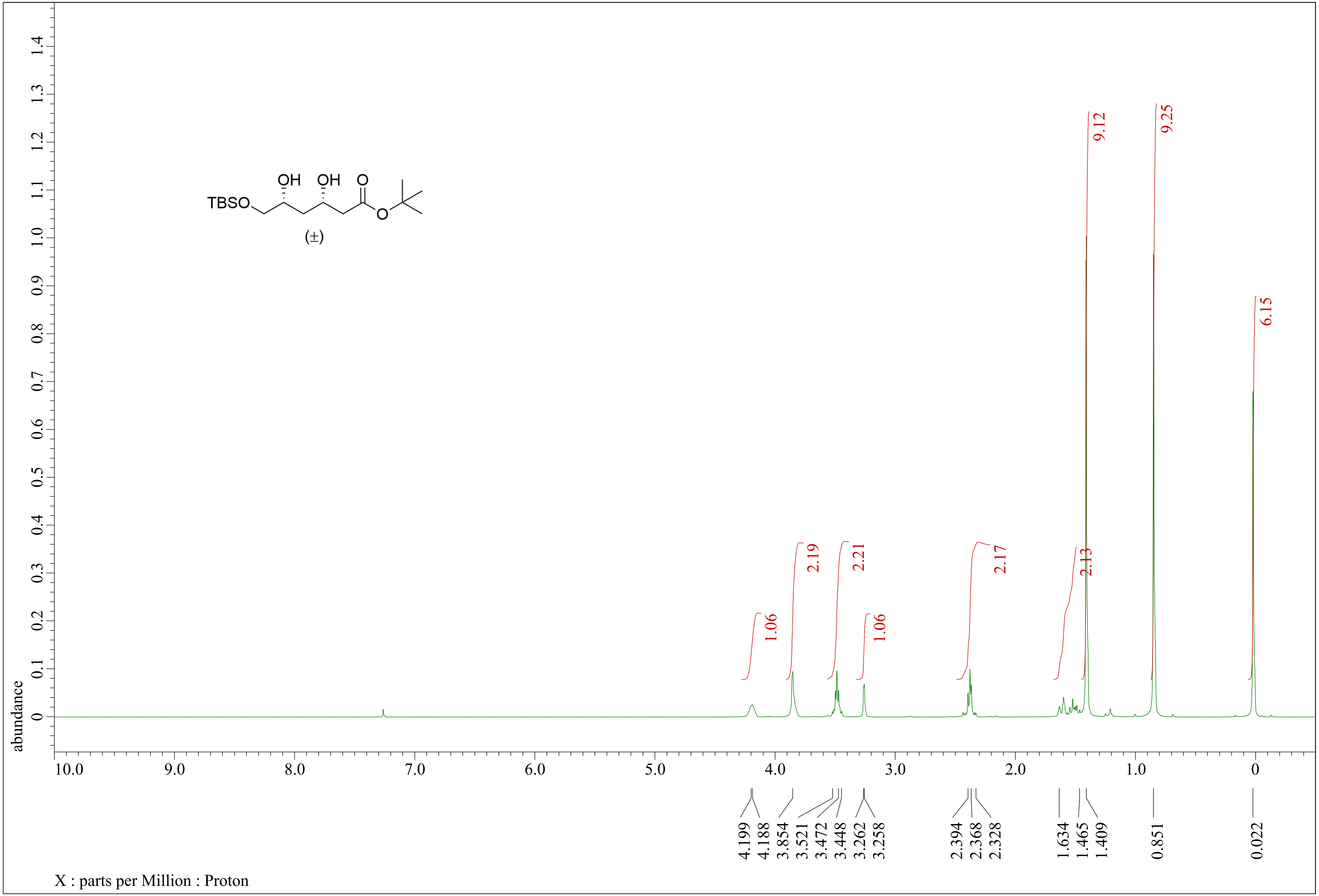
TBSO
OH
OH
O
O
(±)
abundance
X : parts per Million : Proton
1.06
2.19
2.21
1.06
2.17
2.13
9.12
9.25
6.15
4.199
4.188
3.854
3.521
3.472
3.448
3.262
3.258
2.394
2.368
2.328
1.634
1.465
1.409
0.851
0.022

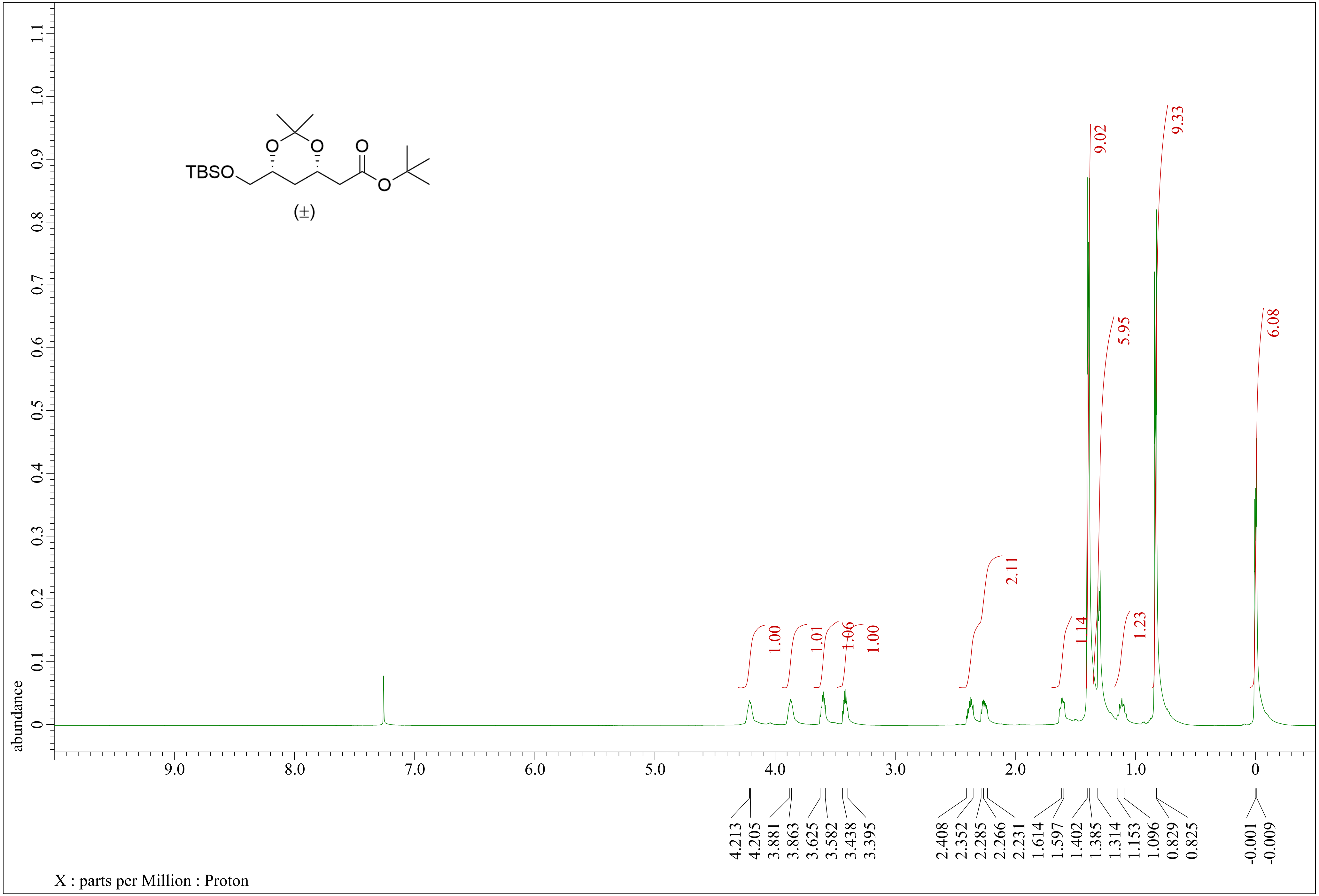
TBSO
(±)
abundance
X : parts per Million : Proton
9.0
8.0
7.0
6.0
5.0
4.0
3.0
2.0
1.0
0
1.00
1.01
1.06
1.00
2.11
1.14
9.02
5.95
1.23
9.33
6.08
4.213
4.205
3.881
3.863
3.625
3.582
3.438
3.395
2.408
2.352
2.285
2.266
2.231
1.614
1.597
1.402
1.385
1.314
1.153
1.096
0.829
0.825
-0.001
-0.009

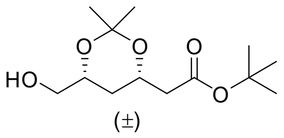

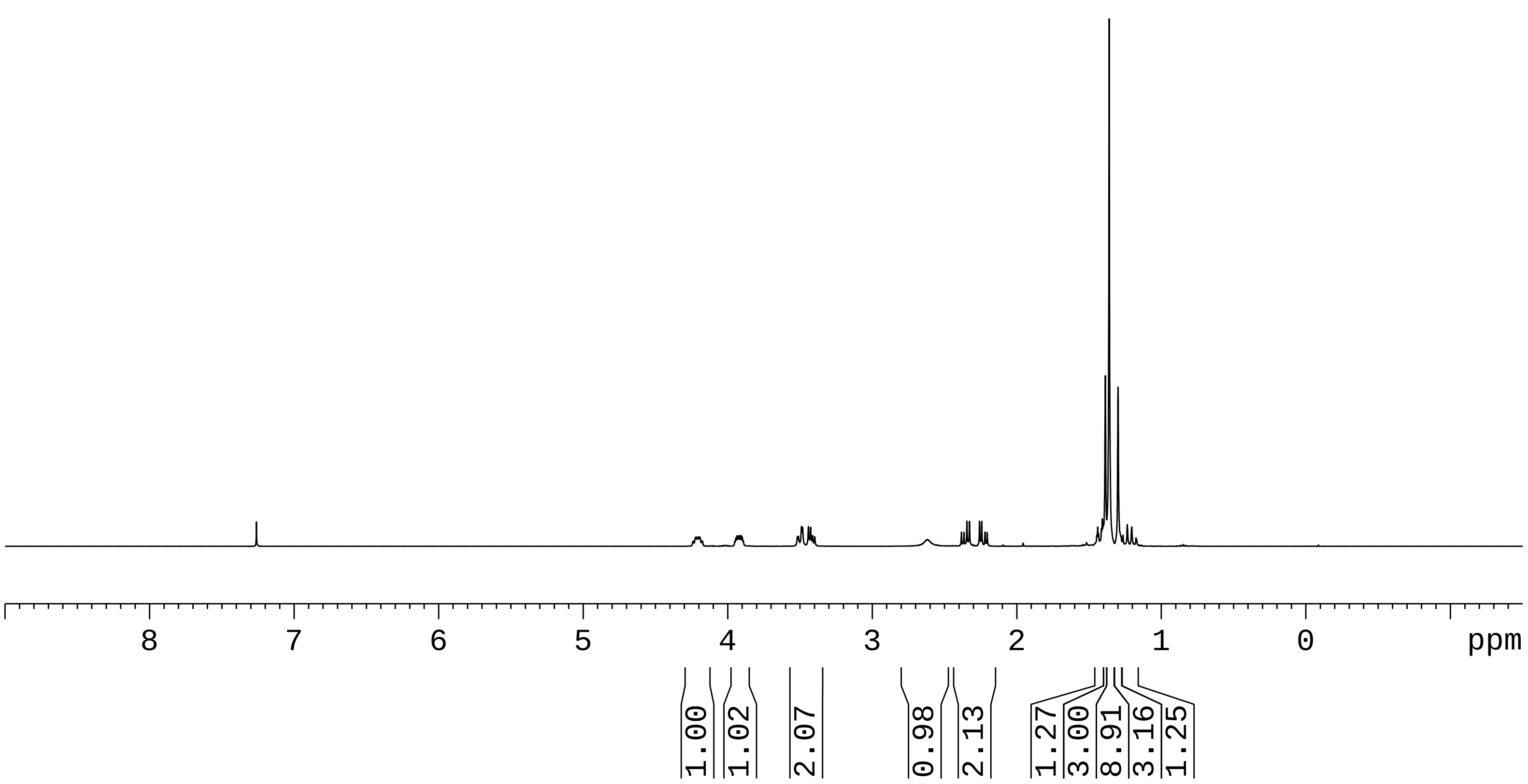

BRUKER

NAME	FJJ171214
EXPNO	1
PROCNO	1
Date_	20171215
Time	14.43
INSTRUM	spect
PROBHD	5 mm PABBO BB-
PULPROG	zg30
TD	65536
SOLVENT	CDCl3
NS	16
DS	2
SWH	8223.685 Hz
FIDRES	0.125483 Hz
AQ	3.9846387 sec
RG	22.6
DW	60.800 usec
DE	6.50 usec
TE	294.1 K
D1	1.00000000 sec
TD0	1

======== CHANNEL f1 ========

NUC1	1H
P1	15.50 usec
PL1	-1.00 dB
PL1W	13.18669796 W
SFO1	400.1724712 MHz
SI	32768
SF	400.1700023 MHz
WDW	EM
SSB	0
LB	0.30 Hz
GB	0
PC	1.00

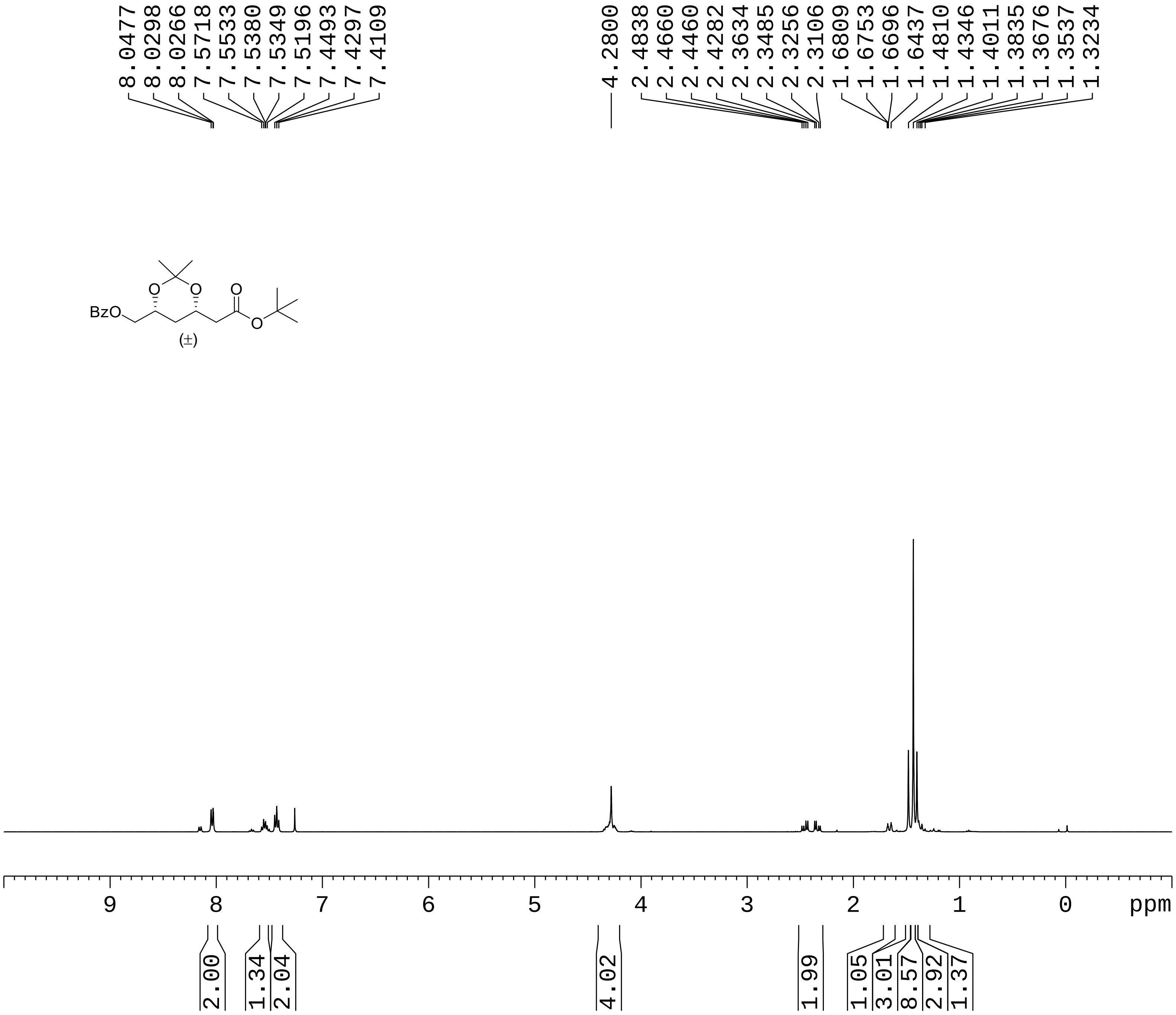

BRUKER

NAME	FJJ171215
EXPNO	1
PROCNO	1
Date_	20171215
Time	19.29
INSTRUM	spect
PROBHD	5 mm PABBO BB-
PULPROG	zg30
TD	65536
SOLVENT	CDCl3
NS	16
DS	2
SWH	8223.685 Hz
FIDRES	0.125483 Hz
AQ	3.9846387 sec
RG	50.8
DW	60.800 usec
DE	6.50 usec
TE	292.8 K
D1	1.00000000 sec
TD0	1

======== CHANNEL f1 ========

NUC1	1H
P1	15.50 usec
PL1	-1.00 dB
PL1W	13.18669796 W
SFO1	400.1724712 MHz
SI	32768
SF	400.1700028 MHz
WDW	EM
SSB	0
LB	0.30 Hz
GB	0
PC	1.00

==== Shimadzu LCsolution Analysis Report ====

E:\data\901\FLF\Fjj170909-xiaoxuan1 AD 21.lcd

Acquired by	: Admin
Sample Name	: Fjj170909-xiaoxuan1
Sample ID	:
Vail #	:
Injection Volume	: 1 uL
Data File Name	: Fjj170909-xiaoxuan1 AD 21.lcd
Method File Name	: method1.lcm
Batch File Name	:
Report File Name	: Default.lcr
Data Acquired	: 2017/9/20 10:48:27
Data Processed	: 2017/9/20 11:02:32

<Chromatogram>

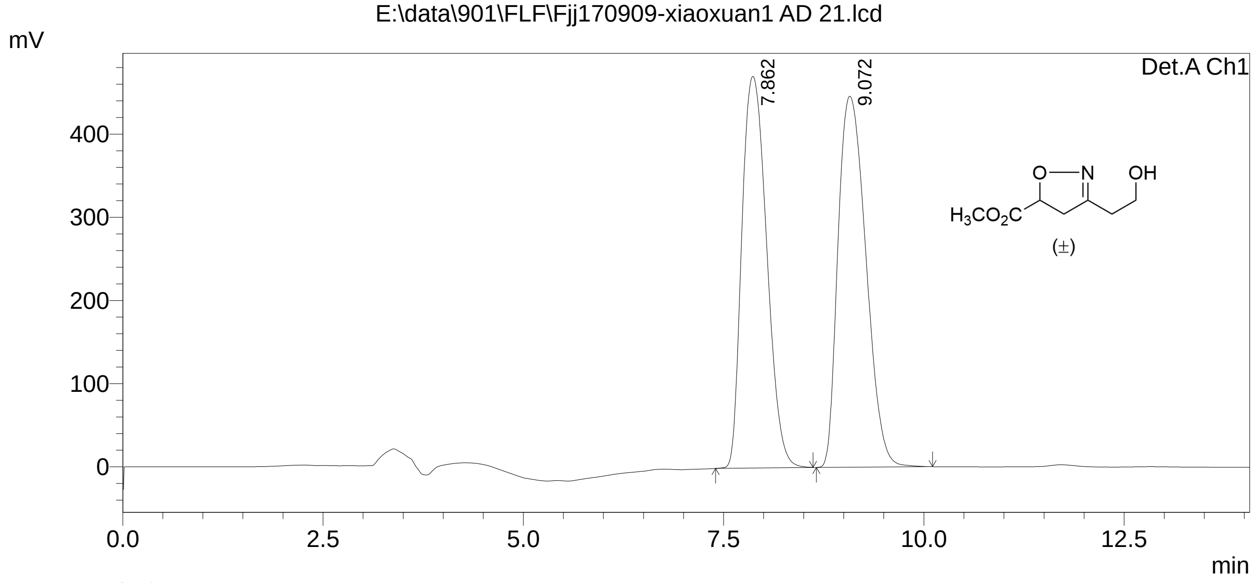

1 Det.A Ch1/225nm

PeakTable

Detector A Ch1 225nm

Peak#	Ret. Time	Area	Height	Area %	Height %
1	7.862	10179161	470877	48.473	51.351
2	9.072	10820335	446096	51.527	48.649
Total		20999497	916973	100.000	100.000

==== Shimadzu LCsolution Analysis Report ====

E:\data\901\FLF\Fjj170909-yibingjiyes20% AD 26.lcd

Acquired by	: Admin
Sample Name	: Fjj170909-yibingjiyes20%
Sample ID	:
Vail #	:
Injection Volume	: 1 uL
Data File Name	: Fjj170909-yibingjiyes20% AD 26.lcd
Method File Name	: method1.lcm
Batch File Name	:
Report File Name	: Default.lcr
Data Acquired	: 2017/9/20 16:24:16
Data Processed	: 2017/9/20 16:41:01

<Chromatogram>

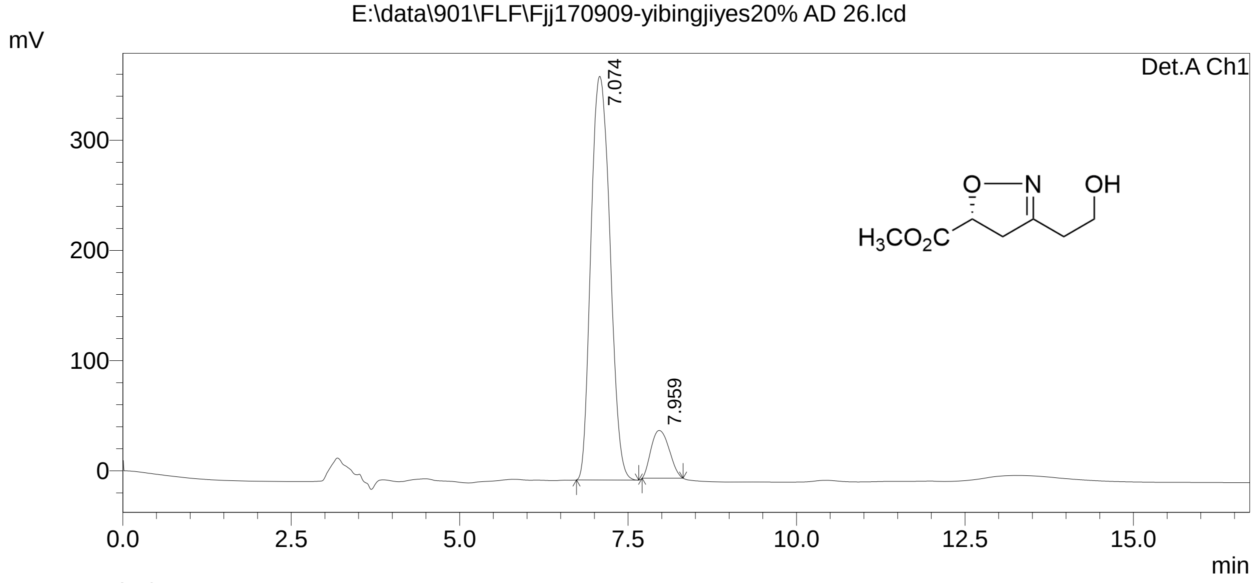

1 Det.A Ch1/225nm

PeakTable

Detector A Ch1 225nm

Peak#	Ret. Time	Area	Height	Area %	Height %
1	7.074	7244825	366410	89.902	89.429
2	7.959	813724	43314	10.098	10.571
Total		8058549	409724	100.000	100.000

==== Shimadzu LCsolution Analysis Report ====

E:\data\901\Fjj HPLC\原数据\FJJ171215-XX-(OD 10% 0.5mL)254nm3.lcd

Acquired by	: Admin
Sample Name	: FJJ171215-XX-(OD 10% 0.5mL)254nm
Sample ID	:
Vail #	:
Injection Volume	: 1 uL
Data File Name	: FJJ171215-XX-(OD 10% 0.5mL)254nm3.lcd
Method File Name	: method1.lcm
Batch File Name	:
Report File Name	: Default.lcr
Data Acquired	: 2017/12/16 18:42:01
Data Processed	: 2017/12/16 19:50:00

<Chromatogram>

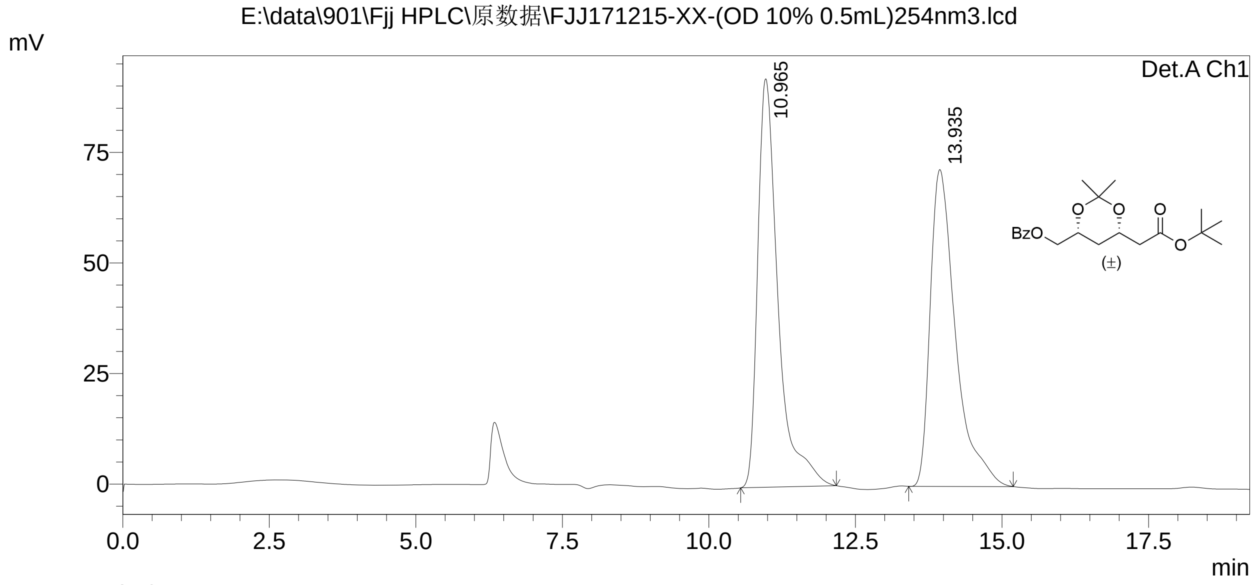

1 Det.A Ch1/254nm

PeakTable

Detector A Ch1 254nm

Peak#	Ret. Time	Area	Height	Area %	Height %
1	10.965	2232473	92355	50.854	56.314
2	13.935	2157511	71644	49.146	43.686
Total		4389983	163999	100.000	100.000

==== Shimadzu LCsolution Analysis Report ====

E:\data\901\FLF\FJJ171215-SX-n(OD 10% 0.5mL)254nm4.lcd

Acquired by	: Admin
Sample Name	: FJJ171215-SX-n(OD 10% 0.5mL)254nm
Sample ID	:
Vail #	:
Injection Volume	: 1 uL
Data File Name	: FJJ171215-SX-n(OD 10% 0.5mL)254nm4.lcd
Method File Name	: method1.lcm
Batch File Name	:
Report File Name	: Default.lcr
Data Acquired	: 2017/12/16 19:03:28
Data Processed	: 2017/12/16 19:24:11

<Chromatogram>

E:\data\901\FLF\FJJ171215-SX-n(OD 10% 0.5mL)254nm4.lcd

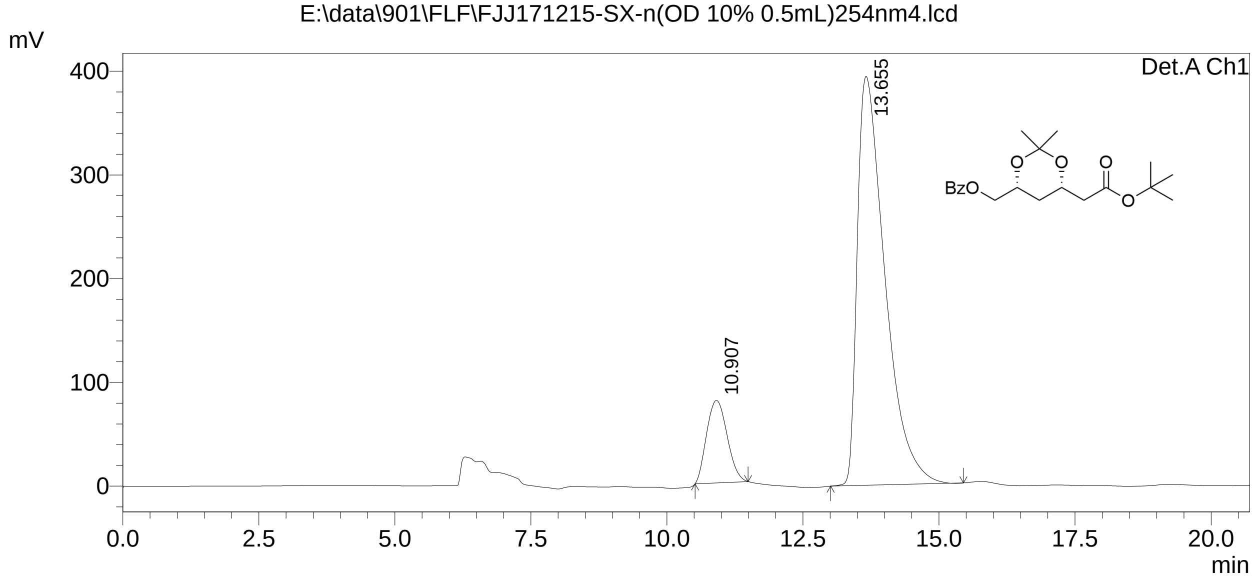

1 Det.A Ch1/254nm

PeakTable

Detector A Ch1 254nm

Peak#	Ret. Time	Area	Height	Area %	Height %
1	10.907	2097109	79502	13.089	16.779
2	13.655	13924276	394315	86.911	83.221
Total		16021384	473816	100.000	100.000
